# Gene editing and CRISPR in the clinic: current and future perspectives

**DOI:** 10.1042/BSR20200127

**Published:** 2020-04-09

**Authors:** Matthew P. Hirakawa, Raga Krishnakumar, Jerilyn A. Timlin, James P. Carney, Kimberly S. Butler

**Affiliations:** 1 Systems Biology, Sandia National Laboratories, Livermore, CA 94551, U.S.A.; 2 Molecular and Microbiology, Sandia National Laboratories, Albuquerque, NM 87185, U.S.A.; 3 Advanced Materials Laboratory, Sandia National Laboratories, Albuquerque, NM 87185, U.S.A.

**Keywords:** clinical trial, CRISPR, gene activation, genome editing, transcription activator-like effector nucleases, zinc finger nuclease

## Abstract

Genome editing technologies, particularly those based on zinc-finger nucleases (ZFNs), transcription activator-like effector nucleases (TALENs), and CRISPR (clustered regularly interspaced short palindromic repeat DNA sequences)/Cas9 are rapidly progressing into clinical trials. Most clinical use of CRISPR to date has focused on *ex vivo* gene editing of cells followed by their re-introduction back into the patient. The *ex vivo* editing approach is highly effective for many disease states, including cancers and sickle cell disease, but ideally genome editing would also be applied to diseases which require cell modification *in vivo*. However, *in vivo* use of CRISPR technologies can be confounded by problems such as off-target editing, inefficient or off-target delivery, and stimulation of counterproductive immune responses. Current research addressing these issues may provide new opportunities for use of CRISPR in the clinical space. In this review, we examine the current status and scientific basis of clinical trials featuring ZFNs, TALENs, and CRISPR-based genome editing, the known limitations of CRISPR use in humans, and the rapidly developing CRISPR engineering space that should lay the groundwork for further translation to clinical application.

## Introduction

Recent advances in biotechnology have revolutionized our ability to understand the genetic basis of human health. The advent of next-generation sequencing technologies and high throughput DNA microarrays have provided crucial tools to define the landscape of human genetic variation through large, international collaborative efforts [1,2]. Today, millions of human reference single nucleotide polymorphisms (SNPs) and structural variants from individuals worldwide are cataloged and publicly available for further investigation [3]. The ability to quickly and thoroughly genotype individuals has enabled researchers to identify numerous genetic loci involved in common and complex diseases through genome-wide association studies [4,5]. Identifying genetic signatures associated with a wide variety of diseases in patients has been helpful in providing appropriate treatments and lifestyle changes to mitigate disease symptoms and improve the quality of life. With the rapid emergence of new genome editing tools, long-lasting or permanent mitigation of genetic diseases through selective modification of the human genome is now possible resulting in immense potential to improve human health.

In this review, we discuss the current state of gene editing technologies and their use as treatments for human disease. We place special emphasis on CRISPR (clustered regularly interspaced short palindromic repeat DNA sequences)-based technologies, as they are quickly transforming the state of life science research around the world and progressing into clinical trials. The initial wave of modern-day genome editors (zinc-finger nucleases (ZFNs) and transcription activator-like effector nucleases (TALENs)) have been evaluated in clinical trials to some extent, but the use of these technologies has been limited due to the need for difficult and laborious engineering of a new version of the editing protein for each new target in the genome [6–8]. Most recently, CRISPR, a naturally occurring prokaryotic immune system that evolved across diverse bacteria and archaea to protect themselves from invading viruses [9,10] has been re-purposed into powerful gene editing tools that function efficiently in diverse organisms including humans [11]. In brief, CRISPR-editing involves the use of CRISPR-associated (Cas) enzymes that target and cleave specific nucleic acid sequences. There are numerous identified CRISPR systems, each of which have distinct nucleic acid binding requirements and enzymatic activities, however the majority of CRISPR-based applications to date utilize Cas9 from *Streptococcus pygogenes* (spCas9) [12]. To target specific DNA sequences, Cas9 utilizes a CRISPR RNA (crRNA) with a 20-nucleotide complimentary sequence to the target sequence, and a trans-activating crRNA (tracrRNA) scaffold that is recognized by the Cas9 protein [13–15]. Importantly, the crRNA and tracrRNA can be fused to form a single guide RNA (sgRNA) chimera that retains the ability to target and cleave specific nucleic acid target sequences [16]. In contrast to early ZFN and TALEN-based editors, CRISPR-based systems require only alteration of the 20-nucleotide target sequence of the sgRNA in order to specifically target a new site in the genome, making the transition between gene targets far more efficient. Because of this, CRISPR-based systems are quickly transforming the state of life science research around the world and progressing into clinical trials. Comprehensive reviews of the history, function, and diversity of ZFN, TALEN, and CRISPR editors have been the subject of many prior reviews and the reader is referred there for introductory material about the function of these powerful editing technologies [6,12,17].

In this review, we will first discuss the state of gene editing technologies and their use as treatments for human disease with a specific focus on CRISPR-based therapies that are currently being tested in ongoing clinical trials. Second, we will present the known limitations for *in vivo* use of gene editors which include off-target effects, delivery issues, and immunogenicity of gene editing molecules. Given the rapid progression of gene editing tools, there are a number of solutions in the research and pre-clinical stages of development that have future potential to address these limitations for clinical use in humans. To conclude this review, we will discuss newly developed technologies that hold promise to address the limitations of current gene editors for clinical use that include the development of new delivery vehicles to direct gene editors to specific tissues, hyperaccurate CRISPR systems that decrease off-target effects, and gene editing tools that modulate the reversible control of gene expression and epigenetics.

## Clinical trials with gene editors

**Figure 1:**
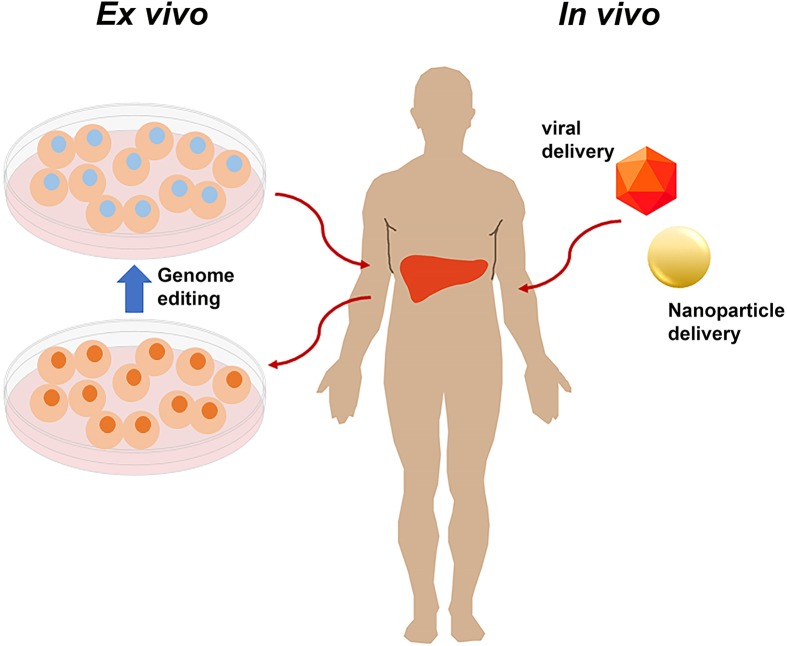
Genome editors can be used therapeutically in several ways, and both *ex vivo* and *in vivo* delivery for somatic genome editing have advanced to clinical trial *Ex vivo*: cells can be extracted from the patient or donor modified in the laboratory and then infused into the patient. *In vivo*: delivery vehicles, including viral vectors and nanoparticles can be loaded with the genome editor and then injected into the patient either systemically, which results in liver editing primarily, or into the location of interest, for example, the eye.

The U.S. clinical trials database (clinicaltrials.gov) contains all studies which meet the definition of an ‘applicable clinical trial’ initiated on or after 27 September 2007 or continuing beyond 26 December 2007. In addition to trials required to register, voluntary registration is also accepted; studies conducted outside U.S.A., and those which may meet one of the conditions in the future, often register voluntarily. We searched the U.S. clinical trials database (01/01/2020) for any trial containing at least one of the following terms: CRISPR, Cas9, Cas12, Cas13, ZFN, zinc finger, gene edit, gene modification, and genome edit. Trials that did not use the genome editor as part of the therapeutic intervention were excluded from the analysis; these included trials to create cell lines from patients using Cas9; use of patient cells to develop therapeutic strategies, but where the cells were not used as a therapeutic themselves; CRISPR use for genome sequencing; and surveys of opinions regarding human gene editing. This search identified 41 trials utilizing genome editing agents including ZFNs, TALENs, and CRISPR/Cas9 for therapeutic interventions, no studies utilizing Cas12 or Cas13 have been registered (Table 1). Genome editing agents have clinically been utilized in two ways (Figure 1): cells can be removed from the patient or donor and modified outside the body (*ex vivo*) followed by reinfusion into the patient or the genome editor itself can be injected into the patient (*in vivo).* Of the registered trials, 37 were *ex vivo* delivery and only 8 were *in vivo* delivery.

**Table 1:** Interventional trials with genome editors

Delivery vector	Nuclease	Phase	Target gene and effect	Disease	*Ex vivo/in vivo*	Intervention	Sponsor organization	Country	NCT number	Date posted
Adenoviral vectors	ZFN	I	CCR5 knockout	HIV	*Ex vivo*	Modified CD4^+^ T cells	University of Pennsylvania	U.S.A.	NCT00842634	2/12/2009
	ZFN	I	CCR5 knockout	HIV	*Ex vivo*	Modified CD4^+^ T cells	Sangamo Biosciences	U.S.A.	NCT01044654	1/8/2010
	ZFN	I/II	CCR5 knockout	HIV	*Ex vivo*	Modified CD4^+^ T cells	Sangamo Biosciences	U.S.A.	NCT01252641	12/3/2010
	ZFN	I/II	CCR5 knockout	HIV	*Ex vivo*	Modified CD4^+^ T cells	Sangamo Biosciences	U.S.A.	NCT01543152	3/2/2012
	ZFN	I/II	CCR5 knockout	HIV	*Ex vivo*	Modified CD4^+^ T cells	Case Western Reserve University	U.S.A.	NCT03666871	9/12/2018
AAV vectors	ZFN	I	Factor IX addition al albumin locus	Hemophilia B	*In vivo*	ZFN-mediated addition of Factor IX gene to the albumin locus of hepatocytes	Sangamo Biosciences	U.S.A.	NCT02695160	3/1/2016
	ZFN	I	α-L-iduronidase (IDUA) addition at albumin locus	MPS type I	*In vivo*	ZFN-mediated addition of *IDUA* gene to the albumin locus of hepatocytes	Sangamo Biosciences	U.S.A.	NCT02702115	3/8/2016
	ZFN	I	Iduronate 2-sulfatase (IDS) addition at albumin locus	MPS type II	*In vivo*	ZFN-mediated addition of *IDS* gene to the albumin locus of hepatocytes	Sangamo Biosciences	U.S.A.	NCT03041324	2/2/2017
	Cas9	I	Removal of alternative splice site in CEP290	Leber congenital amaurosis 10	*In vivo*	ZFN-mediated removal of intronic alternative splice site in retinal cells	Allergan and Editas Medicine, Inc.	U.S.A.	NCT03872479	3/13/2019
Electroporation (mRNA)	ZFN	I/II	CCR5 knockout	HIV	*Ex vivo*	Modified CD4^+^ T cells	Sangamo Biosciences	U.S.A.	NCT02225665	8/26/2014
	ZFN	I	CCR5 knockout	HIV	*Ex vivo*	Modified CD4^+^ T cells	University of Pennsylvania	U.S.A.	NCT02388594	3/17/2015
	ZFN	I	CCR5 knockout	HIV	*ex vivo*	Modified CD34^+^ hematopoietic stem cells	City of Hope Medical Center	U.S.A.	NCT02500849	7/17/2015
	ZFN	I/II	Disrupt the erythroid enhancer in B-cell lymphoma/leukemia 11A (BCL11A)	β-thalassemia	*Ex vivo*	Modified hematopoietic stem cells	Sangamo Biosciences	U.S.A.	NCT03432364	2/14/2018
	ZFN	I	CCR5 knockout	HIV	*Ex vivo*	Modified T cells with ZFN-mediated CCR5 deletion as well as the addition of CD4 CAR receptor and modified CXCR4 expression	University of Pennsylvania	U.S.A.	NCT03617198	8/6/2018
	ZFN	I/II	Disrupt B-cell lymphoma/leukemia 11A (BCL11A)	sickle cell anemia	*Ex vivo*	Modified hematopoietic stem cells	Bioverativ	U.S.A.	NCT03653247	8/31/2018
	TALEN	I	TCRα, TCRβ, CD52 knockout	Advanced lymphoid malignancy	*Ex vivo*	CD19-CAR modified T cells with CAR delivered by lentivirus and TALEN knockout CD52 and TCR to create universal T cells	Institut de Recherches Internationales Servier	U.K., U.S.A., France	NCT02746952	4/21/2016
	TALEN	I	TCRα, TCRβ, CD52 knockout	Refractory B-ALL	*Ex vivo*	CD19-CAR modified T cells with CAR delivered by lentivirus and TALEN knockout CD52 and TCR to create universal T-cells	Institut de Recherches Internationales Servier	U.K., Belgium, France, U.S.A.	NCT02808442	6/21/2016
	TALEN	I	Programmed cell death 1 (PD-1) and CD52 knockout	Acute myeloid leukemia	*Ex vivo*	CD123-CAR modified T cells with CAR delivered by lentivirus and TALEN-mediated knockouts	Cellectis S.A.	U.S.A.	NCT03190278	6/16/2017
	TALEN	I	Programmed cell death 1 (PD-1) and CD52 knockout	Blastic plasmacytoid dendritic cell neoplasm	*Ex vivo*	CD123-CAR modified T cells with CAR delivered by lentivirus and TALEN-mediated knockouts	Cellectis S.A.	U.S.A.	NCT03203369	6/29/2017
	TALEN	I	Programmed cell death 1 (PD-1) and CD52 knockout	Acute myeloid leukemia	*Ex vivo*	CD123-CAR modified T cells with CAR delivered by lentivirus and TALEN-mediated knockouts	Cellectis S.A.	U.S.A.	NCT04106076	9/23/2019
	TALEN	I	Programmed cell death 1 (PD-1) and CD52 knockout	Multiple myeloma	*Ex vivo*	CS-1-CAR modified T cells with CAR delivered by lentivirus and TALEN-mediated knockouts	Cellectis S.A.	U.S.A.	NCT04142619	10/29/2019
	TALEN	I	Programmed cell death 1 (PD-1) and CD52 knockout	CD22^+^ B cell acute lymphoblastic leukemia	*Ex vivo*	CD22-CAR modified T cells with CAR delivered by lentivirus and TALEN-mediated knockouts	Cellectis S.A.	U.S.A.	NCT04150497	11/4/2019
	Cas9	I/II	βTCRα, TCRβ, β-2 microglobin (B2M) knockout	B-cell leukemia	*Ex vivo*	CD19-CAR modified T cells with CAR delivered by lentivirus and Cas9 knockout B2M and TCR to create universal T cells	Chinese PLA General Hospital	China	NCT03166878	5/25/2017
	Cas9	I	TCRα, TCRβ, PD-1 knockout	Various malignancies	*Ex vivo*	Modified T cells with Cas9-mediated deletions and lentiviral transduction of NY-ESO-1 targeted TCR	University of Pennsylvania	U.S.A.	NCT03399448	1/16/2018
	Cas9	I/II	Disruption of the erythroid enhancer to *BCL11A* gene	β-thalassemia	*Ex vivo*	*Ex vivo* modified hematopoietic stem cells	CRISPR Therapeutics	U.K., Germany	NCT03655678	8/31/2018
	Cas9	I/II	Disruption of the erythroid enhancer to *BCL11A* gene	Sickle cell anemia	*Ex vivo*	*Ex vivo* modified hematopoietic stem cells	Vertex Pharmaceuticals Incorporated and CRISPR Therapeutics	U.S.A.	NCT03745287	11/19/2018
	Cas9	I/II	Creation of a CD19-directed T cell	Refractory B-cell malignancies	*Ex vivo*	CD19-directed T-cell immunotherapy	CRISPR Therapeutics	U.S.A., Australia	NCT04035434	7/29/2019
	Cas9	I	disruption of HPK1	refractory B cell malignancies	*Ex vivo*	CD19-CAR modified T cells with CAR delivered by lentivirus and Cas9 knockout of HPK1	Xijing Hospital	China	NCT04037566	7/30/2019
plasmid delivery	ZFN	I	E7 oncogene of HPV16 and HPV18 deletion	HPV-related malignancy	*In vivo*	Vaginal suppository with polymer to facilitate delivery	Huazhong University of Science and Technology	China	NCT02800369	6/15/2016
	TALEN	I	E6 and E7 oncogene of HPV16 and HPV18 deletion	HPV-related malignancy	*in vivo*	plasmid in a gel containing a polymer to facilitate delivery	First Affiliated Hospital, Sun Yat-Sen University	China	NCT03057912	2/20/2017
	TALEN	I	E6 and E7 oncogene of HPV16 and HPV18 deletion	HPV-related malignancy	*In vivo*	Plasmid in vaginal suppository with polymer to facilitate delivery	Huazhong University of Science and Technology	China	NCT03226470	7/21/2017
	Cas9	I	E6 and E7 oncogene of HPV16 and HPV18 deletion	HPV-related malignancy	*In vivo*	Plasmid in a gel containing a polymer to facilitate delivery	First Affiliated Hospital, Sun Yat-Sen University	China	NCT03057912	2/20/2017
Undefined, likely electroporation	Cas9	I	Programmed cell death protein 1 (PD-1) knockout	Metastatic non-small cell lung cancer	*Ex vivo*	Modified T cells	Peking University	China	NCT02793856	6/8/2016
	Cas9	I	Programmed cell death protein 1 (PD-1) knockout	Stage IV bladder cancer	*Ex vivo*	Modified T cells	Peking University	China	NCT02863913	8/11/2016
	Cas9	I	Programmed cell death protein 1 (PD-1) knockout	Metastatic renal cell carcinoma	*Ex vivo*	Modified T cells	Peking University	China	NCT02867332	8/15/2016
	Cas9	I	Programmed cell death protein 1 (PD-1) knockout	Hormone refractory prostate cancer	*Ex vivo*	Modified T cells	Peking University	China	NCT02867345	8/15/2016
	Cas9	I/II	Programmed cell death protein 1 (PD-1) knockout	EBV-positive, advanced stage malignancies	*Ex vivo*	Modified T cells selected for those targeting EBV positive cells	The Affiliated Nanjing Drum Tower Hospital of Nanjing University Medical School	China	NCT03044743	2/7/2017
	Cas9	II	Programmed cell death protein 1 (PD-1) knockout	esophageal cancer	*Ex vivo*	Modified T cells	Hangzhou Cancer Center	China	NCT03081715	3/16/2017
	Cas9	n/a	CCR5 knockout	HIV	*Ex vivo*	Modified CD34^+^ hematopoietic stem cells	Affiliated Hospital to Academy of Military Medical Sciences	China	NCT03164135	5/23/2017
	Cas9	I/II	Cas9-mediated creation of CD19 and CD20 or CD19 and CD22 CAR-T cells	Leukemia	*Ex vivo*	CAR T cells to CD19 and CD20 or CD19 and CD22	Chinese PLA General Hospital	China	NCT03398967	1/16/2018
	Cas9	I/II	Cytokine-induced SH2 protein (CISH) knockout	Metastatic gastrointestinal epithelial cancer	*Ex vivo*	Modified tumor-infiltrating lymphocytes	National Cancer Institute	U.S.A.	NCT03538613	5/28/2018
	Cas9	I	Programmed cell death protein 1 (PD-1) and TCR knockout	Mesothelin positive solid tumors	*Ex vivo*	CAR T cells to mesothelin with added PD-1 and TCR knockout	Chinese PLA General Hospital	China	NCT03545815	6/4/2018
	Cas9	I	CD7 knockout in CD7 CAR T cells	T-cell malignancies	*Ex vivo*	CAR T cells to CD7 and knockout of native CD7 to prevent self targeting	Baylor College of Medicine	U.S.A.	NCT03690011	10/1/2018
	Cas9	I	Correction of the hemoglobulin subunit β globulin gene	β-thalassemia	*Ex vivo*	*Ex vivo* modified hematopoietic stem cells	Allife Medical Science and Technology Co., Ltd.	Not specified	NCT03728322	11/2/2018
	Cas9	I	Programmed cell death protein 1 (PD-1) knockout	Mesothelin positive solid tumors	*Ex vivo*	CAR T cells to mesothelin with PD-1 knockout	Chinese PLA General Hospital	China	NCT03747965	11/20/2018
	Cas9	I/II	Cytokine-induced SH2 protein (CISH) knockout	Metastatic gastrointestinal epithelial cancer	*Ex vivo*	Modified tumor infiltrating lymphocytes	Masonic Cancer Center, University of Minnesota	U.S.A.	NCT04089891	9/13/2019
Long-term clinical follow-up post-intervention	ZFN	Follow-up	CCR5 knockout	HIV	*Ex vivo*	Modified CD4^+^ T cells, 12-year follow-up study	Sangamo Biosciences	U.S.A.	NCT04201782	12/17/2019
	TALEN	Follow-up	TCRα, TCRβ, CD52 knockout	Advanced lymphoid malignancy	*Ex vivo*	CD19-CAR modified T cells with CAR delivered by lentivirus and TALEN knockout CD52 and TCR to create universal T cells, 15-year follow-up study	Institut de Recherches Internationales Servier	U.K., Belgium, France, U.S.A.	NCT02735083	4/12/2016
	Cas9	Follow-up	Disruption of the erythroid enhancer to *BCL11A* gene	β-thalassemia and severe sickle cell anemia	*Ex vivo*	*Ex vivo-* modified hematopoietic stem cells, 15-year follow-up study	Vertex Pharmaceuticals Incorporated and CRISPR Therapeutics	U.S.A., U.K., Germany	NCT04208529	12/23/2019

U.S. clinical trials data base (clinicaltrials.gov) was accessed on 1/1/2020, trials not including interventions using gene editors were excluded. Abbreviations: CAR, chimeric antigen receptor; TCR, T-cell receptor.

**Figure 2:**
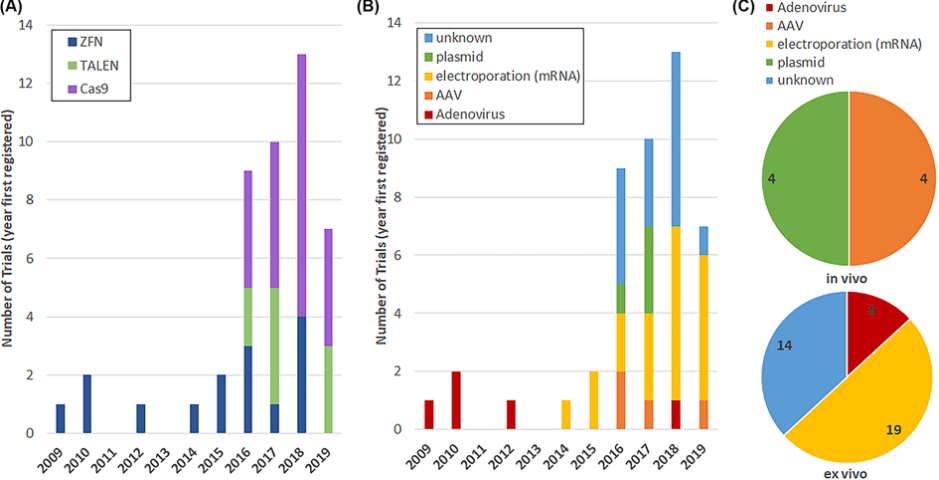
Trends in genome editor use in clinical trials Genome editing trials registered in the U.S. clinical trials database by year and selected genome editor (**A**), or delivery method (**B**) and delivery method grouped by *in vivo* or *ex vivo* use (**C**). Some trials did not have a clear delivery methodology (labeled as unknown). These unknown delivery methods are all *ex vivo* delivery making electroporation the most likely method. Data were accessed 1/01/2020.

Examination of the clinical trials data has shown a rapid increase in registered trials using genome editors, as well as a change in the selected genome editors in the recent years (Figure 2A). The first registered trial is from 2009, and between 2009 and 2015 less than or equal to two trials were registered per year, all of which focused on ZFNs. However, in 2016 and 2017 the number of trials jumped to 10 per year, with a further increase to 13 trials in 2018; 2016 was also the first year TALENs and Cas9 trials were registered, reflecting a change in the genome editing agent as well as number of trials. Separating the data by delivery method also reveals change over time in the selected method (Figure 2B). For example, early trials exclusively used adenovirus-based delivery, whereas later trials also used adeno-associated virus (AAV), polymer-mediated plasmid delivery, and electroporation. Choice of *in vivo* versus *ex vivo* delivery also changed over time (Figure 2C)*. Ex vivo* delivery, where the cells are altered in the lab and then infused into the patient, primarily utilizes electroporation as the methodology, but transduction by adenovirus is also used. *In vivo* delivery has focused on both localized delivery and systemic delivery. Registered clinical trials with localized delivery prior to 2019 were focused on polymer-mediated plasmid delivery of ZFNs, TALENs, or Cas9 to the vagina. However, localized delivery sites are expanding with a new trial in 2019 proposing direct injection into the eye of Cas9 via AAV vector. To date, systemic delivery has been limited to IV-based delivery of AAV using ZFN-mediated addition of a gene to hepatocytes.

### *Ex vivo* cell alteration and infusion trials

Current clinical trials using *ex vivo* alteration by genome editors overwhelmingly focus on the modification of T cells to disrupt gene expression to either treat HIV-1 infection, or to enhance the efficacy of engineered T cells to counter cancer. The earliest gene editing studies have been focused on disruption of the gene encoding CCR5, a major co-receptor that facilities HIV-1 entry into T cells. This idea of gene editing CCR5 for curing or controlling HIV infection in patients was bolstered by the ‘Berlin Patient’, an HIV-positive patient who was treated for acute myeloid leukemia by bone marrow transplant in 2007. The physicians decided to perform the transplant with bone marrow from a donor who was homozygous for a CCR5 mutation known as CCR5 Δ32. This mutation results in loss of CCR5 expression on the cell surface, and, in this case of the Berlin Patient, resulted in a clinical cure of HIV infection [18,19]. CCR5 was chosen as an early target for gene editing because it has naturally occurring mutations that lead to loss of surface expression without any known severe adverse effects, and the effective loss of CCR5 was demonstrated to cure the patient of HIV. While the Berlin Patient underwent allogenic bone marrow transplant, in patients without leukemia it may be possible to disrupt the *CCR5* gene *ex vivo* and then return the engineered cells to the patients via autologous stem cell transplant. ZFNs to knockout CCR5 in human cells were already under development when the Berlin Patient was treated, and the first clinical trial was initiated in 2009 [20,21]. Additionally, although the majority of HIV therapy trials have focused on ZFNs, CRISPR/Cas9 has also been shown experimentally to provide a functional knockout of CCR5 which conferred resistance to HIV infection [22,23], and the first human clinical trial of Cas9 for CCR5 knockout was registered in 2017.

The use of CRISPR Cas9 in HIV also opens several new possibilities for HIV therapy. While The Berlin Patient was cured of HIV through transplantation with CCR5 Δ32 bone marrow, several other attempts to perform this type of cure have failed and other patients have had viral rebound [24,25]. One possible reason for viral relapse is that there are two main co-receptors used for HIV entry, CCR5 and CXCR4. While most new infections are caused by a CCR5 utilizing variant of HIV, many chronic patients have both CCR5 and CXCR4 utilizing variants [24]. In pre-clinical studies, ZFNs have been developed for CXCR4 and have demonstrated utility together with CCR5-targeting ZFNs [26]. Pre-clinical studies of CRISPR/Cas9-based strategies have also been used to successfully knock out both CCR5 and CXCR4, and this method can be more efficient than ZFNs as only multiple gRNAs, rather than multiple proteins, need to be delivered [27,28]. Cas9 also presents the potential to eradicate HIV-1 reservoirs through the disruption of integrated HIV-1 proviral DNA in a variety of cell types [25]. One *in vivo* study demonstrated that AAV delivery of Cas9 and gRNAs resulted in deletion of a large section of the genome-integrated HIV-1 provirus in a variety of tissues [29]. And finally, recent work demonstrated that a combination of gene editing, using AAV-delivered CRISPR components, with long-acting slow-effective release antiretroviral therapies (LASER ARTs) could clear latent infectious reservoirs of HIV-1 in humanized mice [30]. While CXCR4 targeting, dual targeting of CCR5 and CXCR4 and deletion of HIV provirus have not yet reached the clinical stage, the pre-clinical studies highlight where genome editors may take HIV-1 therapy in the future.

**Figure 3:**
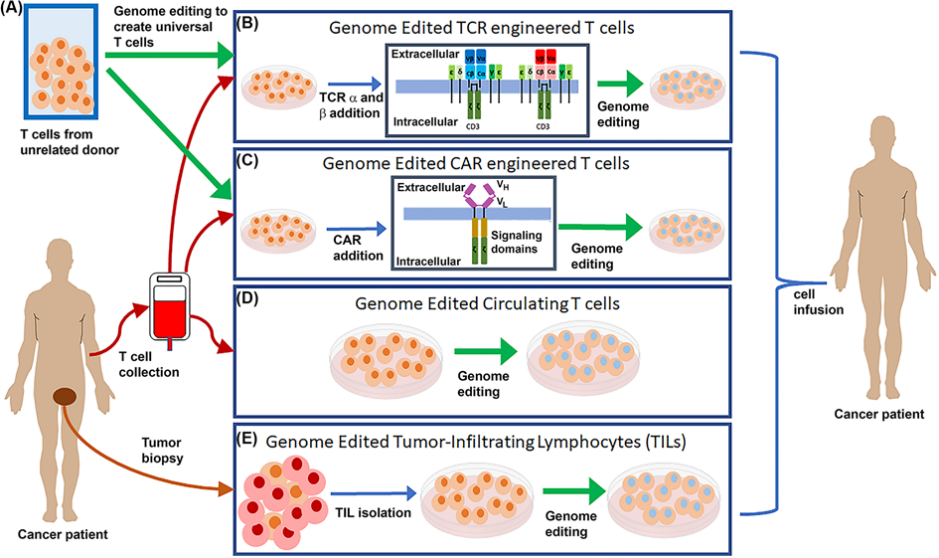
Genome editing used to enhance ACT of T cells for cancer therapy (**A**) Genome editing (highlighted by green arrows) is being explored to create universal donor T cells to serve as the basis for TCR and CAR T-cell engineering. Genome editing is also being explored to enhance the survival and/or efficacy or prevent self-targeting of both natural (circulating T cells and TILs) and engineered (TCR and CAR) T cells. (**B**) TCR engineered T cells have the addition of a second set of TCR α and TCR β genes (highlighted in red and pink) which are present in addition to the naturally occurring TCR α and TCR β genes (highlighted in blue). (**C**) CAR engineered T cells have a chimeric cell receptor with an scFv composed of variable heavy and light chains (V_H_ and V_L_) of an antibody as the extracellular portion fused to intracellular T-cell signaling domains to cause T-cell activation upon interaction with the targeted cell surface marker. Genome editing is also being applied to circulating T cells collected from a patient’s blood (**D**) and to isolated tumor infiltrating lymphocytes (**E**), which utilize the native T-cell targeting to destroy tumor cells. Abbreviation: scFv, single-chain variable fragment.

While HIV-1 therapy was the earliest gene editing clinical trial and remains a major focus, many of the recent trials have focused on tailoring T cells for adoptive cell transfer (ACT) for cancer. There are four types of ACT to which gene editing is being applied: general T cells, tumor-infiltrating lymphocytes (TILs), T-cell receptor (TCR) bearing cells, and chimeric antigen receptor (CAR) bearing T cells (Figure 3). The first two types of ACT to which gene editing is being applied, general T cells and TILs, involves the natural, unaltered, T-cell targeting with gene editing to knock out a gene of interest. The second two ACT types, TCR and CAR modified T cells, involve genetically altering T cells via gene transfer in order to direct them to target cells of interest, and then further altered these cells by gene editing. Gene editing is being applied to ACT in several ways: to enhance survival after transfer, increase efficacy, prevent self-targeting, or develop universal T cells.

The earliest clinical trial utilizing CRISPR (NCT02793856) focused on collecting general T cells from peripheral blood and utilizing Cas9 to knock out the programmed cell death protein 1 (*PD-1*) gene. PD-1 is a protein found on the surface of T cells that negatively regulates T-cell activation upon interaction with its ligand, PD-L1. High expression of PD-1 on the T-cell surface accelerates T-cell tolerance and exhaustion, limiting the efficacy of T cells against tumors. The primary safety concern with the first CRISPR trial was that use of general, non-specific T cells, rather than TILs collected directly from the patient’s tumors, might lead to a general overactivation of the patient’s immune system [31]. While the concern is valid, and toxicity due to anti-PD-1 and anti-PD-L1 therapies is a known issue [32], systemic therapy with PD-1 and PD-L1 blockers has been approved by the U.S. Food and Drug Administration (FDA) for a variety of cancers [33], which the researchers felt mitigated the concern [31]. The data from this first in human CRISPR trial reported thus far indicate no major adverse events; the trial was not designed to examine efficacy, however, so the effectiveness of this therapy is currently unknown [34,35].

One major limitation to the first CRISPR clinical study is that the collected T cells are not enriched for cells likely to respond to the tumor cells. One method around this is the use of TILs collected directly from tumor resections. TIL therapy has a long history of clinical trials in melanoma [36], including long-term robust patient response, and is being developed for other cancers including cervical, ovarian, kidney, gastrointestinal, and head and neck cancers [37]. Two TIL clinical trials to treat gastrointestinal cancer involve the collection of TILs from patient tumors and enhancing their antitumor efficiency through knock out of the gene *Cish*. TILs capable of targeting gastrointestinal cancer cells have been found in patients [38]; however, Cish, a member of the suppressor of cytokine signaling (SOCS) family, has been shown to block the avidity of the TILs and reduce their activity against cancer [39]. In a mouse model, knockout of the *Cish* gene in TILs led to increased expansion and responsiveness, leading to tumor regression [39]. Furthermore, pre-clinical studies of ZFN-mediated knockout of PD-1 in TILs for treatment of melanoma suggest that clinical trials using TILs with gene knockouts will be pursued in the future [40].

While the trials on TILs and general T-cell gene editing focused on modifying naturally targeted T cells, the majority of *ex vivo* gene editing trials focus on using gene editors to enhance TCR and CAR-engineered T cells (Figure 3A–C). Engineering the T-cell recognition through either the addition of a specific TCR or CAR to T cells circumvents several of the issues presented with TILs: first, not all patients have tumor-responsive T cells; second, these cells must be collected from surgical resections of tumors; finally, there is no control over the targeted cancer antigen. TCR-engineered T cells are based on transferring the genes encoding the α and β chains of a TCR specific to an antigen unique to, or overexpressed in, tumor cells into collected T cells that are then re-infused into the patient. In contrast, CARs are artificial chimeric receptors created to target cell surface markers on cancer cells. CARs are artificial receptors consisting of an external single-chain variable fragment (scFv) created from the variable regions of the heavy and light chains of antibodies for target specificity and internal T-cell signaling domains leading to T-cell activation upon scFv binding [41,42]. Similar to TCR engineering, CAR can be transferred in to collected T cells and then re-infused into the patient. TCR and CAR engineered T cells each present unique advantages and challenges that gene editing can address [43]. TCR engineered T cells have some advantages over CAR engineered T cells: the TCR is able to recognize intracellular proteins, including novel tumor antigens created by mutations, whereas CAR recognizes only proteins present on the cell surface [43]. However, the TCR recognizes peptides from intracellular proteins presented on the surface of the tumor cell in the major histocompatibility complex (MHC) class I molecules, and loss or down-regulation of MHC molecules is a major source of tumor escape from immune surveillance which could limit the efficacy of TCR-based therapies [44]. CAR T-cell recognition of target is independent of MHC expression, but limited to cell surface markers, which are commonly present on both diseased and normal cells, which can limit the utility of CAR T-cell therapy [43]. Both TCR and CAR T-based therapy has been pursued independent of gene editing, but gene editing is opening new possibilities for enhancing immune therapy and will be discussed in more detail.

A number of TCRs that mediate T-cell targeting of tumor cells have been identified and tested in clinical trials [45]. One of the most promising of these TCRs is NY-ESO-1 [46]. T cells engineered to express the NY-ESO-1 TCR have been used as effective therapy for melanoma, synovial cell carcinoma, and myeloma [45]. One potential limitation to TCR therapy is the presence of the endogenous TCR α and β chains, which could pair with the engineered TCR α and β chains to create heterologous pairs. These heterologous pairs might reduce efficacy due to their inability to recognize the intended target, and cause toxicity due to recognition of unintended targets [47]. The one registered gene editing trial to date (NCT03399448) which focuses on engineered TCRs uses CRISPR/Cas9 to knockout the endogenous TCR α and β chains (to remove the potential for heterologous TCR pairings) as well as PD-1 (to enhance TCR engineered T-cell efficacy).

The majority of *ex vivo* gene editing trials focus on the enhancement of CAR engineered T-cell therapy. CAR T-cell therapy is the first form of gene transfer therapy to gain FDA approval, with two approved therapeutics, Tisagenlecleucel (Kymriah) and axicabtagene ciloleucel (Yescarta or Axi-Cel) for CD19-positive refractory pre-B cell acute lymphoblastic leukemia and diffuse large B-cell lymphoma, respectively [43,48]. While both approved therapeutics target B-cell malignancies, CAR T therapy represents an extremely large clinical trial space, with over 250 clinical trials in a wide variety of cancers currently underway [43,49]. Of these clinical trials, a number of them include gene editors to further enhance the CAR engineered T cells. Delivery of the CAR gene into T cells is mediated by γ retroviral or lentiviral vectors; the advantage of these delivery systems is a high rate of gene transfer and stable expression of the CAR. However, these viral vectors integrate the CAR randomly into the genome, which can lead to varied CAR expression, insertional mutagenesis, overexpression of adjacent genes, and disruption of genes at the site of integration. While evidence of these issues is yet to been seen in CAR therapy [50], insertional oncogenesis and overexpression of adjacent genes have been seen in a human gene therapy trials using these viral vectors [51–55]. One potential method around this is to direct where the CAR is inserted into the genome. Disruption of the TCR α constant (TRAC) locus has been demonstrated using TALENs, Cas9, and megaTAL Nucleases, with both TALENs and Cas9 being highly effective at disrupting TRAC expression and thereby reducing TCR expression on the T-cell surface [56]. Recently, this ability has been expanded to allow specific insertion of the CAR into the TRAC locus using Cas9 or TALENs [57–59]. Integration of the CAR into the TRAC locus increased CAR T cell anti-tumor activity in mice models. The insertion of the CAR into the TRAC locus has the advantage of also disrupting the native TCR surface expression, which reduces the likelihood of graft-versus-host disease (GvHD) and increases the safety of allogenic CAR T therapy [60]. This idea forms the basis for the clinical trial NCT03398967, which proposes to integrate two CARs (to CD19 and either CD20 or CD22) into the TRAC locus for allogenic ACT. The created cells will lack native TCR expression, which will facilitate allogenic ACT; and the dual targeting is hoped to prevent the relapse seen in CD19 CAR T therapy, where the cells lose CD19 expression by utilizing two surface targets [48,61].

A number of clinical trials are focused on the creation of universal CAR T cells, through knockout of the TCR through removal of TRAC alone or of the TRAC and TCR β constant (TRBC) loci. Removal of the TCR should prevent the transferred T cells from recognizing host antigens, which leads to GvHD. However, the presence of human leukocyte antigen (HLA) system proteins on the surface of the transplanted T cells, if mismatched with the host, can lead to rapid rejection of the transplanted cells. Some of the studies aim to both prevent GvHD and rapid rejection. For example, the removal of β-2 microglobulin (B2M), which is necessary for HLA expression, in addition to TRAC and TRBC resulting in increased efficacy of CD19 CAR T cells compared with removal of the TCR alone in mice models [62,63]. The combined knockout of the TCR with B2M by Cas9 in CD19 CAR T therapy is currently being tested in clinical trial (NCT03166878).

Another gene commonly targeted for removal by gene editing agents in CAR therapy for leukemia is *CD52*. CD52 is the protein targeted by alemtuzumab, a chemotherapeutic agent commonly used in leukemia treatment, but which can also kill the transplanted CAR T cells. Removal of both the TCR (to prevent GvHD) and CD52 using TALENs allowed generation of allogenic CD19 CAR T cells that could be used in combination with alemtuzumab in a mouse model [64]. The utility of these TALEN edited CD19 CAR T cells is the basis of three currently running clinical trials (NCT02735083, NCT02746952, and NCT02808442). Prior to initiation of these trials, two infants with relapsed refractory CD19^+^ B-cell acute lymphoblastic leukemia were treated with TALEN-mediated CD52 and TCR knockout CD19 CAR T cells in combination with anti-CD52 therapy [58]. Both infants achieved molecular remission and had lasting CD19 CAR T-cell persistence. In addition to removing antigens targeted by chemotherapy agents, the ability to remove genes allows application of CAR T-cell therapy to T-cell malignancies through the removal of the targeted T-cell surface marker. CD7 is highly expressed on a variety of T-cell malignancies, but expression of the CD7 CAR prevented CAR T-cell expansion due to fratricide in culture. CD7 knockout through Cas9 resulted in efficient expansion of CD7 CAR T cells while retaining CD7 selective killing in a mouse model [65], and is the basis of a current clinical trial (NCT03690011).

Another common gene editing approach in CAR T cells is the knockout of PD-1, either alone or in combination with TCR knockout. Selective knockout of PD-1 in the therapeutic T cells may be advantageous over systemic anti-PD-1 therapy, which can have significant immune toxicities [66]. Combination of CAR therapy with PD-1/PD-L1 blockade is currently being examined in clinical trials, and preliminary results from CD19 targeted CAR T-cell therapy has shown limited toxicity and enhanced CAR T-cell survival [67–69]. The PD1 knockout by Cas9 has been shown to enhance the efficacy of CD19 and mesothelin CAR T cells in mouse models [70,71]. PD1 knockout has also been combined with the gene knockouts designed to create universal CAR T cells [62,63]. Clinical trials using CRISPR/Cas9 editing in CAR T cells are underway, including PD1 knockout in combination with mesothelin CAR T cells without or with TCR knockout (NCT03747965 and NCT03545815, respectively), and with CD123 CAR T cells with CD52 knockout (NCT03190278 and NCT03203369).

Another focus for *ex vivo* gene editing trials is the treatment of two hemoglobinopathies, β-thalassemia and sickle cell disease. While gene therapy has previously been successfully applied to β-thalassemia, through lentiviral transfer of the β-globin gene (HBB) [72], the concern remains that random integration might result in oncogenesis [73]. Gene editing provides an alternative to viral delivery of intact genes for the treatment of β-thalassemia and sickle cell anemia. While a number of studies have focused on utilizing genome editors to facilitate the correction of mutations in HBB, or to aid in site specific incorporation of an intact copy of HBB [74–78], others have focused on the editing ability alone. It has long been known that mutations resulting in persistence of fetal γ-globin expression (usually silenced at birth) reduce the debilitating effects of mutations in β-globin, including β-thalassemia and sickle cell disease [79]. Genome editing has been utilized to recreate the large deletion which causes Hereditary Persistence of Fetal Hemoglobin (HPFH), and hematopoietic stem cells that were Cas9-edited to create HPFH show increased expression of fetal γ-globin [80]. Other studies have focused on deleting the fetal globin repressor BCL11A, leading to increased fetal γ-globin expression. ZFN, TALEN, and Cas9 have all been applied to BCL11A knockout and ZFN-edited hematopoietic stem cells were able to engraft in mice and express fetal γ-globin [81,82]. Disruption of BCL11A in hematopoietic stem cells via ZFN or Cas9 followed by cell transplantation to treat β-thalassemia or sickle cell disease clinical trials are currently underway (NCT03432364, NCT03653247, NCT03655678, and NCT03745287).

### *In vivo* gene editing trials

Clinical trials of genome editors delivered *in vivo* have also been initiated. The trials to date have focused on easily accessible tissues, such as the cervix, eye, and liver. The latter being the most likely place for the delivered editing agent to accumulate. The largest set of *in vivo* genome editing trials has focused on eradication of integrated E6 and E7 HPV genes in cervical cancer. While vaccines are now available for HPV, the vaccines do not provide therapeutic effect for those who have already developed cervical cancer [83]. All of the trials to date have originated in China, starting with a trial in 2016 that proposed the use of ZFNs, but in 2017 both TALEN and Cas9 trials were also registered. These trials take advantage of the easy access to the cervix through the vagina, and in each case the delivery vehicle is a polymer gel suppository, the safety of which was recently tested in animal models [84]. Non-viral delivery of ZFNs, TALENs, or Cas9 targeting the integrated E7 oncogene reduced tumor burden in mouse models [85–87]. While the clinical trials to date have focused on non-viral delivery, AAV delivery of Cas9 targeted to E6 and E7 has also been shown effective in xenograft models [88,89]. Targeting E6 and E7 with genome editors often directly results in cell death; however, targeting these genes can also increase the sensitivity of tumors to other modes of therapy, including chemotherapy and radiotherapy [83], providing an increased rationale for utilizing these genome editors. One particularly interesting study examined HPV16^+^ patient xenografts of anal cancer in immunodeficient mice followed by Cas9 targeting of E6 and E7 [88]. This work demonstrated a significant reduction in tumor growth in mice [88], suggesting that the therapeutic utility of genome editors for HPV treatment extends beyond cervical cancer.

While the largest set of *in vivo* genome editing trials was focused on treatment of cervical cancer, early use of *in vivo* ZFN gene editors was aimed to treat hemophilia B by replacing disease causing mutations in the *F9* gene, which causes a deficiency of blood coagulation factor IX [90,91]. While the traditional gene editing approach is to replace the damaged gene at its native locus, if transcription from the native locus is too low there may not be a therapeutic effect. To address this issue, a subsequent study utilized AAV vectors to deliver a pair of ZFNs targeting gene replacement constructs at the albumin locus, which functions as a safe harbor locus with high transcriptional activity [92]. As the delivery of two targeted ZFNs and a cDNA requires three AAVs to hit the same cell after IV injection, utilizing a highly expressed ‘safe site’ in the genome is more likely to result in therapeutic levels of protein expression. A commonly used safe site for gene integration is the adeno-associated virus integration site 1 (AAVS1), which can also be targeted for gene integration by ZFNs [93,94].

Another commonly used integration site is the albumin locus, which is an especially attractive site for genome integration of secreted proteins. Exon 1 of the albumin gene encodes a secreted peptide that is cleaved from the final albumin product, so the addition of the cDNA with a splice acceptor site into intron 1 allows the creation of a new protein combining the secretory peptide and the protein of interest [92]. The initial work on this system showed the ability insert the cDNA and a number of proteins including Factor IX (hemophilia B), Factor VIII (hemophilia A), α-Galoactosidase A (Fabry Disease), α-l-Iduronidase (Hurler Syndrome, a.k.a. mucopolysaccharidosis type I (MPS I)), Iduronate-2 Sulfatase (Hunter Syndrome, a.k.a. mucopolysaccharidosis type II (MPS II)), and Acid β-Glucosidase (Gaucher Disease) [92]. Treatment of animal models of hemophilia A and B resulted in significant improvement in blood clotting, and intervention in MPS I and II prevented or reduced neurocognitive deficit in young animals [92,95,96]. Of these initial gene sets, replacement of Factor IX, α-l-Iduronidase, and Iduronate-2 sulfatase for hemophilia B, MPS I and II have advanced to clinical trials. The early stage results from the MPS II study suggested the treatments were safe, but also showed very low rate of editing events and inconclusive therapeutic efficacy in the low and medium dose patients [97]. Patient treatments are expected to continue through 2019, but future trials will likely focus on second-generation ZFNs that may have increased activity in human liver cells [97].

Beyond the use of ZFNs, the first AAV Cas9 trial was registered in 2019. The trial utilizes AAV to deliver Cas9 to the eye as a cure for Leber congenital amaurosis type 10 (LCA10). LCA10 is an ideal candidate for Cas9 therapy, as the most common LCA10 causing mutation occurs within an intron of the *CEP290* gene, creating a novel splice site which alters the mRNA to create a premature stop codon [98]. The treatment utilizes two sgRNAs which together mediate loss of part of the intron or inversion of this partial intron, either of which results in normal expression of CEP290 protein in patient cells. Furthermore, the eye is readily accessible, and subretinal injection in mice and primates resulted in sustained gene editing at a level expected to be therapeutic in humans [98].

### Clinical trials with inactive nucleases for transcriptional activation

Another interesting aspect is the use of ZFNs, TALENs, and Cas9 with inactive or absent nuclease function to allow semi-specific transcriptional activation or suppression. The advantage of this approach is that sequence specificity can be retained for targeted gene activation or inactivation without causing permanent alteration to the genome. From a clinical standpoint, this can reduce the concern about creating unwanted mutations as the DNA remains intact while still allowing alteration in gene expression. While the initial studies were limited to Zinc Finger Protein (ZFP)-based transcription factors for activation of VEGF expression (Table 2), these studies suggest a path forward for more recent transcriptional activators and epigenome editors, based on deactivated Cas9 (dCas9), ZFPs, and transcription activator-like effector proteins (TALEs), which can be used to not only modulate transcription temporarily but also alter genome structure through methylation and acetylation to allow permanent changes in gene transcription [11,99,100]. Later in this review we will discuss the recent technological advances regarding CRISPR-based control of gene expression and the epigenome in greater detail.

**Table 2:** Clinical interventions using inactive genome editors as transcription factors

Vector	Transcription factor type	Phase	Target Gene and effect	Disease	*Ex vivo/in vivo*	Intervention	Sponsor organization	Country	NCT number	Date posted
Naked plasmid	ZFP TF	I	VEGF-A increased expression	Artheriosclerosis and intermittent claudication (lower limb ischemia)	*In vivo*	Injection into the leg	National Heart, Lung, and Blood Institute (NHLBI)	U.S.A.	NCT00080392	3/30/2004
	ZFP TF	I	VEGF increased expression	Diabetic limb neuropathy	*In vivo*	Injection into the leg	Sangamo Therapeutics	U.S.A.	NCT00110500	5/10/2005
	ZFP TF	II	VEGF increased expression	Diabetes type 1 and 2, diabetic limb neuropathy	*In vivo*	Injection into the leg	Sangamo Therapeutics	U.S.A.	NCT00406458	12/4/2006
	ZFP TF	II	VEGF increased expression	Diabetes type 1 and 2, diabetic limb neuropathy	*In vivo*	Injection into the leg	Sangamo Therapeutics	U.S.A.	NCT00476931	5/22/2007
	ZFP TF	II	VEGF increased expression	Diabetes type 1 and 2, diabetic limb neuropathy	*In vivo*	Injection into the leg	Sangamo Therapeutics	U.S.A.	NCT00665145	4/23/2008
	ZFP TF	II	VEGF increased expression	Amyotrophic lateral sclerosis	*In vivo*	Injection into neck, arm, or leg	Sangamo Therapeutics	U.S.A.	NCT00748501	9/8/2008
	ZFP TF	II	VEGF increased expression	Diabetes type 1 and 2, diabetic limb neuropathy	*In vivo*	Injection into the leg	Sangamo Therapeutics	U.S.A.	NCT01079325	3/3/2010

U.S. clinical trials data base (clinicaltrials.gov) was accessed on 1/1/2020.

### Looking forward: upcoming areas for gene editor clinical trials

The genome editing landscape is moving very quickly and a number of potential therapies beyond those currently in clinical trials are rapidly approaching the clinical trial space. Many pharmaceutical companies put out regular updates on their development pipelines, giving insight into the likely coming trials. The majority of the anticipated *ex vivo* trials revolves around CAR T therapy and hemoglobinopathies (β-thalassemia and sickle cell disease) [101]. Beyond these anticipated trials, another likely area of *ex vivo* clinical genome editor use are the monogenic primary immunodeficiencies (PIDs). Similar to the *in vivo* ZFN trials, treatment of the PIDs would require the insertion of a correct gene (or cDNA) copy. However, treatment of these PIDs is in some way ideal for genome editing, as the standard treatment is typically hematopoietic stem cell transplant, meaning the cells can be modified *ex vivo* which removes the *in vivo* delivery problem encountered with *in vivo* AAV trials [97]. Furthermore, these diseases are severe enough that they are already the subject of gene therapy trials [102]. Pre-clinical studies using genome editors and donor DNA have been demonstrated for three PIDs; X-linked severe combined immunodeficiency (SCID-X1) [103–105], chronic granulomatous disease (CGD) [106–111], and Wiskott–Aldrich syndrome (WAS) [112]. Pre-clinical studies in SCID-X1 have focused on addition of a functional copy of interleukin-2 receptor subunit γ (IL2RG) into the AAV1 safe harbor site or, more commonly, into native gene site [103–105]. These studies were able to demonstrate functional gene expression and long-term engraftment in immunodeficient mouse models utilizing ZFNs [103,105] and Cas9 [104] genome editing followed by integration of the provided therapeutic gene copy in SCID-X1 patient cells. Similar to the studies in SCID-X1, genome editing followed by integration of provided sequence to insert functional gene copy has been demonstrated in CGD patient cells and resulted in long-term engraftment in immunodeficient mice using TALENs [108], ZFNs [107,108,110], and Cas9 [106,109], laying the groundwork for clinical trials in this space.

Beyond *ex vivo* trials, there are some promising advances in *in vivo* treatment of readily accessible tissues, such as the eye or ear. The eye is an attractive site for genome editing as direct access and self-contained nature of the eye reduces systemic effects. AAVs for delivery to the retina have already been identified and tested clinically, with one AAV-based gene therapy already approved and tissue specific promoters are also known which can further limit expression to the cells of interest [113,114]. The first Cas9 clinical trial for blindness, treatment of LCA10, has already been registered (NCT03872479) and was discussed in the current trials section. Also under development is Cas9 therapy involving the knockout of the endogenous gene copies and replacement with a functional copy for autosomal dominant cone–rod dystrophy (CORD6)-mediated blindness which has progressed to use in non-human primates [115]. Beyond the initiated LCA10 trial and CORD6 experiments, CRISPR-based therapeutics are being developed for a range of other inherited retinal disorders and multifactorial retinal diseases which has been reviewed elsewhere [116,117]. In addition to the eye, the ear is another site that also allows ready access for genome editing. While the studies in the ear have not progressed as far as the eye, correction of the *USH2A* gene responsible for both visual and hearing impairment in Usher syndrome has been shown in patient cells utilizing Cas9 and an DNA template [118]. Beyond *in vitro* cell manipulation, Cas9 nickases with paired sgRNAs in combination with an oligonucleotide donor template editing of zygotes were able to prevent age-related hearing loss in an mouse model [119]. Finally, Cas9 was used to selectively disrupt the mutant allele of Tmc1 (transmembrane channel-like gene family 1) associated with autosomal dominant hearing loss while sparing the wild-type allele and preventing progressive hearing loss [120,121].

In addition to easily accessible sites, liver targeted therapeutics are under development due to accumulation in liver of intravenously injected materials [101]. Several groups have published on the potential to treat α-1 Antitrypsin Deficiency (AATD) utilizing Cas9. α-1 antitrypsin (AAT) is secreted by the liver, and if the mutant allele is present, the protein aggregates in hepatocytes causing liver fibrosis, cirrhosis, and cancer and the reduction in circulating AAT also results in emphysema and Chronic Obstructive Pulmonary Disease (COPD) in the lungs. Cas9 has been used to knockout the mutant *AAT* gene or to knockin the normal gene in to a safe harbor site in mouse models [122–124]. Research has shown that dual delivery of both Cas9 targeted to ATT in combination with a donor template will knockout the mutant allele and integrate the wild-type allele, leading to expression of the wild-type AAT in mouse models [124,125]. In addition to therapeutics development to treat AATD in liver, non-viral, lipid nanoparticle (LNP) delivery of Cas9 facilitating hepatocyte selective targeting for the editing of mouse liver transthyretin gene, the mouse homolog of the human gene associated with Transthyretin Amyloidosis, which is caused by a buildup of misfolded transthyretin protein [126]. The *in vivo* delivery resulted in a greater than 97% reduction in serum and liver protein levels and the reduction persisted for 12 months [126]. The authors of this paper, also state this methodology can be applied to Hepatitis B treatment [101]. Hepatitis B treatment by Cas9 and other genome editors, as well as treatment for other oncolytic viruses, has been reviewed extensively elsewhere [127–129].

While the previously discussed potential disease targets focus on diseases in tissues already under trial for genome editors, there are also development pipelines for new tissues and diseases, including Cystic Fibrosis (CF), Duchenne muscular dystrophy (DMD) [101], and spinal cord injuries [130]. CF was the target of the earliest gene therapy trials as well as many ongoing gene therapy trials, one reason for this is the lungs can be readily targeted by inhalation [131,132]. However, to date, the efficacy has been too low in these trials for therapeutic effect [131,132]. Genome editors present a new possibility for therapeutic treatment of CF, although the low efficacy with gene therapy delivery agents studied to date may also apply to the delivery of genome editors. Repair of CF mutations has been demonstrated with ZFNs and Cas9 *in vitro* as well as in patient induced pluripotent stem cells [133–140], although testing in animal models has not yet been performed. CF is an interesting and challenging disease as the CF transmembrane conductance regulator (CTFR) gene is very large and there are numerous mutations associated with the disease. Several gene editing strategies have been used to treat CF including Cas9 targeted removal of mutations that lead to nonfunctional protein splicing [135], ZFNs and Cas9 used to repair the ΔF508 mutation [134,137–140], ZFNs used for the targeted addition of exons 11–27 of the CTFR gene into exon 11 as a functional gene correction [136], and Cas9 utilized to add a functional copy of the CTFR gene into an AAVS1 safe site [133].

Similar to CF, the gene encoding dystrophin, called DMD, is very large and there are numerous documented mutations along the entire gene that can cause DMD. The *DMD* gene is an interesting target for genome editing because a wide range of edit types can act as a functional cure including exon deletion, exon skipping, exon reframing, and exon knockin, depending on the specific disease causing mutation present [141]. While the use of Cas9 for DMD has been reviewed elsewhere [141], there are a few studies in this field worth highlighting specifically. The first is the use of Cas9 to correct the ΔE50-MD canine model of DMD, the present study examined both localized muscle delivery and systemic delivery via AAV. Systemic delivery resulted in expression of dystrophin in multiple muscles, including cardiac muscle at rates near normal at 8 weeks post-systemic delivery [142]. While longer term studies are needed in larger animal models, such as canines, a long-term study was recently completed in a mouse model of DMD. The present study utilized systemic AAV delivery of Cas9 in neonatal mice and found a single dose administration resulted in sustained editing and dystrophin protein expression 1 year after treatment [143].

### Germline editing and CRISPR babies

The rapidly advancing CRISPR toolkit holds many promising developments toward advancement of clinical medicine, although there are several safety and ethical concerns still to be addressed. One of the areas of greatest concern is germline editing, which creates a heritable alteration in the genome [144,145]. While an in-depth discussion of the ethical concerns and governmental regulatory concerns raised by germline editing are beyond the scope of this review, the actions in this area have the potential for far reaching effects in clinical genome editing and public image of these technologies [145]. We have therefore provided a brief overview of the technology as used in research and in humans [146].

Cas9 editing of zygotes has been applied to DMD treatment in a mouse model, creating a permanent and potentially inheritable change in the genome [147]. The ability to edit zygotes provides a pathway for eliminating fatal or debilitating monogenetic diseases and could act as a compliment to preimplantation genetic diagnosis [148,149]. This research has gone beyond animal models and editing of human zygotes has also been performed utilizing both non-viable and potentially viable embryos [146,150–153]. While the researchers publishing these embryo studies highlighted areas of concern as well as promise, such as the need for increased fidelity, specificity, and reproducibility, more recently, CRISPR edited embryos were implanted and resulted in the birth of twins in China [149,154]. While it remains unclear if and exactly how the babies’ genes were modified, there has been a flurry of debate regarding germline editing [149,154,155]. This event has created a push for a moratorium on editing human embryos and the World Health Organization announced the establishment of a committee to devise guidelines for Human Gene Editing [149].

## Issues limiting *in vivo* clinical use of CRISPR

While clinical trials have already begun, both *ex vivo* and *in vivo* in humans, there are still some issues that may limit the clinical use of CRISPR *in vivo.* The limiting issue which has received the most attention is the delivery problem: how do we accurately deliver CRISPR, control its activity, and limit off-target events [156]? For example, while AAV delivery is advantageous for gene therapy as it provides the possibility of long-term expression, this may lead to undesirable effects as the CRISPR enzyme would be expressed indefinitely. Non-viral delivery may allow temporal control of CRISPR activity, but the efficacy has traditionally been lower than viral delivery, although recent work with LNPs shows promise for highly efficient non-viral vectors [126]. Due to the diversity of delivery options; CRISPR Cas9 can be delivered as DNA, RNA, or ribonucleoprotein (RNP), a wide variety of delivery vehicles and methods have been developed and several reviews have focused on the methods to achieve delivery with both viral and non-viral vectors [17,157]. Beyond delivery to cells in general, the specificity of delivery is also important as in some cases a specific organ or cell type must be targeted. To date, most *in vivo* clinical trials have focused target tissues with direct access, such as the cervix or eye, or alterations in liver. As the field expands to more disease targets, the ability to target the therapy, either through controlled, tissue-specific expression of Cas9, or cell-specific targeting will become more important. Other limitations to *in vivo* delivery, which have received less attention that the delivery issue, are the detection and consequences of off-target effects, and immunogenicity of CRISPR therapies.

### CRISPR immunogenicity

**Figure 4:**
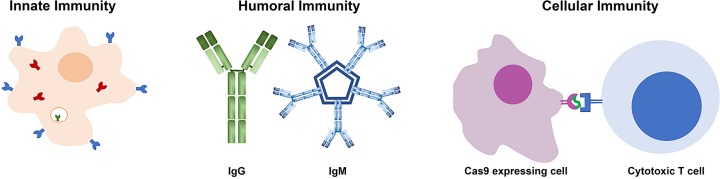
Mechanisms of Cas9 immunity seen in experimental studies Innate immunity is mediated by pattern recognition receptors present on the cell surface (shown in blue), in the endocytic vesicles (shown in green) and cytoplasm (shown in red) of phagocytic cells. Humoral immunity is mediated by antibodies which can neutralize Cas9 protein or delivery vehicles. Both IgG and IgM antibodies have been seen to Cas9 exposure. Cellular immunity is mediated by display of peptides from intracellular proteins on cell surface receptors that can be recognized by cytotoxic T cells mediating killing of Cas9 expressing cells.

*In vivo* delivery of CRISPR, both Cas9 and other CRISPR systems, can be immunogenic through three main pathways; innate immunity, humoral immunity, or cellular immunity, illustrated in Figure 4 [158]. The innate immune system utilizes pattern recognition receptors to recognize conserved features in microbes, including the recognition of foreign nucleic acids [159]. This raises the possibility that mRNA or DNA encoding CRISPR or the gRNA could be recognized. Studies using *in vitro*-transcribed gRNA have been demonstrated to trigger the innate immune response in both human and mouse cells triggering interferon response and cell death [160,161]. This response could be tempered through the use of chemically synthesized gRNAs or through chemical modification of the *in vitro*-transcribed gRNAs [160,161]. While these studies show proof-of-principle, further studies are needed to identify the best method to avoid the innate immune response to both the gRNA, mRNA, and DNA delivery.

Beyond innate immunity, antibodies to *Staphylococcus aureus* Cas9 (SaCas9) and *Streptococcus pyogenes* Cas9 (SpCas9) have been found in human serum samples, suggesting a pre-existing immunity to Cas9 in a portion of the human population due to prior exposure to the two most currently used Cas9 sources, *S. aureus* and *S. pyogenes* [162,163]. These two studies found very different portions of the population to have preexisting antibodies, 10 or 78% to SaCas9 and 2.5 or 58% to SpCas9 [162,163], suggesting some variability either in the testing or populations. Despite these differences, it is clear that a portion of the human population has pre-existing antibodies to the two most commonly used Cas9 enzymes, which could affect the use of these Cas9 molecules *in vivo*. Furthermore, studies in animal models have shown that delivery of SaCas9 by AAV results in the development of anti-SaCas9 antibodies in adult mice after systemic delivery and in non-human primates after injection into the eye [115,143]. Similar to the findings with SaCas9 by AAV, systemic delivery of SpCas9 by adenovirus also resulted in development of anti-SpCas9 antibodies in adult mice [164]. The age of the animal appears to be important in the immune response development as systemic injection of neonatal mice with AAV-SaCas9 did not result in antibody development [143]. While the development of antibodies to Cas9 may not be problematic for single dose therapy with Cas9, it could prevent repeated use. As both pre-existing and developed immunity can occur, these factors may need to be monitored during clinical development of CRISPR therapeutics.

In addition to both innate immunity and humoral immunity, cellular immunity mediated by cytotoxic T cells can also be directed against Cas9-expressing cells. Examination of human blood samples has shown the presence of reactive T cells to both SaCas9 and SpCas9, with the percentage of responding population being 78% to SaCas9 and 67 or 96% to SpCas9, depending on the study [162,165]. Similar to the humoral immunity studies, there is variability in the percentage of responding humans, but it is clear that many people may have cells capable of killing Cas9 transfected cells *in vivo*. In addition, animal studies have shown the development of cellular immunity to Cas9 after AAV delivery of SaCas9 and adenoviral delivery of SpCas9 in mouse models and AAV delivery of SaCas9 in non-human primates [115,143,164]. As seen with antibody development, the age of the animal appears to be important, as systemic delivery of AAV-SaCas9 to neonatal mice did not cause the development of a reactive T-cell response [143]. The development of a T-cell response could be problematic, as it could result in the destruction of Cas9 expressing cells *in vivo*, abrogating the therapeutic effect and potentially result in the therapy exacerbating a condition through eliminating the population of cells targeted for editing. It is possible that this effect could be mediated by immune suppression, however this would not be ideal for long-term treatment, making controlled temporal presence of Cas9 important to prevent adverse immune effects.

The studies to date have shown that age may be an important mediator of the immune response. In addition to age, it is likely that the delivery vector will have a strong effect on the immunological response. In animal models, the delivery of Cas9 with adenoviral vectors, which are highly immunogenic, resulted in decrease in Cas9 expression overtime, no therapeutic effect, inflammation, and hepatocellular toxicity. In comparison, delivery of Cas9 by plasmid resulted in lower gene correction but therapeutic effect suggesting edited hepatocyte survival [158]. It is therefore important to determine and control the immune response to both Cas9 and the delivery vehicle for efficacious editing. One possible method to reduce the immunogenicity of Cas9 is the identify the epitopes leading to immune stimulation and engineer the Cas9 protein to create a low immunogenicity Cas9. Preliminary studies in this area were able to identify the reactive residues and alteration of these residues did not abrogate SpCas9 editing [164], suggesting the potential to reduce the immunogenicity profile of Cas9 and enhance its therapeutic use [158].

### Off-target effects

An ideal gene editor would exhibit perfect specificity for the target sequence and cause no mutations to any other region of the genome. Unfortunately, CRISPR/Cas-based editors (and other editors, such as ZFNs and TALENs) rarely achieve such a high standard. Off-target effects primarily arise from the sgRNA seed sequence or PAM sequences binding with sequence mismatches (1–5 bps have been shown to be tolerated) but can also be influenced by cell type and the DNA repair pathways in a particular cell type. The reader is referred to Zhang et al. [166] for a more thorough review of the mechanisms of CRISPR off-target effects. Off-target effects were postulated early in the development of gene editors and recent advances have increased the sensitivity for detection of these effects. As a result, off-target effects have been shown in virtually all systems studied.

The presence of off-target effects does not necessarily prevent gene editing tools from succeeding in clinical applications. Off-target modifications could be tolerated for many treatments if they were random, showing no sequence bias, and the levels were at or below the random mutation frequencies. Additionally, while off-target effects may have significant consequences in specific cell types, like stem cells or neurons, they could be more tolerable in cells that are fully differentiated or those with short replicative lifespans. Ultimately, the treatment risk must be weighed with disease prognosis and the potential benefit. Currently accepted clinical treatment methods for many diseases carry with them high risk including mutagenesis (e.g., cisplatin for cancer chemotherapy) [167]. High risk of off-target modifications could be accepted for difficult to treat diseases especially where the overall prognosis is poor, provided the possible mutation risks were known.

Strategies for optimizing CRISPR specificity through improved design have resulted in software tools for predicting off-target sites and probabilities ranging in complexity from threshold-based to machine learning-enabled. Assays for detecting CRISPR off-target mutations have traditionally attempted to identify the double stranded breaks created when Cas9 cleaves the genome with a combination of DNA sequencing and bioinformatics. The assays vary in their approach and can suffer substantial bias due to PCR amplification. Approaches for predicting and detecting CRISPR off-target effects developed prior to 2015 are adequately reviewed in Zhang et al. [166]. Most recently this topic has been updated and expanded upon to include developments up through 2018 in a review by Gkazi [168] which presents a concise summary of tools for quantifying off target modification from CRISPR/Cas9 in three categories, *in silico* (prediction), *in vitro*, and *in vivo* assays including advantages and disadvantages to each method*.* To avoid redundancy with these reviews, we limit our discussion here to several promising, newly emergent tools with potential for or direct application for assaying off-target effects in the clinic and refer the reader to Gkazi [168] for information on additional tools and the historical evolution.

One area of interest in off-target discovery are methods that can bridge both *in vitro* and *in vivo* gap in off-target detection. Joung et al. have demonstrated a pipeline that bridges *in vitro* and *in vivo* to enable improved nuclease-based therapeutic strategies in a recent publication [169]. Their system, coined Verification of In Vivo Off-targets or VIVO, invokes a two-staged approach where an initial ‘discovery’ step is performed *in vitro* to identify a large, inclusive set of potential off-target cleavage sites using Circularization for *In vitro* Reporting of CLeavage Effects (CIRCLE-seq). This initial step is followed by an *in vivo* confirmation step that identifies indel mutations in the potential set of sites. This pipeline is advantageous for examining the genome-wide specificities of CRISPR/Cas editors and identifying off-target effects. Although, not experimentally demonstrated in this initial publication, the approach is believed to be generalizable to non-CRISPR approaches and non-mammalian organisms (Supplementary discussion, [169]).

Corn et al. recently reported a new assay, Discovery of In Situ Cas Off-targets and VERification by sequencing (DISCOVER-seq), for detecting off-target modifications to the genome [170]. DISCOVER-seq operates differently than other off-target detection assays in that it monitors a protein involved in DNA repair, MRE11, to identify where genome edits have occurred rather than the double-stranded break itself. The use of a repair factor protein that is specifically and avidly recruited to a DSB site only during active DNA repair leads to a significant reduction in false-positives over prior methods like ChIP-seq that utilize Cas9 binding as a basis for detection. Importantly, MRE11 is broadly conserved across multiple species and expressed in many cell types. The method has been shown to be insensitive to differences in guide RNA formats and Cas enzyme orthologs. Together these features point to the potential of DISCOVER-seq to be a universal detection platform for identifying off-target mutations *in vitro* and *in vivo*.

Typically, assays for CRISPR/Cas genome edits are accomplished using a variety of targeted PCR amplification followed by next-generation sequencing or next-generation sequencing alone. These assays can be extremely sensitive to small edits such as indels, but inherently insensitive to detection of structural variations including large deletions, translocations, and inversions. Unfortunately, the potential for structural rearrangements are important to measure as chromosome rearrangements can cause alterations in gene expression leading to clinical symptoms or even the development of cancer [171,172]. Additionally, understanding the potential for structural alterations in chromosomes is required both for FDA approval and to the development of safe genome editors for clinical use. Recently, the Uni-directional Targeted Sequencing (UDiTaS) method was shown to successfully measure structural changes and indel events in one reaction [173]. The basis for the method is a custom Tn5 transposon, a unique molecule identifier (UMI) barcode, and a pooling barcode which are assembled and used to tagment genomic DNA. Following amplification of target regions, two rounds of PCR are performed (one using the target primer and the second creating the Illumina sequencing library). The method was shown to be highly linear and sensitive, though it could be limited by bias during the amplification. While the UDiTaS method is designed for use *in vitro*, the ability to look at specific cell types, including primary cells, can define specific large and small genome alterations to monitor in *in vivo* studies and can provide necessary supplemental information on large-scale genomic alterations caused by genome editors.

Similarly, CHAMP (Chip-Hybridization Association Mapping Platform), is an *in vitro* method that has been shown success profiling off-target CRIPSR-Cas binding on synthetic and importantly human genomic DNA [174]. CHAMP utilizes a Mi-Seq flow cell which following DNA sequencing is repurposed for use as a spatially addressable platform containing a library known (sequenced) DNA oligomers that can bind to Cas proteins. In combination with fluorescent reporters on the DNA and the Cas protein and high throughput total internal reflection fluorescence microscopy (TIRFm), each oligo on the chip is read out and the presence of both fluorescent labels indicates that DNA sequence can bind the protein. This method can thus provide quantitative profiling of off-target binding sites in the genomes of induvial patients in the clinic and with future development could also assay target cleavage in addition to binding.

Beyond off-target effects in CRISPR editing and DNA interaction, recent research has found unanticipated effects in protein expression and function after attempted CRISPR gene knockouts [175]. Combined RNA sequencing and triple-stage mass spectrometry was utilized to examine the protein expression of 193 DNA verified CRISPR-induced frameshift deletions and demonstrated protein expression in one third of the targets. Closer examination found the proteins were either N-terminally truncated or the product of skipping the editing exon, creating a different protein isoform. Detailed analysis of three of the truncated proteins found partial preservation of protein function. Additionally, recent studies have demonstrated that the tumor suppressor p53 can inhibit CRISPR engineering in human cells by causing toxicity and cell death as a result of DSB formation [176,177]. The inhibition of CRISPR activity by p53 is particularly concerning as this can promote selection of p53-defective cells with increased tumorigenic potential. Taken together, these studies and other studies of off-target effects reviewed elsewhere highlight the need to understand the biological effects of CRISPR use at the DNA, RNA, and protein levels to prevent unintended effects of CRISPR clinical use of current gene editors and the coming gene editing technologies.

## Coming solutions and novel gene editing technologies

Having surveyed the landscape of current CRISPR clinical trials, it is important to look forward to anticipate which of the novel CRISPR-based technologies currently in the pipeline are likely to be impactful in the clinic in the near future. In the following sections of this review, we identify and discuss a few areas of focus for future research that will likely have significant clinical impact.

### Hyperaccurate CRISPR systems

**Figure 5:**
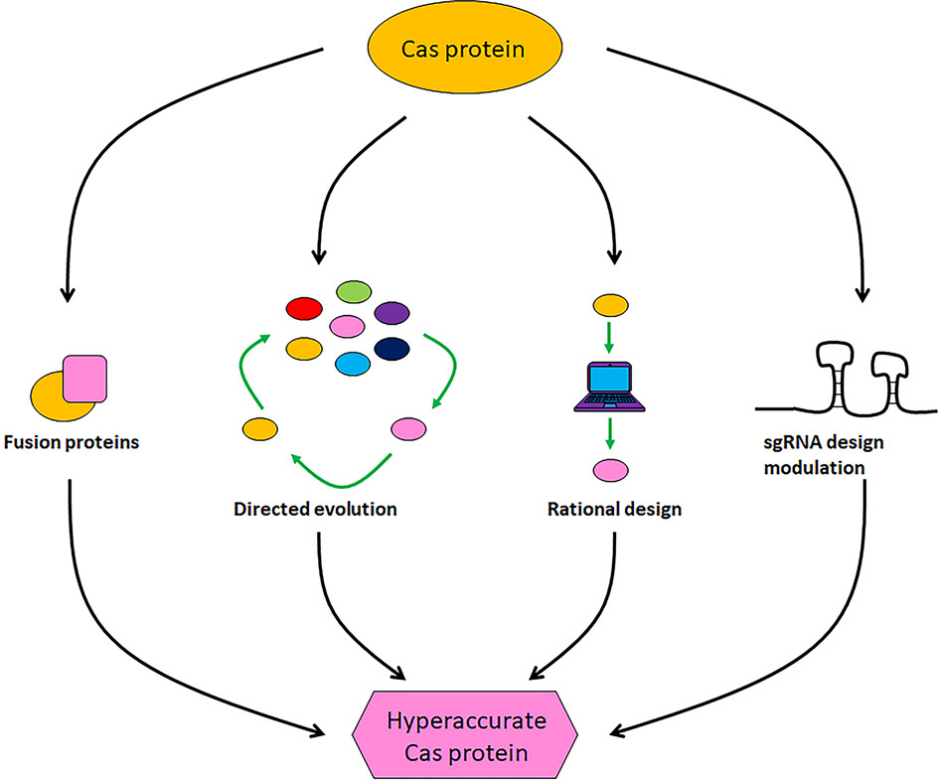
Engineering hyperaccurate CRISPR systems Several approaches have been used to improve the function of Cas9 proteins and reduce off-target effects. Cas9 function has been modified through the rational design and engineering of a higher fidelity nuclease, modifying the sgRNA for increased stability, directed evolution toward hyperaccuracy, or fusing Cas9 with programmable DNA-binding domains.

As previously discussed, off-target effects of CRISPR systems are caused by a range of factors, including levels of the CRISPR complex, sgRNA sequence, PAM sequence, and target genome structure/state [166,178,179]. We will discuss efforts to reduce off-target effects by addressing a number of these factors to generate hyperaccurate CRISPR systems (Figure 5).

When the domain structure and function of an enzyme is known, rational design can be an effective and inexpensive way to rapidly produce high-efficiency alternatives to the canonical protein. With SpCas9, a number of studies have elucidated the structure–function relationships within the protein, opening up the door for mutation by rational design [180–184]. Enhanced SpCas9 (eSpCas9) is an SpCas9 variant which neutralizes the positive charge of a non-target strand groove located between the HNH (Histidine Asparagine Histidine), RuvC, and PAM-interacting domains in SpCas9. This decreased non-target strand binding, likely allows for rehybridization between the target and non-target DNA strands, which preferentially prevents sgRNA–DNA hybridization at weaker, non-specific interactions [185]. Another example is SpCas9–HF1, which was designed by mutating four residues that are known to be involved in formation of non-specific DNA contacts. This resulted in a form of SpCas9 that retained over 85% of wild-type activity, but almost eliminated off-target effects [186]. In yet another study, the Doudna group demonstrate that a domain in SpCas9 of previously unknown function, REC3, binds to the RNA–DNA duplex, and enables the docking of the HNH nuclease domain at the active site. They also show that both eSpCas9 and SpCas9-HF1 contain mutations in the REC3 domain that block the HNH nuclease domain in the inactive state (also called the ‘conformational checkpoint’) when bound to mismatched targets. This led them to rationally design a new hyperaccurate SpCas9, called HypaCas9, with mutations in this same REC3 domain that trigger this conformational checkpoint (with the help of another domain, REC2) [187]. These studies demonstrate the usefulness of rational design experiments, where a few targeted mutations can yield significant gains in terms of specificity.

A limitation of rational design however is that, by definition, it is limited by what we know about the target protein, making it potentially limited in scope and inherently biased. The larger scale unbiased alternative to rational design is directed evolution which, while being a lot more resource-intensive and having a smaller rate of success, can yield novel and unexpected variants of a protein. For example, Casini et al. targeted the same domain as the Doudna group, REC3, for generating a hyperaccurate version of SpCas9, but instead of choosing residues to mutate based on known structure, they performed random mutagenesis over this domain and set up a large-scale screen in yeast. Ultimately, they combined four beneficial mutations to create evoCas9, which they show has superior activity and specificity to both wild-type and rationally designed Cas9 (eSpCas9 and SpCas9-HF1) [188]. Another group set up a one-step screen in *Escherichia coli* by introducing a target gene from the human genome (EMX1) into the *E. coli* genome, a mismatched version of the target on a plasmid encoding a toxin, a mismatched sgRNA to EMX1 and a library of SpCas9 mutants. This allowed for an elegant method to select SpCas9 mutants that caused cleavage of the toxin plasmid with the mismatched EMX1 (allowing for survival), but not the EMX1 in the *E. coli* genome (which would cleave the genomic DNA and cause cell death). Through this method, the authors identified a number of mutations not overlapping with any previously identified Cas9 variant and named their best mutant Sniper-Cas9 [157]. A similar method in *E. coli* used an on-target sequence on a toxin plasmid and an off-target sequence on an antibiotic resistance-containing plasmid, with three different human genes, including EMX1. The authors were specifically screening for Cas9 that retained activity and specificity in the context of the RNP form, which is the form with the most precise dose control for introduction of CRISPR components in the context of a therapy or clinical trial [189]. This revealed high-fidelity (HiFi) Cas9, containing a single point mutation (R691A) which the authors show resulted in a variant with high efficiency and specificity when delivered as an RNP in human CD34^+^ hematopoietic stem cells, compared with other high-fidelity Cas9 variants discussed here [189].

Phage-assisted continuous evolution (PACE) with three different PAM sequences on independent plasmids has also been used to expand the PAM specificity of SpCas9. The result, xCas9, harbors mutations near the DNA–sgRNA interface, and recognizes a wider range of PAMs while surprisingly also having decreased off-target effects compared with wild-type SpCas9 [190]. This result shows that directed evolution can expand the PAM specificity without sacrificing target specificity, suggesting that there are still many improvements to CRISPR systems that can be explored which we may not intuitively deduct. Methodically designed directed evolution, as exemplified in the studies above, are likely to be a critical component of designing increasingly efficient and accurate variants of CRISPR components, including other Cas proteins such as SaCas9 and Cas12. Each new study discussed here demonstrates that their version of Cas9 has the highest activity and accuracy compared with its predecessors, which is encouraging in terms of achieving a safe and accurate version of Cas9 that can reliably be used as a therapeutic. However, it is important to remember that there might be cell type- and delivery method-specific differences that can affect specificity and no comprehensive study comparing all of the Cas9 variants across all cell types and deliver methods has been performed. Therefore, each of these variants need to be individually tested for each desired application.

An alternative to introducing mutations in Cas9 to alter its function is creating fusions of Cas9 with other proteins such as programmable DNA-binding domains (pDBD), which can be used to enhance the specificity of Cas9. For example, early studies showed that a Cas9 fusion with the FokI nuclease (fCas9) requires the simultaneous binding of two monomeric fCas9s 15–25 bp apart for cleavage, which greatly reduces off-target effects [191,192]. This is similar to the ‘paired nickase’ approach, in which instead of having a single Cas9 nuclease, successful cleavage requires two mutated Cas9 proteins (which are nickases) localized by two independent sgRNAs [193–195]. A similar approach was utilized by Bolukbasi et al., where they examined the properties of fusions of SpCas9 with ZFPs and TALEs. Importantly, the authors simultaneously reduced the endogenous DNA-affinity of the Cas9, with the goal of broadening the sequence targeting range of the fusion protein, and show that this actually results in a significant increase in specificity [196]. Another interesting approach to increasing specificity of Cas9 is to create a fusion with chemically regulated proteins such as dihydrofolate reductase (DHFR) or the estrogen receptor (ER50), which are stabilized by trimethoprim (TMP) or 4-hydroxytamoxifen (4-HT), respectively. In the absence of these chemicals, the fusion proteins are targeted for degradation, thus reducing off-target effects by limiting the availability of the protein [197]. One disadvantage of fusion proteins is that they tend to be rather large, which can complicate delivery. Fusions of smaller Cas proteins (such as SaCas9 or Cas12a) with proteins that enhance targeting specificity could therefore be worth exploring. Fusion proteins are also further discussed in the context of base editors later in this review.

Until now, we have primarily focused on alterations to the protein component of CRISPR systems with the goal of increased specificity. However, parallel and equally justifiable efforts to increase the accuracy of CRISPR systems involve changing the structure of the sgRNA. The rules of sgRNA design are constantly evolving to improve specificity, with multiple groups contributing to this effort [195,198–200]. In addition to sequence, the chemical composition of the sgRNA can also affect specificity. Multiple groups have shown that introducing RNA modifications such as 2′-O-methyl-ribonucleotides, 2′-fluoro-ribonucleotides, phosphorothioate ribonucleotides and others, can increase the stability of the sgRNA by conferring resistance to nucleases, but also improve specificity by affecting the interactions between the sgRNA, DNA, and Cas9 [201–205]. Yet another approach to sgRNA optimization involves introduction of secondary structure that can affect specificity. Recently, a study showed that engineering in a hairpin at the 5′ end of the sgRNA can alter secondary structure and thereby provide an energetic barrier to R-loop formation. This allows for greater specificity and fewer off-target effects [206]. The authors also show that this method can improve the specificity of *S. aureus* Cas9 and Cas12a, suggesting broader applicability for altering sgRNA design [206]. Each of the individual approaches to Cas optimization reviewed above have yielded significant improvements both in specificity and efficiency of CRISPR gene editing. It will be interesting to determine whether combinations of these solutions could synergize to produce even more hyperaccurate RNPs.

Looking to the future, there are hybrid CRISPR/transposase systems that can be co-opted in a similar fashion to those mentioned above for use in mammalian cells. Recent work has shown the natural occurrence of such systems in bacteria allowing for incorporation of longer fragments of DNA into the genome without the need for homology-directed repair (which requires double-stranded DNA breaks) [207,208].

### Base editors

The CRISPR/Cas system has been successfully used to for homology-directed recombination (HDR)-based editing of the genome. This function is critical for correcting disease-causing mutations, but the low efficiency of HDR compared with non-homologous end-joining (NHEJ) in mammalian cells complicates using this method for repairing mutations [209]. Efforts to enhance usage of HDR, while significant and impactful, have only yielded moderate increases in efficiency [74,210,211]. An elegant solution to this problem was developed by David Liu of Harvard University, who demonstrating that fusing a cytidine deaminase (enzyme which deaminates a cytosine into a uracil) with catalytically inactive Cas9 (dCas9) could result in sgRNA-guided correction of a range of C to T mutations that are relevant to human disease [212]. This technology bypasses the need to make double-stranded breaks in the underlying DNA. Instead, the presence of a stretch of ssDNA in the R-loop of the dCas9 serves as a substrate for the cytidine deaminase (in this case, rat APOBEC1, which showed the highest activity of the four enzymes tried). Importantly, in contrast with the low efficiency of HDR, deamination-driven base editing showed 15–75% efficiency with minimal indels.

The Liu lab has since taken this technology a step further and developed a parallel adenine base editor system that can convert A•T into G•C in DNA. As there are no known adenine deaminases *in vivo*, this endeavor required directed evolution and engineering of a transfer RNA adenosine deaminase to be able to use DNA as a substrate [61]. The collection of modular base editing complexes developed by the Liu lab now enables all four transition mutations (purine to purine or pyrimidine to pyrimidine). Since these discoveries, a number of groups have developed and/or utilized similar dCas9/deaminase base editor systems, and shown efficient base editing in organisms ranging from bacteria to organoid systems to human embryos [22,49,62,93,213–219], which is extremely promising for therapeutic applications. More recently, BE-PACE (base editors through PACE) was used to identify and optimize base editors on both GC and non-GC target cytosines. They were able to successfully evolve three deaminases – APOBEC1, FERNY, and CDA1, and showed that fusion of these evolved proteins with dCas9 enhanced base editing efficiency compared with their parental proteins [220]. The authors do caution, however, that editing efficiency is subject to cell type, cell state, and site specificity, meaning that each individual application may require pilot testing of a variety of base editors before the optimal one can be chosen. In addition, base editors have also been used for *ex vivo* applications such affinity maturation of antibodies [221].

As with all novel technology however, careful evaluation is required to avoid any unintended off-target effects. Two recent studies demonstrate that base editors, specifically cytosine base editors, cause an alarming number of unwanted, off-target mutations *in vivo*, both in plants and mice [222,223]. Surprisingly, a new study found unwanted mutations in the RNA transcriptome as a result of off-target DNA base editing activity [224]. Of course, some of these effects could potentially be mitigated by reducing the dose of the base editing complex that is introduced, or by refining the components of the complex. Such efforts are already underway, with examples including the use of Sniper dCas9, a Cas9 with increased specificity created by directed evolution, or RNPs instead of plasmids to reduce to delivered dose [157,225]. Furthermore, many of these off-target mutations may either be silent on non-consequential functionally, but these findings highlight the need for further research before base editors can be widely used in the clinic.

### RNA targeting

While the development of CRISPR systems to target DNA across cell types and organisms for a variety of functions has undeniably revolutionized both basic scientific research and the development of therapeutics for clinical applications, the paramount importance of coding and non-coding RNAs in biology has made the discovery of RNA-targeting CRISPR systems an equally exciting area in the field of nucleic acid editing. Targeting of RNA by CRISPR systems is not only useful for altering levels of mRNAs and non-coding regulatory RNAs, but also represents a mechanism by which these edits can be temporally regulated, limited by the half-life of the targeted RNA (as opposed to genomic changes, which are permanent for the lifetime of the cell). While there are a number of known RNA-targeting CRISPR systems in bacteria, their adaptation for use in mammalian cell types is only now beginning to be explored [226]. There are both Class 1 (multi-component) and Class 2 (single component) RNA-targeting CRISPR systems [226]. Given the complexity of Class 1/Type III CRISPR systems and the resulting difficulty in expressing functional protein complexes heterologously, the majority of uses cases for these have been in prokaryotes [226]. On the other hand, Class 2 systems have been shown to target RNA, and are being successfully adapted for RNA-targeting in mammalian systems. Early work demonstrated that in the presence of a ssDNA oligonucleotide with a PAM sequence (PAMmer), SpCas9 could be ‘tricked’ into targeting ssRNAs for cleavage [227]. Using a similar PAMmer-based strategy, dSpCas9 can be targeted to various mRNAs for live tracking of transcripts within the cell [228]. More recently, it has been demonstrated that while the PAMmer enhances RNA-targeting of SpCas9, it is not required for this activity, as exemplified by the cleavage of repetitive RNAs and certain mRNAs [229,230].

*S. pyogenes* is however, not the only organism with a Type II Cas9 that can be made to target RNA, and in fact, Cas9 from alternative sources have been shown to be amenable to RNA-targeting. Cas9 enzymes from *Staphylococcus aureus* (SaCas9), *Campylobacter jejuni* (CjCas9), *Francisella novicida* (FnCas9) and *Neisseria meningitidis* (NmCas9) have all been shown to have RNA-targeting activity to different extents [231–235]. NmCas9 has been demonstrated to target RNAs *in vitro* in the absence of any PAMmer or cofactors, although its endogenous activity *in vivo* remains to be determined [233]. CjCas9 on the other hand, has been shown to target endogenous mRNAs *in vivo* through imperfect pairing with native crRNA guides and subsequent cleavage by Cas9’s HNH domain [231]. A step further is heterologous use of Cas9’s RNA-targeting activity, as shown in one study where SaCas9 had a protective effect in *E. coli* from MS2 phage through cleavage of its RNA genome, as well as targeting of endogenous *E. coli* mRNAs [234]. When it comes to adaptation of these Type II Cas9s to a mammalian system, one of the few demonstrations is the use of FnCas9, which not only can target endogenous RNAs, but has been shown to target the genome of a human RNA virus, hepatitis C, in a liver carcinoma cell line [232]. If these Type II Cas9 enzymes are to be used clinically as RNA-targeting enzymes, future work should focus on efficiency and specificity (both in terms of sequence, and DNA vs RNA) in mammalian cells, with significant focus on off-target effects, as with all the other novel CRISPR systems that are discussed herein.

The issue of fine-tuning a CRISPR system to target RNA instead of DNA can be a challenge with dual-specificity Cas proteins. Another member group of the Class 2, single component CRISPR systems, the Type IV Cas13 proteins, helps bypass this difficulty by specifically targeting only RNA (not DNA). Two of the best characterized Cas13 proteins are *Leptotrichia wadei* Cas13a (LwaCas13a) and *Leptotrichia shahii* Cas13a (LshCas13a, previously known as LshC2c2), both studied by Abudayyeh et al [236]. Heterologous expression of LshCas13a was shown to degrade *E. coli* transcripts *in vivo*, as well as protect *E. coli* from MS2 phage infection [236]. LwaCas13a was expressed both in plant and mammalian cells, and successfully targeted endogenous and reporter genes in both these cell types [237]. Both these Cas13a proteins exhibit ‘collateral RNA cleavage’, meaning that their RNase activity causes degradation of any surrounding transcripts, and this collateral RNA cleavage is not specific for the target. This problem is circumvented by using Cas13b *Prevotella sp. P5-125* (PspCas13b), as shown by Cox et al., which exhibits specific RNase activity for its target without the collateral damage [238]. This study then uses a catalytically inactive version of PspCas13b fused with ADAR2 (adenosine deaminase acting on RNA type 2) for highly specific base editing of RNA from adenosine to inosine (A to I). The authors coin their system RNA Editing for Programmable A to I Replacement, or REPAIR, and further engineer the system for optimal viral delivery [238]. Future work will likely seek to expand the repertoire of based changes that can be achieved, for example, C-to-U editing by using a fusion of Cas13 with APOBEC [239].

Another group developed a systematic computational method to identify more RNA-targeting Type IV CRISPR systems, following which they engineered seven Cas13 orthologs, which resulted in a ribonuclease effector derived from *Ruminococcus flavefaciens* XPD3002 (CasRx) [240]. CasRx was used to knockdown a variety of endogenous transcripts and mammalian cells with enhanced efficiency and specificity compared with RNA interference (RNAi) and even CRISPR-mediated inhibition (CRISPRi) [240]. In an elegant demonstration of the therapeutic potential of their work, the authors show that CasRx can regulate the splicing of MAPT, the gene encoding the tau protein. The ratio of τ isoforms is critically mis-regulated in Frontotemporal Dementia, and CasRx can rescue this ratio in diseased cortical neurons, suggesting that this protein could potentially be used therapeutically in primary cells such as neurons, to reverse the effects of neurodegenerative disease [240]. This is also an excellent example of a situation in which DNA editing would be of no use, as splicing occurs at the level of RNA. Of course, before such a therapeutic can be deployed in patients, extensive work has to be done to eliminate off-target effects and ensure long-term safety, but taken together, these RNA-targeting CRISPR systems represent a new and exciting frontier in the clinical applications of gene editing.

### CRISPR-mediated repression of gene expression

**Figure 6:**
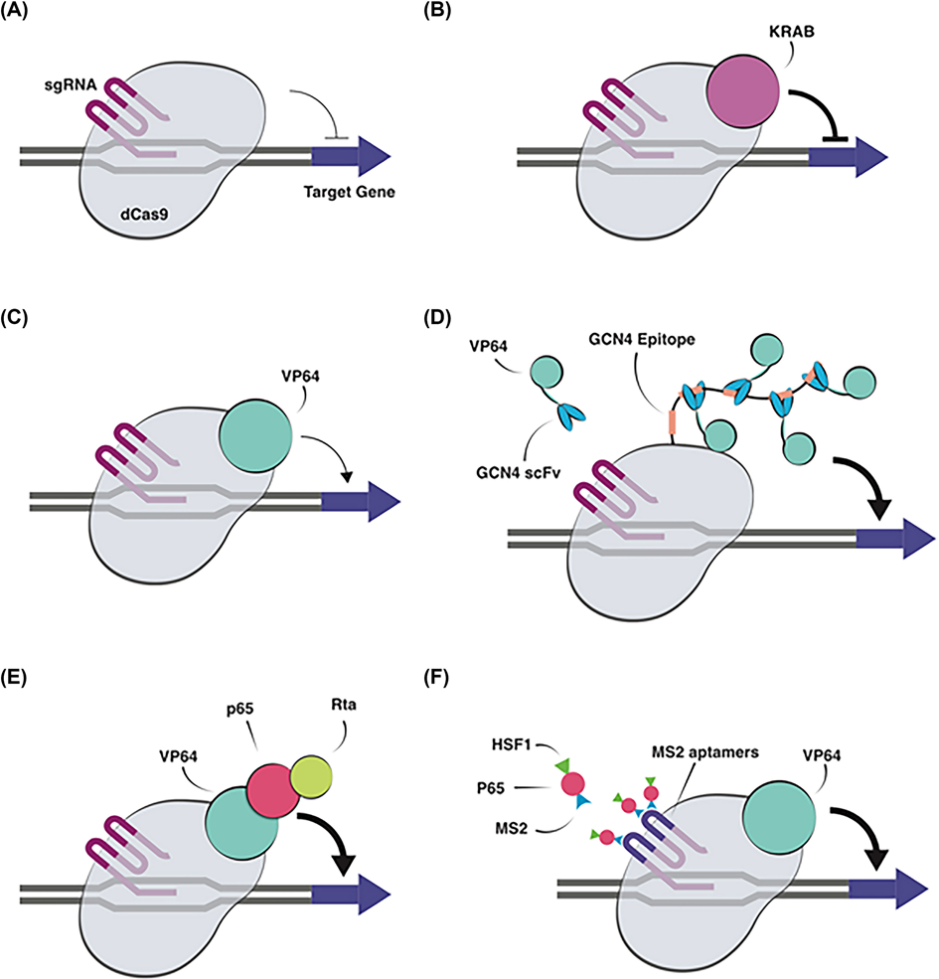
CRISPR/Cas9 regulators of gene expression (**A**) The nuclease dead version of Cas9, dCas9, is directed to a genomic locus with an sgRNA and inhibits transcription of a target gene. (**B**) Fusion of the KRAB domain to dCas9 causes stronger gene repression. CRISPR-mediated gene activation (CRISPRa) involves recruiting transcriptional activators to a genomic locus using a dCas9–sgRNA scaffold. (**C**) The simplest CRISPRa system is dCas9 fused to VP64. More complex CRISPRa systems include (**D**) Sun-Tag, (**E**) VPR and (**F**) SAM, and these involve the recruitment of multiple transcriptional activators to further enhance gene expression. Abbreviations: KRAB, Krüppel-associated box domain of Kox1; SAM, Synergistic Activation Mediator.

Recent modifications to the CRISPR/Cas9 system have provided effective strategies for targeted control of gene expression, as opposed to editing of nucleic acid sequences. Central to CRISPR-mediated gene regulation is a mutated form of Cas9 that lacks endonuclease activity, called dead cas9 or dCas9. The dCas9 protein incorporates point mutations (D10A and H840A) in the RuvC and HNH nuclease domains, respectively [16]. Importantly, although dCas9 does not cleave DNA, it retains the ability to precisely bind specific DNA sequences through sgRNA targeting [241]. Targeted gene expression can be repressed by directing dCas9 to the promoter or protein coding region of a gene. When bound to a promoter, dCas9 blocks transcription initiation through the steric inhibition of transcription factors and RNA polymerase [241] (Figure 6A). Alternatively, dCas9 bound to the protein coding region of a gene blocks transcription elongation when RNA polymerase collides with the dCas9-sgRNA complex [241]. Utilizing dCas9, or dCas9 fused to additional transcriptional repressors, to repress gene expression is the central tenet of CRISPRi.

Although dCas9 itself can be a powerful repressor of gene expression in bacteria, its ability to silence genes in mammalian cells is more modest [241,242]. Control of eukaryotic gene expression includes additional layers of regulation involving recruitment of positive and negative transcriptional regulators, as well as epigenetic modifications to genomic DNA and histones. To more effectively knock down transcription in mammalian cells, the dCas9 molecule was further engineered to incorporate fusions with various effector protein domains as illustrated in Figure 6A,B. Several fusions proteins were tested initially including dCas9 fused to the Krüppel-associated box domain of Kox1 (KRAB), the chromo shadow domain of HP1α (CS), the WRPW domain of Hes1, and four copies of the SIN3A domain of MAD1 (SID4X) [242,243]. Of the dCas9-repressor constructs tested, the dCas9-KRAB fusion was shown to be most effective at repressing eukaryotic gene expression, and could knock down reporter and endogenous genes 5–15-fold in human cells [242] (Figure 6B). The KRAB domain is broadly conserved across eukaryotic C2H2 ZFPs and exerts gene repression by interacting with chromatin-remodeling factors [244,245]. The dCas9-KRAB system has become the gold-standard for mammalian gene knockdown studies, and has been used for diverse purposes including mapping human gene regulatory networks [246], tuning gene expression in human pluripotent stem cells [247–249], and silencing gene expression within a mouse brain [250].

New CRISPR interference technologies continue to emerge, and novel modifications to the dCas9 scaffold have been shown to enhance the efficacy of gene silencing. A recent study screened numerous effector domains fused to dCas9 and found that a synergistic fusion of dCas9-KRAB-MeCP2 could produce a 5–60 fold reduction in gene expression when compared with dCas9 alone [251]. These CRISPRi tools allow rapid knockdown of genes across diverse cell types and can silence regions of the genome that were historically difficult to target including non-coding RNAs, antisense transcripts and microRNAs.

### CRISPR-mediated activation of gene expression

The foundation of CRISPR-mediated gene activation (CRISPRa) is the dCas9 protein fused to one or more transcriptional activators. There are several CRISPRa systems currently in use (Figure 6C–F), and each builds a unique synthetic transcription factor that can be targeted to the promoter of a gene of interest with an sgRNA. The first version of CRISPRa involved dCas9 fused to VP64, a strong transcriptional activator composed of tetrameric repeats of the activation domain of herpes simplex viral protein VP16 [193,242,252,253] (Figure 6C). The use of dCas9-VP64 has been shown to allow activation of target genes, although the level of gene expression is typically a modest two to five fold increase in mammalian cells [254]. Interestingly, multiplexing several sgRNAs that tile the promoter enhances gene activation, suggesting that recruitment of multiple dCas9-VP64 activators could increase gene expression [252,255]. To recruit multiple copies of VP64 to a genomic locus, a novel CRISPRa system termed SunCas9, or SunTag, was developed [256,257] (Figure 6D). The SunTag module involves dCas9 fused to a protein scaffold composed of 10–24 repeating GCN4 peptide epitopes [256,257]. The SunTag epitope array then recruits multiple copies of the nuclear localized GCN4 scFv antibody fused to a VP64 activator. Using the SunTag system a single dCas9 molecule is capable of recruiting numerous copies of VP64 to a target sequence, and considerably increases gene expression when compared with activation with the dCas9–VP64 fusion alone [257].

The next iterations of CRISPRa technologies involved combining multiple synergistic transcriptional activators with the sgRNA-directed dCas9. In one study, researchers surveyed the effects of numerous activation domains fused individually, and in combination, to dCas9 to increase gene activation [258]. A tripartite activator complex composed of VP64, p65, and Rta fused in tandem, called VPR, was shown to increase gene expression of endogenous targets 22–320 fold when compared with dCas9–VP64 only [258] (Figure 6E). Another CRISPRa approach called the Synergistic Activation Mediator (SAM) builds on the dCas9–VP64 fusion protein by adding multiple, distinct activation mechanisms [259] (Figure 6F). Analysis of the crystal structure of the Cas9–sgRNA complex revealed that both the tetraloop and stem-loop 2 of the sgRNA have four base-pairs that inertly protrude outside of the Cas9 protein [183]. The exposed sgRNA loops were hypothesized to be capable of acting as scaffolds to recruit additional transcriptional activators. The SAM system utilizes the original dCas9–VP64 protein but incorporates an engineered sgRNA that contains minimal hairpin aptamers appended to the sgRNA tetraloop and stem-loop 2 [259]. The hairpin RNA aptamers can be selectively bound by an MS2 bacteriophage coat protein [260]. The MS2 protein is then fused to two additional transcriptional activators, p65 and the activation domain from the human heat-shock factor HSF1 [259]. Each sgRNA aptamer can bind two copies of the MS2 protein, thus allowing a total of four copies of MS2-p65-HSF1 fusion proteins to be recruited to each dCas9–VP64 activator complex [259]. Together, the SAM system utilizes a synergy of three distinct activation domains (VP64, p65, and HSF1) to generate a specific and powerful CRISPRa tool capable of activating genes more strongly than dCas9–VP64 by itself. CRISPRa technologies continue to be improved, but currently the SunTag, VPR, and SAM systems function relatively equally in terms of gene activation [261]. Importantly, across diverse eukaryotic species, the SunTag, VPR, and SAM systems are all capable of gene activation several orders of magnitude stronger than the first-generation dCas9-VP64 alone [261].

While most studies utilizing CRISPRa technologies in disease models have been focused on understanding how genes function at a cellular level, several studies have looked at the potential to use CRISPRa to modulate disease in mouse models. Use of CRISPRa systems resulted in increased gene expression that led to phenotypic changes in the mice, including mouse models of diabetes, muscular dystrophy, and acute kidney disease [262]. Recently, delivery of dCas9-VP64 targeted down-regulated genes in breast cancer resulted in increased gene expression and reduction in tumor growth in mouse xenografts [263]. Viral-based delivery of dCas9–VP64 has also been demonstrated to overcome haploinsufficiency in two obese mouse models. The resulting increase in expression from the existing normal copy of two different genes, Sim1 or Mc4r, prevented the development of obesity in the mouse models [264].

### Epigenetic modifications using CRISPR tools

The CRISPRi/a tools described above primarily function by directly recruiting or interfering with transcriptional machinery which is thought to result in short term alteration of gene expression limited to the duration of the CRISPRi or CRISPRa expression. However, most eukaryotes utilize additional epigenetic modifications to DNA and histones that are important regulators of gene expression. One of the most well-studied epigenetic modifications is the methylation of the fifth position of cytosine. In mammals, methylation can be deposited *de novo* by DNA methyltransferase (DNMT3A/B) and can be maintained by DNMT1 [265]. Demethylating DNA involves oxidation of the methyl group by TET dioxygenases, and restored by replication-dependent dilution or DNA glycosylase-initiated base excision repair [266]. Methylated DNA regulatory elements interfere with their trans-acting transcription factors causing gene repression [267]. The ability to edit the methylation at specific genomic loci would be a powerful capability to enable researchers to better control transcription in mammalian cells.

Targeted editing of DNA methylation has previously been accomplished with custom ZFPs or TALEs fused to DNA methyltransferases [268,269]. However, designing new ZFPs or TALEs for every target sequence of interest would be expensive and laborious. Alternatively, several CRISPR/Cas9 tools have recently been engineered that allow editing of DNA methylation, and only require a custom sgRNA for targeting to genomic loci of interest. The first generation of CRISPR-based methylation systems in mammalian cells used dCas9 fused to full-length DNMT3a or the DNMT3a catalytic domain [270–273]. Importantly, these dCas9-based methylation tools have been shown to function with higher efficacy and resolution, and also more effectively silence gene expression than the TALE-based approaches [271–273]. The dCas9-DNMT3A fusions can accurately methylate DNA across a region of ∼35–320 bp from the DNA-bound fusion protein and can be multiplexed with several sgRNAs to span a larger target region of the genome [270–272]. Alternatively, methylation across a larger genomic region can be edited using the dCas9-SunTag-DNMT3A system, that can recruit multiple DNMT3As to a target site and hypermethylated a region of ∼4.5 kb [274,275]. Multimerization can also improve methylation and using dCas9-DNMT3a/DNMT3L could lead to more potent and widespread DNA methylation of CpG islands, and additionally caused DNA methylation spreading from a target site [276]. Importantly, it is heritable across mitotic divisions [270].

A limitation to the first-generation dCas9-DNMT fusion proteins is that they function relatively slowly over a period of several days, which may impede experimental systems that require analyses over shorter time durations. Additionally, recent analyses of genome-wide methylation patterns arising from dCas9-DNMT systems identified numerous off-target effects that are common in promoters, CpG islands and 5′ untranslated regions [277,278]. Off target effects of dCas9-DNMT3A may arise because dCas9 is not required to be bound to its sgRNA-target sequence in order to function, so dCas9-DNMT3A bound to other genomic loci are free to promote methylation [279]. Additional off target effects may arise because DNMT3A functions as a tetramer and can recruit endogenous DNMT3A/DNMT3L proteins to methylate larger regions [270]. Several recent iterations of dCas9 DNA methyltransferases have attempted to increase enzyme kinetics and resolve off target effects. To increase the rate of DNA methylation, new CRISPR tools incorporated an engineered *de novo* CpG DNA methyltransferase from the bacterial species *Mollicutes spiroplasma* strain MQ1 (M.sssI) [280]. The modified MQ1^Q147L^ DNA methyltransferase is relatively small in size (386 amino acids), functions rapidly within 24 hours, and produces fewer off-target effects [280]. The dCas9-MQ1^Q147L^ fusion protein was demonstrated to efficiently methylate target DNA sequences in human cells and developing mouse embryos [280]. Bacterial derived MTases are not expected to recruit endogenous mammalian MTases which may help to limit off-target demethylation. To further reduce off-target events, a split version of the M.sssI MTase was shown to generate efficient and targeted DNA methylation (up to ∼70%) [279]. The split MTase system involves one fragment of M.sssI fused to dCas9 which then directs the assembly of the MTase fragments when bound to target DNA loci [279]. Additionally, this system further reduces off target events because the two M.SssI fragments lack stability or ability to methylate DNA when dCas9 is not bound to DNA [279].

Targeted demethylation in mammalian genomes has also been accomplished with several technical variations, but all methods described here involve recruiting the TET1 dioxygenase to specific genomic loci guided by sgRNA and dCas9. Similar to the methylation strategies described above, targeted demethylation was previously demonstrated using TALE-TET1 fusion proteins in human cells [281]. CRISPR-based demethylation editors subsequently emerged that involved protein fusions with dCas9 and the catalytic domain of TET1 (TET1CD), and these dCas9-TET1 systems both demethylated target DNA and activated gene expression in cancer cell lines, mouse embryonic stem cells, mouse cortical neurons, and *in vivo* in mice [272,282–284]. The dCas9-TET1 fusion protein was directly compared with a TALE-TET1 fusion, and the CRISPR-based approach was determined to demethylate DNA more effectively and accurately than the TALE-based approach (28% decreased methylation within 150 bp vs. 14% within 200 bp, respectively) [272]. A second CRISPR-based demethylation method involved repurposing the SunTag system to recruit multiple TET1CD molecules to a genomic locus [283]. The SunTag-TET1 system was capable of more long-range demethylation when compared to TALE-based methods, likely because up to ten copies of TET1 could be recruited to a target sequence [283]. The SunTag-TET1 system was also shown to more effectively demethylate DNA than the direct dCas9-TET1CD fusion protein (>90% max observed demethylation vs. 14%, respectively) [283]. Importantly, in addition to demethylating DNA, the SunTag-TET1CD was also demonstrated to modify gene expression, and could induce a 1.7–50 fold up-regulation of target genes in diverse mammalian cell types and *in vivo* in brain of mouse fetuses [283]. Finally, a third demethylation approach repurposed the SAM system to recruit MS2-TET1CD to the MS2 aptamers protruding from the dCas9–sgRNA complex [284]. The MS2-TET1CD demethylation system was also shown to promote demethylation and activate gene expression, although it was not as effective as the SunTag system [283,284]. Together there are many variations of CRISPR-based DNA methylation editors, however in order to construct a desired epigenetic landscape, the cooperation between methylation and demethylation editors is likely necessary. For example, a recent study using mouse embryonic stem cells demonstrated that a synergy between TET1 and DNMTs was necessary to properly define and edit regions of genome methylation [285].

Altering the state of chromatin through modifications to histone proteins is another epigenetic mechanism to control proper gene expression in eukaryotes. Numerous histone modifications are involved with directing mammalian gene expression, but the majority of current CRISPR-based histone editing involves acetylation or methylation of lysine residues. When terminal lysine residues of histone proteins are acetylated, their charge becomes more neutral which weakens the interaction between histones and DNA and can subsequently become more permissive for transcription [286]. Permissive and repressive chromatin states are mediated in part by histone acetyltransferases and histone deacetylases, and these enzymes have been repurposed into novel CRISPR tools. The first CRISPR-based histone modifier was dCas9 fused to the catalytic core of the human histone acetyltransferase p300 (dCas9^p300 Core^) ^[^287^]^. Notably, use of dCas9^p300 Core^ could significantly increase target gene expression in human cells due to acetylation of H3K27 residues from gene promoters as well as both proximal and distal enhancers [287]. Additionally, the dCas9 transcriptional activators described above typically require the recruitment of multiple effector domains or multiple sgRNAs to promote robust gene expression, while the dCas9^p300 Core^ system needs only a single gRNA to function effectively [287]. CRISPR-mediated histone deacetylation tools have also been developed using dCas9 fused to the full-length human histone deacetylase HDAC3 [288]. Importantly, the dCas9-HDAC3 fusion protein was demonstrated to remove H3K27ac marks when positioned properly in a gene promoter, and could modify gene expression in mouse and human cell lines [288]. Unlike the dCas9-based transcriptional activators/repressors, the positioning of the sgRNA relative to the transcription start site of a gene does not appear to influence function, however the positioning relative to endogenous histone acetylation was crucial [288].

Another modification to histone proteins that regulates chromatin accessibility and local gene expression is the methylation of lysine residues. Histone modifying enzymes including methyltransferases and demethylases are conserved proteins that control histone methylation and can be engineered to be used as programmed histone-modifying enzymes. Recent developments to the CRISPR-based epigenetic toolbox includes both histone demethylases and methyltransferases fused to dCas9. The human protein lysine-specific histone demethylase (LSD1) is a broadly conserved lysine-specific histone demethylase and transcriptional co-repressor, and silences enhancer elements through H3K4/K9 specific demethylation [289–291]. An engineered TALE-LSD1 was shown to edit active enhancers and modify gene expression [292]. Soon afterward, a dCas9-LSD1 fusion protein was developed that could allow for more high-throughput histone demethylation editing [293]. The dCas9-LSD1 protein was demonstrated to effectively remove histone methylation from enhancer regions and knock down gene expression in mouse embryonic stem cells [293]. Alternatively, methylation of specific lysine residues on histone 3 proteins (e.g., H3K4me3 and H3K79me2-3) are associated with transcriptionally active euchromatin [291]. PRDM9 is a histone lysine methyltransferase that possesses mono-, di-, and trimethylation activities at H3K4 residues [294]. The functional domain of the human PRDM9 was recently fused to dCas9, and could produce targeted H3K4me3 marks at promoter regions that activated expression from previously silenced genes in human cell lines [295]. Methylation of other specific histone residues can alternatively create repressive chromatin (e.g., H3K9me3 and H3K27m3) leading to gene silencing [291]. Recently, O’Geen et al. engineered a broad set of epigenetic writers and recruiters fused to dCas9, and investigated their ability to edit H3K9me3 and H3K27me3 marks and to modify gene expression [296]. Interestingly, this work demonstrated that all of the methyltransferases tested (EZH2, G9A, SUV39H1, and FOG1) were all capable of some level of transcriptional repression, although they did not always correlate with expected histone modifications [296]. Additionally, some of the epigenetic editors tested only provided temporary silencing, but combinations of effectors could overcome this transient effect and promoted long-lasting repression [296]. Engineering a desired and persistent cell state is a complex and challenging effort and will likely require the use of multiplexed epigenetic modifiers targeted to both enhancer and promoter regions.

Similar to the status with CRISPRi and CRISPRa technologies, the application of Cas9 epigenome editors has mostly been pursued *in vitro* to date. However, the potential to selectively silence a mutated gene or activate the healthy gene copy with long duration effects has vast potential in medicine that could be achieved as CRISPR epigenome editing technology advances.

## Conclusions

The rapid development of gene editing technologies within the last decade is already providing significant advances toward improving human health. Gene editors are being used in ongoing clinical trials to treat diverse human diseases including HIV, cancer, and blood disorders. As gene editing tools continue to evolve, novel therapies for additional diseases will likely emerge. CRISPR-based gene editing tools, in particular, are quickly developing and have been used to generate diverse modifications in mammalian cells, including targeted editing of specific DNA sequences, activation or repression of genes of interest, and epigenetic reprogramming of cellular identities. However, despite the potential benefits of using gene editing technologies for human therapy, the fundamental biology underlying these technologies needs to be better understood in order to provide safe and effective treatment options to patients. Many of the CRISPR tools have been tested only *in vitro* and an outstanding question is the efficacy and safety during *in vivo* applications. This will likely involve the complex interplay between a given tool’s molecular function combined with the delivery modality. Some CRISPR components have been immunogenic in certain individuals – how can we engineer these potential therapies to minimize the risk of eliciting a counterproductive immune response? CRISPR gene-editing tools often exhibit widespread off-target effects, which could be dangerous if these therapies are needed in vital organs or are delivered there unintentionally – how can we maximize delivery to targeted body sites and minimize accumulation at off-target sites? These issues are all currently being investigated by research groups across the globe, and improvements in these areas will be crucial for the success of gene-editing therapies.

Ethical issues also arise from this recent wave of new gene-editing tools. Is it ethical to edit a developing human embryo? Who should make these decisions, and who will regulate them? There will need to be global discussions bridging science and politics to govern the use of CRISPR and gene editing in developing babies. Furthermore, health and disease are often a spectrum rather than a binary condition, and decisions must be made on the basis of known or likely trade-offs. Gene editing carries significant risks, such that a balance between acceptable risk for significant benefit must be struck in each case of potential use. This balance will shift as the technology advances, changing the risk/benefit profile for a given therapy.

Finally, as new technologies emerge there is always the possibility that they will be accidentally or intentionally misused. Ongoing work to identify and engineer anti-CRISPRs, such as that comprising DARPA’s Safe Genes program, is already revealing the path forward to development of countermeasures that inhibit or reverse unwanted gene editing. Gene editing technologies are extremely powerful and hold immense potential to provide new opportunities to treat a myriad of human diseases. As the amount of resources devoted toward better understanding and characterization of these technologies continues to increase dramatically with every passing year, their full-fledged clinical deployment appears very close to becoming a reality.

## Abbreviations

AATD: α-1 antitrypsin deficiency
AAV: adeno-associated virus
AAVS1: adeno-associated virus integration site 1
ACT: adoptive T-cell transfer
B2M: β-2 microglobulin
CAR: chimeric antigen receptor
CasRx: *Ruminococcus flavefaciens* XPD3002
CF: cystic fibrosis
CGD: chronic granulomatous disease
CjCas9: *Campylobacter jejuni* Cas9
CORD6: cone–rod dystrophy
CRISPR: clustered regularly interspaced short palindromic repeat DNA sequences
CRISPRa: CRISPR-mediated gene activation
CRISPRi: CRISPR-mediated gene inhibition
dCas9: catalytically inactive Cas9
dCas9^p300 Core^: dCas9 fused to the catalytic core of the human histone acetyltransferase p300
DISCOVER-seq: Discovery of In Situ Cas Off-targets and VERification by sequencing
DMD: Duchenne muscular dystrophy
DNMT3A/B: DNA methyltransferase
fCas9: Cas9 fusion with the FokI nuclease
FDA: U.S. Food and Drug Administration
FnCas9: *Francisella novicida* Cas9
GvHD: graft-versus-host disease
HBB: β-globin gene
HDR: homology-directed recombination
HLA: human leukocyte antigen
HPFH: hereditary persistence of fetal hemoglobin
KRAB: Krüppel-associated box domain of Kox1
LCA10: Leber congenital amaurosis type 10
LNP: lipid nanoparticle
LSD1: lysine-specific histone demethylase
LshCas13a, previously known as LshC2c2: *Leptotrichia shahii* Cas13a
LwaCas13a: *Leptotrichia wadei* Cas13a
M.sssI: *Mollicutes spiroplasma* strain MQ1
NmCas9: *Neisseria meningitidis* Cas9
PACE: phage-assisted continuous evolution
PAMmer: ssDNA oligonucleotide with a PAM sequence
PD-1: programmed cell death protein 1
PD-L1: PD-1 ligand
PID: primary immunodeficiency
PspCas13b: *Prevotella sp.* Cas13b
RNP: ribonucleoprotein
SaCas9: *Staphylococcus aureus* Cas9
SAM: Synergistic Activation Mediator
scFv: single-chain variable fragment
SCID-X1: X-linked severe combined immunodeficiency
SpCas9: *Streptococcus pyogenes* Cas9
TALE: transcription activator-like effector protein
TALEN: transcription activator-like effector nuclease
TCR: T-cell receptor
TET1CD: catalytic domain of TET1
TIL: tumor-infiltrating lymphocyte
TRAC: TCR α constant
TRBC: TCR β constant
UDiTaS: uni-directional targeted sequencing
WAS: Wiskott–Aldrich syndrome
ZFN: zinc-finger nuclease
ZFP: zinc finger protein

## References

[1] Lander E.S., Linton L.M., Birren B., Nusbaum C., Zody M.C., Baldwin J. et al. (2001) Initial sequencing and analysis of the human genome. Nature 409, 860–921 11237011 10.1038/35057062

[2] International HapMap Consortium (2003) The International HapMap Project. Nature 426, 789–796 10.1038/nature02168 14685227

[3] Sayers E.W., Agarwala R., Bolton E.E., Brister J.R., Canese K., Clark K. et al. (2019) Database resources of the National Center for Biotechnology Information. Nucleic Acids Res. 47, D23–D28 10.1093/nar/gky1069 30395293 PMC6323993

[4] Wellcome Trust Case Control Consortium (2007) Genome-wide association study of 14,000 cases of seven common diseases and 3,000 shared controls. Nature 447, 661–678 10.1038/nature05911 17554300 PMC2719288

[5] Visscher P.M., Wray N.R., Zhang Q., Sklar P., McCarthy M.I., Brown M.A. et al. (2017) 10 years of GWAS discovery: biology, function, and translation. Am. J. Hum. Genet. 101, 5–22 10.1016/j.ajhg.2017.06.005 28686856 PMC5501872

[6] Gaj T., Gersbach C.A. and Barbas C.F. III (2013) ZFN, TALEN, and CRISPR/Cas-based methods for genome engineering. Trends Biotechnol. 31, 397–405 10.1016/j.tibtech.2013.04.004 23664777 PMC3694601

[7] Carroll D. (2011) Genome engineering with zinc-finger nucleases. Genetics 188, 773–782 10.1534/genetics.111.131433 21828278 PMC3176093

[8] Joung J.K. and Sander J.D. (2013) TALENs: a widely applicable technology for targeted genome editing. Nat. Rev. Mol. Cell Biol. 14, 49–55 10.1038/nrm3486 23169466 PMC3547402

[9] Mojica F.J., Diez-Villasenor C., Soria E. and Juez G. (2000) Biological significance of a family of regularly spaced repeats in the genomes of Archaea, Bacteria and mitochondria. Mol. Microbiol. 36, 244–246 10.1046/j.1365-2958.2000.01838.x 10760181

[10] Barrangou R., Fremaux C., Deveau H., Richards M., Boyaval P., Moineau S. et al. (2007) CRISPR provides acquired resistance against viruses in prokaryotes. Science 315, 1709–1712 10.1126/science.1138140 17379808

[11] Adli M. (2018) The CRISPR tool kit for genome editing and beyond. Nat. Commun. 9, 1911 10.1038/s41467-018-04252-2 29765029 PMC5953931

[12] Sternberg S.H. and Doudna J.A. (2015) Expanding the biologist’s toolkit with CRISPR-Cas9. Mol. Cell 58, 568–574 10.1016/j.molcel.2015.02.032 26000842

[13] Mali P., Yang L., Esvelt K.M., Aach J., Guell M., DiCarlo J.E. et al. (2013) RNA-guided human genome engineering via Cas9. Science 339, 823–826 10.1126/science.1232033 23287722 PMC3712628

[14] Cong L., Ran F.A., Cox D., Lin S., Barretto R., Habib N. et al. (2013) Multiplex genome engineering using CRISPR/Cas systems. Science 339, 819–823 10.1126/science.1231143 23287718 PMC3795411

[15] Wiedenheft B., Sternberg S.H. and Doudna J.A. (2012) RNA-guided genetic silencing systems in bacteria and archaea. Nature 482, 331–338 10.1038/nature10886 22337052

[16] Jinek M., Chylinski K., Fonfara I., Hauer M., Doudna J.A. and Charpentier E. (2012) A programmable dual-RNA-guided DNA endonuclease in adaptive bacterial immunity. Science 337, 816–821 10.1126/science.1225829 22745249 PMC6286148

[17] Lino C.A., Harper J.C., Carney J.P. and Timlin J.A. (2018) Delivering CRISPR: a review of the challenges and approaches. Drug Deliv. 25, 1234–1257 10.1080/10717544.2018.1474964 29801422 PMC6058482

[18] Hütter G., Nowak D., Mossner M., Ganepola S., Müßig A., Allers K. et al. (2009) Long-term control of HIV by CCR5 Delta32/Delta32 stem-cell transplantation. N. Engl. J. Med. 360, 692–698 10.1056/NEJMoa0802905 19213682

[19] Yukl S.A., Boritz E., Busch M., Bentsen C., Chun T.W., Douek D. et al. (2013) Challenges in detecting HIV persistence during potentially curative interventions: a study of the Berlin Patient. PLoS Pathog. 9, e1003347 10.1371/journal.ppat.1003347 23671416 PMC3649997

[20] Perez E.E., Wang J., Miller J.C., Jouvenot Y., Kim K.A., Liu O. et al. (2008) Establishment of HIV-1 resistance in CD4+ T cells by genome editing using zinc-finger nucleases. Nat. Biotechnol. 26, 808–816 10.1038/nbt1410 18587387 PMC3422503

[21] Tebas P., Stein D., Tang W.W., Frank I., Wang S.Q., Lee G. et al. (2014) Gene Editing of CCR5 in Autologous CD4 T Cells of Persons Infected with HIV. N. Engl. J. Med. 370, 901–910 10.1056/NEJMoa1300662 24597865 PMC4084652

[22] Zhang Y., Qin W., Lu X., Xu J., Huang H., Bai H. et al. (2017) Programmable base editing of zebrafish genome using a modified CRISPR-Cas9 system. Nat. Commun. 8, 118 10.1038/s41467-017-00175-6 28740134 PMC5524635

[23] Ye L., Wang J., Beyer A.I., Teque F., Cradick T.J., Qi Z. et al. (2014) Seamless modification of wild-type induced pluripotent stem cells to the natural CCR5Δ32 mutation confers resistance to HIV infection. Proc. Natl Acad. Sci. U.S.A. 111, 9591–9596 10.1073/pnas.1407473111 24927590 PMC4084478

[24] Allen A.G., Chung C.H., Atkins A., Dampier W., Khalili K., Nonnemacher M.R. et al. (2018) Gene editing of HIV-1 co-receptors to prevent and/or cure virus infection. Front. Microbiol. 9, 2940 10.3389/fmicb.2018.02940 30619107 PMC6304358

[25] Khalili K., White M.K. and Jacobson J.M. (2017) Novel AIDS therapies based on gene editing. Cell. Mol. Life Sci. 74, 2439–2450 10.1007/s00018-017-2479-z 28210784 PMC5474186

[26] Didigu C.A., Wilen C.B., Wang J., Doung J., Secreto A.J., Danet-Desnoyers G.A. et al. (2014) Simultaneous zinc-finger nuclease editing of the HIV coreceptors ccr5 and cxcr4 protects CD41 T cells from HIV-1 infection. Blood 123, 61–69 10.1182/blood-2013-08-521229 24162716 PMC3879906

[27] Liu Z., Chen S., Jin X., Wang Q., Yang K., Li C. et al. (2017) Genome editing of the HIV co-receptors CCR5 and CXCR4 by CRISPR-Cas9 protects CD4+ T cells from HIV-1 infection. Cell Biosci. 7, 47 10.1186/s13578-017-0174-2 28904745 PMC5591563

[28] Yu S., Yao Y., Xiao H., Li J., Liu Q., Yang Y. et al. (2018) Simultaneous knockout of CXCR4 and CCR5 genes in CD4+ T cells via CRISPR/Cas9 confers resistance to both X4- and R5-tropic human immunodeficiency virus type 1 infection. Hum. Gene Ther. 29, 51–67 10.1089/hum.2017.032 28599597

[29] Kaminski R., Bella R., Yin C., Otte J., Ferrante P., Gendelman H.E. et al. (2016) Excision of HIV-1 DNA by gene editing: a proof-of-concept *in vivo* study. Gene Ther. 23, 690–695 10.1038/gt.2016.41 27194423 PMC4974122

[30] Dash P.K., Kaminski R., Bella R., Su H., Mathews S., Ahooyi T.M. et al. (2019) Sequential LASER ART and CRISPR treatments eliminate HIV-1 in a subset of infected humanized mice. Nat. Commun. 10, 2753 10.1038/s41467-019-10366-y 31266936 PMC6606613

[31] Cyranoski D. (2016) First trial of CRISPR in people. Nature 535, 479 10.1038/nature.2016.20988 27466108

[32] Naidoo J., Page D.B., Li B.T., Connell L.C., Schindler K., Lacouture M.E. et al. (2015) Toxicities of the anti-PD-1 and anti-PD-L1 immune checkpoint antibodies. Ann. Oncol. 26, 2375–2391 10.1093/annonc/mdv383 26371282 PMC6267867

[33] Gong J., Chehrazi-Raffle A., Reddi S. and Salgia R. (2018) Development of PD-1 and PD-L1 inhibitors as a form of cancer immunotherapy: a comprehensive review of registration trials and future considerations. J. Immunother. Cancer 6, 8 10.1186/s40425-018-0316-z 29357948 PMC5778665

[34] Lu Y., Huang M., Deng T., Zhou X., Yu K., Liang M. et al. (2018) A phase I trial of PD-1 deficient engineered T cells with CRISPR/Cas9 in patients with advanced non-small cell lung cancer with PD-L1 expression [abstract]. Proceedings of the American Association for Cancer Research Annual Meeting 2018. Cancer Res. 78, Chicago, IL; Philadelphia (PA), Abstract nr CT133

[35] Lu Y., Xue J., Deng T., Zhou X., Yu K., Huang M. et al. (2018) A phase I trial of PD-1 deficient engineered T cells with CRISPR/Cas9 in patients with advanced non-small cell lung cancer. J. Clin. Oncol. 36, 3050 10.1200/JCO.2018.36.15_suppl.3050

[36] Rohaan M.W., van den Berg J.H., Kvistborg P. and Haanen JBAG (2018) Adoptive transfer of tumor-infiltrating lymphocytes in melanoma: a viable treatment option. J. Immunother. Cancer 6, 102 10.1186/s40425-018-0391-1 30285902 PMC6171186

[37] Geukes Foppen M.H., Donia M., Svane I.M. and Haanen JBAG (2015) Tumor-infiltrating lymphocytes for the treatment of metastatic cancer. Mol. Oncol. 9, 1918–1935 10.1016/j.molonc.2015.10.018 26578452 PMC5528735

[38] Tran E., Ahmadzadeh M., Lu Y.C., Gros A., Turcotte S., Robbins P.F. et al. (2015) Immunogenicity of somatic mutations in human gastrointestinal cancers. Science 350, 1387–1390 10.1126/science.aad1253 26516200 PMC7445892

[39] Palmer D.C., Guittard G.C., Franco Z., Crompton J.G., Eil R.L., Patel S.J. et al. (2015) Cish actively silences TCR signaling in CD8+ T cells to maintain tumor tolerance. J. Exp. Med. 212, 2095–2113 10.1084/jem.20150304 26527801 PMC4647263

[40] Beane J.D., Lee G., Zheng Z., Mendel M., Abate-Daga D., Bharathan M. et al. (2015) Clinical scale zinc finger nuclease-mediated gene editing of PD-1 in tumor infiltrating lymphocytes for the treatment of metastatic melanoma. Mol. Ther. 23, 1380–1390 10.1038/mt.2015.71 25939491 PMC4817870

[41] Barrett D.M., Grupp S.A. and June C.H. (2015) Chimeric antigen receptor- and TCR-modified T cells enter main street and wall street. J. Immunol. 195, 755–761 10.4049/jimmunol.1500751 26188068 PMC4507286

[42] Fesnak A.D., June C.H. and Levine B.L. (2016) Engineered T cells: the promise and challenges of cancer immunotherapy. Nat. Rev. Cancer 16, 566–581 10.1038/nrc.2016.97 27550819 PMC5543811

[43] June C.H., O’Connor R.S., Kawalekar O.U., Ghassemi S. and Milone M.C. (2018) CAR T cell immunotherapy for human cancer. Science 359, 1361–1365 10.1126/science.aar6711 29567707

[44] Garrido F., Aptsiauri N., Doorduijn E.M., Garcia Lora A.M. and van Hall T. (2016) The urgent need to recover MHC class I in cancers for effective immunotherapy. Curr. Opin. Immunol. 39, 44–51 10.1016/j.coi.2015.12.007 26796069 PMC5138279

[45] Karpanen T. and Olweis J. (2015) T-cell receptor gene therapy e ready to go viral? Mol. Oncol. 9, 2019–2042 10.1016/j.molonc.2015.10.006 26548533 PMC5528733

[46] Thomas R., Al-Khadairi G., Roeland J., Hendrickx W., Dermime A., Bedognetti D. et al. (2018) NY-ESO-1 based immunotherapy of cancer: current perspectives. Front. Immunol. 9, 947 10.3389/fimmu.2018.00947 29770138 PMC5941317

[47] Milone M.C. and Bhoj V.G. (2018) The pharmacology of T cell therapies. Mol. Ther. Methods Clin. Dev. 8, 210–221 29552577 10.1016/j.omtm.2018.01.010 PMC5852291

[48] Zhao J., Song Y. and Liu D. (2019) Clinical trials of dual-targeted CAR T cells, donor-derived CAR T cells, and universal CAR T cells for acute lumphoid leukemia. J. Hematol. Oncol. 12, 17 10.1186/s13045-019-0705-x 30764841 PMC6376657

[49] Zheng K., Wang Y., Li N., Jiang F.F., Wu C.X., Liu F. et al. (2018) Highly efficient base editing in bacteria using a Cas9-cytidine deaminase fusion. Commun. Biol. 1, 32 10.1038/s42003-018-0035-5 30271918 PMC6123677

[50] Jin C., Fotaki G., Ramachandran M., Nilsson B., Essand M. and Yu D. (2016) Safe engineering of CAR T cells for adoptive cell therapy of cancer using long-term episomal gene transfer. EMBO Mol. Med. 8, 702–711 10.15252/emmm.201505869 27189167 PMC4931286

[51] Hacein-Bey-Abina S., Garrigue A., Wang G.P., Soulier J., Lim A., Morillon E. et al. (2008) Insertional oncogenesis in 4 patients after retrovirus-mediated gene therapy of SCID-X1. J. Clin. Invest. 118, 3132–3142 10.1172/JCI35700 18688285 PMC2496963

[52] Hacein-Bey-Abina S., Von Kalle C., Schmidt M., McCormack M.P., Wulfraat N., Leboulch P. et al. (2003) LMO2-associated clonal T cell proliferation in two patients after gene therapy for SCID-X1. Science 302, 415–419 10.1126/science.1088547 14564000

[53] Avedillo Díez I., Zychlinski D., Coci E.G., Galla M., Modlich U., Dewey R.A. et al. (2011) Development of novel efficient SIN vectors with improved safety features for Wiskott Aldrich syndrome stem cell based gene therapy. Mol. Pharm. 8, 1525–1537 10.1021/mp200132u 21851067

[54] Ott M.G., Schmidt M., Schwarzwaelder K., Stein S., Siler U., Koehl Y. et al. (2006) Correction of X-linked chronic granulomatous disease by gene therapy, augmented by insertional activation of MDS1-EVI1, PRDM16 or SETBP1. Nat. Med. 12, 401–409 10.1038/nm1393 16582916

[55] Modlich U., Navarro S., Zychlinski D., Maetzig T., Knoess S., Brugman M.H. et al. (2009) Insertional transformation of hematopoietic cells by self-inactivating lentiviral and gammaretroviral vectors. Mol. Ther. 17, 1919–1928 10.1038/mt.2009.179 19672245 PMC2835038

[56] Osborn M.J., Webber B.R., Knipping F., Lonetree C., Tennis N., DeFeo A.P. et al. (2016) Evaluation of TCR gene editing achieved by TALENs, CRISPR/Cas9, and megaTAL Nucleases. Mol. Ther. 24, 570–581 10.1038/mt.2015.197 26502778 PMC4786913

[57] Eyquem J., Mansilla-Soto J., Giavridis T., van der Stegen S.J.C., Hamieh M., Cunanan K.M. et al. (2017) Targeting a CAR to the TRAC locus with CRISPR/Cas9 enhances tumour rejection. Nature 543, 113–117 10.1038/nature21405 28225754 PMC5558614

[58] Qasim W., Zhan H., Samarasinghe S., Adams S., Amrolia P., Stafford S. et al. (2017) Molecular remission of infant B-ALL after infusion of universal TALEN gene-edited CAR T cells. Sci. Transl. Med. 9, eaaj2013 10.1126/scitranslmed.aaj2013 28123068

[59] Roth T.L., Puig-Saus C., Yu R., Shifrut E., Carnevale J., Li P.J. et al. (2018) Reprogramming human T cell function and specificity with non-viral genome targeting. Nature 559, 405–409 10.1038/s41586-018-0326-5 29995861 PMC6239417

[60] Smith M., Zakrzewski J., James S. and Sadelain M. (2018) Posttransplant chimeric antigen receptor therapy. Blood 131, 1045–1052 10.1182/blood-2017-08-752121 29358181 PMC5865610

[61] Gaudelli N.M., Komor A.C., Rees H.A., Packer M.S., Badran A.H., Bryson D.I. et al. (2017) Programmable base editing of A*T to G*C in genomic DNA without DNA cleavage. Nature 551, 464–471 10.1038/nature24644 29160308 PMC5726555

[62] Komor A.C., Zhao K.T., Packer M.S., Gaudelli N.M., Waterbury A.L., Koblan L.W. et al. (2017) Improved base excision repair inhibition and bacteriophage Mu Gam protein yields C: G-to-T: a base editors with higher efficiency and product purity. Sci Adv 3, eaao4774 10.1126/sciadv.aao4774 28875174 PMC5576876

[63] Ren J., Liu X., Fang C., Jiang S., June C.H. and Zhao Y. (2016) Multiplex genome editing to generate universal CAR T cells resistant to PD1 inhibition. Clin. Cancer Res. 23, 2255–2266 10.1158/1078-0432.CCR-16-1300 27815355 PMC5413401

[64] Poirot L., Philip B., Schiffer-Mannioui C., Le Clerre D., Chion-Sotinel I., Dernaime S. et al. (2015) Multiplex genome-edited T-cell manufacturing platform for “off-the-shelf” adoptive T-cell immunotherapies. Cancer Res. 75, 3853–3864 10.1158/0008-5472.CAN-14-3321 26183927

[65] Gomes-Silva D., Srinivasan M., Sharma S., Lee C.M., Wagner D.L., Davis T.H. et al. (2017) CD7-edited T cells expressing a CD7-specific CAR for the therapy of T-cell malignancies. Blood 130, 285–296 10.1182/blood-2017-01-761320 28539325 PMC5520470

[66] Haanen J., Carbonnel F., Robert C., Kerr K.M., Peters S., Larkin J. et al. (2017) Management of toxicities from immunotherapy: ESMO Clinical Practice Guidelines for diagnosis, treatment and follow-up. Ann. Oncol. 28, iv119–iv142 10.1093/annonc/mdx225 28881921

[67] Chong E.A., Melenhorst J.J., Lacey S.F., Ambrose D.E., Gonzalez V., Levine B.L. et al. (2017) PD-1 blockade modulates chimeric antigen receptor (CAR)-modified T cells: refueling the CAR. Blood 129, 1039–1041 10.1182/blood-2016-09-738245 28031179 PMC5391777

[68] Locke F.L., Westin J.R., Miklos D.B., Herrara A.F., Jacobson C.A., Lee J. et al. (2017) Phase 1 results from ZUMA-6: axicabtagene ciloleucel (axi-cel; KTE-C19) in combination with atezolizumab for the treatment of patients with refractory diffuse large B cell lymphoma (DLBCL). Blood 130, 2826

[69] Jacobson C.A., Locke F.L., Miklos D.B., Herrara A.F., Westin J.R., Lee J. et al. (2019) End of phase 1 results from Zuma-6: axicabtagene ciloleucel (Axi-Cel) in combination with atezolizumab for the treatment of patients with refractory diffuse large B cell lymphoma. Biol. Blood Marrow Transplant. 25, S173 10.1016/j.bbmt.2018.12.314

[70] Hu W., Zi Z., Jin Y., Li G., Shao K., Cai Q. et al. (2019) CRISPR/Cas9-mediated PD-1 disruption enhances human mesothelintargeted CAR T cell effector functions. Cancer Immunol. Immunother. 68, 365077 10.1007/s00262-018-2281-2 PMC11028344 30523370

[71] Rupp L.J., Schumann K., Roybal K.T., Gate R.E., Ye C.J., Lim W.A. et al. (2017) CRISPR/Cas9-mediated PD-1 disruption enhances anti-tumor efficacy of human chimeric antigen receptor T cells. Sci. Rep. 7, 737 10.1038/s41598-017-00462-8 28389661 PMC5428439

[72] Cavazzana-Calvo M., Payen E., Negre O., Wang G., Hehir K., Fusil F. et al. (2010) Transfusion independence and HMGA2 activation after gene therapy of human b-thalassaemia. Nature 467, 318–322 10.1038/nature09328 20844535 PMC3355472

[73] Milone M.C. and O'Doherty U. (2018) Clinical use of lentiviral vectors. Leukemia 32, 1529–1541 10.1038/s41375-018-0106-0 29654266 PMC6035154

[74] Cai L., Bai H., Mahairaki V., Gao Y., He C., Wen Y. et al. (2018) A universal approach to correct various HBB gene mutations in human stem cells for gene therapy of beta-thalassemia and sickle cell disease. Stem Cells Transl. Med. 7, 87–97 10.1002/sctm.17-0066 29164808 PMC5746148

[75] Ou Z., Niu X., He W., Chen Y., Song B., Xian Y. et al. (2016) The combination of CRISPR/Cas9 and iPSC technologies in the gene therapy of human β-thalassemia in mice. Sci. Rep. 6, 32463 10.1038/srep32463 27581487 PMC5007518

[76] Wattanapanitch M., Damkham N., Potirat P., Trakarnsanga K., Janan M., U-pratya Y. et al. (2018) One-step genetic correction of hemoglobin E/beta-thalassemia patient-derived iPSCs by the CRISPR/Cas9 system. Stem Cell Res. Ther. 9, 46 29482624 10.1186/s13287-018-0779-3 PMC5828150

[77] Xie F., Ye L., Chang J.C., Beyer A.I., Wang J., Muench M.O. et al. (2014) Seamless gene correction of b-thalassemia mutations in patient-specific iPSCs using CRISPR/Cas9 and piggyBac. Genome Res. 24, 1526–1533 10.1101/gr.173427.114 25096406 PMC4158758

[78] Patsali P., Turchiano G., Papasavva P., Romito M., Loucari C.C., Stephanou C. et al. (2019) Correction of IVS I-110 (G>A) b-thalassemia by CRISPR/Cas- and TALEN-mediated disruption of aberrant progenitor cells. Haematologica 104, e497–e501 10.3324/haematol.2018.215178 31004018 PMC6821606

[79] Wienert B., Martyn G.E., Funnell A.P.W., Quinlan K.G.R. and Crossley M. (2018) Wake-up sleepy gene: reactivating fetal globin for β-hemoglobinopathies. Trends Genet. 34, 927–940 10.1016/j.tig.2018.09.004 30287096

[80] Ye L., Wang J., Tan Y., Beyer A.I., Xie F., Muench M.O. et al. (2016) Genome editing using CRISPR-Cas9 to create the HPFH genotype in HSPCs: An approach for treating sickle cell disease and β-thalassemia. Proc. Natl. Acad. Sci. U.S.A. 113, 10661–10665 10.1073/pnas.1612075113 27601644 PMC5035856

[81] Bjurström C.F., Mojadidi M., Phillips J., Kuo C., Lai S., Lill G.R. et al. (2016) Reactivating fetal hemoglobin expression in human adult erythroblasts through BCL11A knockdown using targeted endonucleases. Mol. Ther. Nucleic Acids 5, e351 10.1038/mtna.2016.52 28131278 PMC5023398

[82] Chang K.H., Smith S.E., Sullivan T., Chen K., Zhou Q., West J.A. et al. (2017) Long-term engraftment and fetal globin induction upon BCL11A gene editing inbone-marrow-derived CD34+ hematopoietic stem and progenitor cells. Mol. Ther. Methods Clin. Dev. 4, 137–148 28344999 10.1016/j.omtm.2016.12.009 PMC5363298

[83] Zhen S. and Li X. (2017) Oncogenic human papillomavirus: application of CRISPR/Cas9 therapeutic strategies for cervical cancer. Cell. Physiol. Biochem. 44, 2455–2466 10.1159/000486168 29268281

[84] Ren C., Li X., Mao L., Xiong J., Gao C., Shen H. et al. (2019) An effective and biocompatible polyethylenimine based vaginal suppository for gene delivery. Nanomedicine 20, 101994 10.1016/j.nano.2019.03.016 31028885

[85] Ding W., Hu Z., Zhu D., Jiang X., Yu L., Wang X. et al. (2014) Zinc finger nucleases targeting the human papillomavirus E7 oncogene induce E7 disruption and a transformed phenotype in HPV16/18-positive cervical cancer cells. Clin. Cancer Res. 20, 6495–6503 10.1158/1078-0432.CCR-14-0250 25336692

[86] Hu Z., Ding W., Zhu D., Yu L., Jiang X., Wang X. et al. (2015) TALEN-mediated targeting of HPV oncogenes ameliorates HPV-related cervical malignancy. J. Clin. Invest. 125, 425–436 10.1172/JCI78206 25500889 PMC4382249

[87] Lao Y.H., Li M., Gao M.A., Shao D., Chi C.W., Huang D. et al. (2018) HPV oncogene manipulation using nonvirally delivered CRISPR/Cas9 or Natronobacterium gregoryi argonaute. Adv. Sci. 5, 1700540 10.1002/advs.201700540 30027026 PMC6051382

[88] Hsu D.S., Kornepati A.V.R., Glover W., Kennedy E.M. and Cullen B.R. (2018) Targeting HPV16 DNA using CRISPR/Cas inhibits anal cancer growth *in vivo*. Future Virol. 13, 475–482 10.2217/fvl-2018-0010 30245733 PMC6136077

[89] Yoshiba T., Saga Y., Urabe M., Uchibori R., Matsubara S., Fujiwara H. et al. (2019) CRISPR/Cas9-mediated cervical cancer treatment targeting human papillomavirus E6. Oncol. Lett. 17, 2197–2206 30675284 10.3892/ol.2018.9815 PMC6341785

[90] Li H., Haurigot V., Doyon Y., Li T., Wong S.Y., Bhagwat A.S. et al. (2011) *In vivo* genome editing restores haemostasis in a mouse model of haemophilia. Nature 475, 217–221 10.1038/nature10177 21706032 PMC3152293

[91] Anguela X.M., Sharma R., Doyon Y., Miller J.C., Li H., Haurigot V. et al. (2013) Robust ZFN-mediated genome editing in adult hemophilic mice. Blood 122, 3283–3287 10.1182/blood-2013-04-497354 24085764 PMC3821724

[92] Sharma R., Anguela X.M., Doyon Y., Wechsler T., DeKelver R.C., Sproul S. et al. (2015) In vivo genome editing of the albumin locus as a platform for protein replacement therapy. Blood 126, 1777–1784 10.1182/blood-2014-12-615492 26297739 PMC4600017

[93] Zong Y., Wang Y., Li C., Zhang R., Chen K., Ran Y. et al. (2017) Precise base editing in rice, wheat and maize with a Cas9-cytidine deaminase fusion. Nat. Biotechnol. 35, 438–440 10.1038/nbt.3811 28244994

[94] Sivalingam J., Kenanov D., Han H., Nirmal A.J., Ng W.H., Lee S.S. et al. (2016) Multidimensional genome-wide analyses show accurate FVIII integration by ZFN in primary human cells. Mol. Ther. 24, 607–619 10.1038/mt.2015.223 26689265 PMC4786920

[95] Ou L., DeKelver R.C., Rohde M., Tom S., Radeke R., St. Martin S.J. et al. (2019) ZFN-mediated in vivo genome editing corrects murine Hurler Syndrome. Mol. Ther. 27, 178–187 10.1016/j.ymthe.2018.10.018 30528089 PMC6319315

[96] Laoharawee K., DeKelver R.C., Podetz-Pedersen K.M., Rohde M., Sproul S., Nguyen H.O. et al. (2018) Dose-dependent prevention of metabolic and neurologic disease in murine MPS II by ZFN-mediated *in vivo* genome editing. Mol. Ther. 26, 1127–1136 10.1016/j.ymthe.2018.03.002 29580682 PMC6080131

[97] Sheridan C. (2018) Sangamo’s landmark genome editing trial gets mixed reception. Nat. Biotechnol. 36, 907–908 10.1038/nbt1018-907 30307929

[98] Maeder M.L., Stefanidakis M., Wilson C.J., Bara R., Barrera L.A., Bounoutas G.S. et al. (2019) Development of a gene-editing approach to restore vision loss in Leber congenital amaurosis type 10. Nat. Med. 25, 229–233 10.1038/s41591-018-0327-9 30664785

[99] Dominguez A.A., Lim W.A. and Qi L.S. (2016) Beyond editing: repurposing CRISPR–Cas9 for precision genome regulation and interrogation. Nat. Rev. Mol. Cell Biol. 17, 5–15 10.1038/nrm.2015.2 26670017 PMC4922510

[100] Thakore P.I., Black J.B., Hilton I.B. and Gersbach C.A. (2016) Editing the epigenome: technologies for programmable transcriptional modulation and epigenetic regulation. Nat. Methods 13, 127–137 10.1038/nmeth.3733 26820547 PMC4922638

[101] Hong A. (2018) CRISPR in personalized medicine: industry perspectives in gene editing. Semin. Perinatol. 42, 501–507 30376985 10.1053/j.semperi.2018.09.008

[102] Booth C., Gaspar H.B. and Thrasher A.J. (2016) Treating immunodeficiency through HSC Gene iherapy. Trends Mol. Med. 22, 317–327 10.1016/j.molmed.2016.02.002 26993219

[103] Genovese P., Schiroli G., Escobar G., Di Tomaso T., Firrito C., Calabria A. et al. (2014) Targeted genome editing in human repopulating haematopoietic stem cells. Nature 510, 235–240 10.1038/nature13420 24870228 PMC4082311

[104] Pavel-Dinu M., Wiebking V., Denjene B.T., Srifa W., Mantri S., Nicolas C.E. et al. (2019) Gene correction for SCID-X1 in long-term hematopoietic stem cells. Nat. Commun. 10, 1634, 10.1038/s41467-019-13620-5 PMC6456568 30967552

[105] Schiroli G., Ferrari S., Conway A., Jacob A., Capo V., Albano L. et al. (2017) Preclinical modeling highlights the therapeutic potential of hematopoietic stem cell gene editing for correction of SCID-X1. Sci. Transl. Med. 9, eean0820 10.1126/scitranslmed.aan0820 29021165

[106] De Ravin S.S., Li L., Wu X., Choi U., Allen C., Koontz S. et al. (2017) CRISPR-Cas9 gene repair of hematopoietic stem cells from patients with X-linked chronic granulomatous disease. Sci. Transl. Med. 9, eaah3480 10.1126/scitranslmed.aah3480 28077679

[107] De Ravin S.S., Reik A., Liu P.Q., Li L., Wu X., Su L. et al. (2016) Targeted gene addition in human CD34+ hematopoietic cells for correction of X-linked chronic granulomatous disease. Nat. Biotechnol. 34, 424–429 10.1038/nbt.3513 26950749 PMC4824656

[108] Dreyer A.K., Hoffmann D., Lachmann N., Ackermann M., Steinemann D., Timm B. et al. (2015) TALEN-mediated functional correction of X-linked chronic granulomatous disease in patient-derived induced pluripotent stem cells. Biomaterials 69, 191–200 10.1016/j.biomaterials.2015.07.057 26295532

[109] Flynn R., Grundmann A., Renz P., Hänseler W., Cowley S.A. and Moore M.D. (2015) CRISPR-mediated genotypic and phenotypic correction of a chronic granulomatous disease mutation in human iPS cells. Exp. Hematol. 43, 838–848 10.1016/j.exphem.2015.06.002 26101162 PMC4596252

[110] Merling R.K., Sweeney C.L., Chu J., Bodansky A., Choi U., Long Priel D. et al. (2015) An AAVS1-targeted minigene platform for correction of iPSCs from all five types of chronic granulomatous disease. Mol. Ther. 23, 147–157 10.1038/mt.2014.195 25288370 PMC4426805

[111] Sürün D., Schwäble J., Tomasovic A., Ehling R., Stein S., Kurrle N. et al. (2018) High efficiency gene correction in hematopoietic cells by donor-template-free CRISPR/Cas9 genome editing. Mol. Ther. Nucleic Acids 10, 1–8 29499925 10.1016/j.omtn.2017.11.001 PMC5723376

[112] Gutierrez-Guerrero A., Sanchez-Hernandez S., Galvani G., Pinedo-Gomez J., Martin-Guerra R., Sanchez-Gilabert A. et al. (2018) Comparison of zinc finger nucleases versus CRISPR-specific nucleases for genome editing of the Wiskott-Aldrich Syndrome locus. Hum. Gene Ther. 29, 366–380 10.1089/hum.2017.047 28922955

[113] Rodrigues G.A., Shalaev E., Karami T.K., Cunningham J., Slater N.K.H. and Rivers H.M. (2018) Pharmaceutical development of AAV-based gene therapy products for the eye. Pharm. Res. 36, 29 10.1007/s11095-018-2554-7 30591984 PMC6308217

[114] Trapani I. and Auricchio A. (2018) Seeing the light after 25 years of retinal gene therapy. Trends Mol. Med. 24, 669–681 10.1016/j.molmed.2018.06.006 29983335

[115] McCullough K.T., Boye S.L., Fajardo D., Calabro K., Peterson J.J., Strang C.E. et al. (2019) Somatic gene editing of GUCY2D by AAV-CRISPR/Cas9 alters retinal structure and function in mouse and macaque. Hum. Gene Ther. 30, 571–589 10.1089/hum.2018.193 30358434 PMC6534089

[116] Xu C., Cho G.Y., Sengillo J.D., Park K.S., Mahajan V.B. and Tsang S.H. (2018) Translation of CRISPR genome surgery to the bedside for retinal diseases. Front. Cell Dev. Biol. 6, 46 10.3389/fcell.2018.00046 29876348 PMC5974543

[117] Yu W. and Wu Z. (2018) *In vivo* applications of CRISPR-based genome editing in the retina. Front. Cell Dev. Biol. 6, 53 10.3389/fcell.2018.00053 29868583 PMC5960719

[118] Fuster-García C., García-García G., González-Romero E., Jaijo T., Sequedo M.D., Ayuso C. et al. (2017) USH2A gene editing using the CRISPR system. Mol. Ther. Nucleic Acids 8, 529–541 28918053 10.1016/j.omtn.2017.08.003 PMC5573797

[119] Mianné J., Chessum L., Kumar S., Aguilar C., Codner G., Hutchison M. et al. (2016) Correction of the auditory phenotype in C57BL/6N mice via CRISPR/Cas9-mediated homology directed repair. Genome Med. 8, 16 10.1186/s13073-016-0273-4 26876963 PMC4753642

[120] Gao X., Tao Y., Lamas V., Huang M., Yeh W.H., Pan B. et al. (2018) Treatment of autosomal dominant hearing loss by in vivo delivery of genome editing agents. Nature 553, 217–221 10.1038/nature25164 29258297 PMC5784267

[121] Zuris J.A., Thompson D.B., Shu Y., Guilinger J.P., Bessen J.L., Hu J.H. et al. (2015) Cationic lipid-mediated delivery of proteins enables efficient protein-based genome editing *in vitro* and *in vivo*. Nat. Biotechnol. 33, 73–80 10.1038/nbt.3081 25357182 PMC4289409

[122] Stephens C.J., Kashentseva E., Everett W., Kaliberova L. and Curiel D.T. (2018) Targeted *in vivo* knock-in of human alpha-1-antitrypsin cDNA using adenoviral delivery of CRISPR/Cas9. Gene Ther. 25, 139–156 10.1038/s41434-018-0003-1 29588497 PMC5919923

[123] Bjursell M., Porritt M.J., Ericson E., Taheri-Ghahfarokhi A., Clausen M., Magnusson L. et al. (2018) Therapeutic genome editing with CRISPR/Cas9 in a humanized mouse model ameliorates α1-antitrypsin deficiency phenotype. EBioMedicine 29, 104–111 10.1016/j.ebiom.2018.02.015 29500128 PMC5925576

[124] Shen S., Sanchez M.E., Blomenkamp K., Corcoran E.M., Marco E., Yudkoff C.J. et al. (2018) Amelioration of alpha-1 antitrypsin deficiency diseases with genome editing in transgenic mice. Hum. Gene Ther. 29, 861–873 10.1089/hum.2017.227 29641323

[125] Song C.Q., Wang D., Jiang T., O’Connor K., Tang Q., Cai L. et al. (2018) *In vivo* genome editing partially restores alpha 1-antitrypsin in a murine model of AAT deficiency. Hum. Gene Ther. 29, 853–860 10.1089/hum.2017.225 29597895 PMC6110121

[126] Finn J.D., Smith A.R., Patel M.C., Shaw L., Youniss M.R., van Heteren J. et al. (2018) A single administration of CRISPR/Cas9 lipid nanoparticles achieves robust and persistent *in vivo* genome editing. Cell Rep. 22, 2227–2235 10.1016/j.celrep.2018.02.014 29490262

[127] Yang H.C. and Chen P.J. (2018) The potential and challenges of CRISPR-Cas in eradication of hepatitis B virus covalently closed circular DNA. Virus Res. 244, 304–310 10.1016/j.virusres.2017.06.010 28627393

[128] Gilani U., Shaukat M., Rasheed A., Shahid M., Tasneem F., Arshad M. et al. (2018) Theimplication ofCRISPR/Cas9genomeediting technology in combating human oncoviruses. J. Med. Virol. 91, 1–13 10.1002/jmv.25292 30133783

[129] Moyo B., Bloom K., Scott T., Ely A. and Arbuthnot P. (2017) Advances with using CRISPR/Cas-mediated gene editing to treatinfections with hepatitis B virus and hepatitis C virus. Virus Res. 244, 311–320 10.1016/j.virusres.2017.01.003 28087399

[130] Paschon V., Correia F.F., Morena B.C., da Silva V.A., Dos Santos G.B., da Silva M.C.C. et al. (2020) CRISPR, Prime Editing, Optogenetics, and DREADDs: new therapeutic approaches provided by emerging technologies in the treatment of spinal cord injury. Mol. Neurobiol. 57, 2085–2100 10.1007/s12035-019-01861-w 31927725

[131] Sondhi D., Stiles K.M., De B.P. and Crystal R.G. (2017) Genetic modification of the lung directed toward treatment of human disease. Hum. Gene Ther. 28, 3–82 10.1089/hum.2016.152 27927014

[132] Villate-Beitia I., Zarate J., Puras G. and Pedraz J.L. (2017) Gene delivery to the lungs: pulmonary gene therapy for cystic fibrosis. Drug Dev. Ind. Pharm. 43, 1071–1081 10.1080/03639045.2017.1298122 28270008

[133] Xia E., Duan R., Shi F., Seigel K.E., Grasemann H. and Hu J. (2018) Overcoming the undesirable CRISPR-Cas9 expression in gene correction. Mol. Ther. Nucleic Acids 13, 699–709 30513454 10.1016/j.omtn.2018.10.015 PMC6278715

[134] Ruan J., Hirai H., Yang D., Ma L., Hou X., Jiang H. et al. (2019) Efficient gene editing at major CFTR mutation loci. Mol. Ther. Nucleic Acids 16, 73–81 30852378 10.1016/j.omtn.2019.02.006 PMC6409404

[135] Sanz D.J., Hollywood J.A., Scallan M.F. and Harrison P.T. (2017) Cas9/gRNA targeted excision of cystic fibrosiscausing deep-intronic splicing mutations restores normal splicing of CFTR mRNA. PLoS ONE 12, e0184009 10.1371/journal.pone.0184009 28863137 PMC5581164

[136] Bednarski C., Tomczak K., vom Hövel B., Weber W.M. and Cathomen T. (2016) Targeted integration of a super-exon into the CFTR locus leads to functional correction of a cystic fibrosis cell line model. PLoS ONE 11, e0161072 10.1371/journal.pone.0161072 27526025 PMC4985144

[137] Schwank G., Koo B.K., Sasselli V., Dekkers J.F., Heo I., Demircan T. et al. (2013) Functional repair of CFTR by CRISPR/Cas9 in intestinal stem cell organoids of cystic fibrosis patients. Cell Stem Cell 13, 653–658 10.1016/j.stem.2013.11.002 24315439

[138] Crane A.M., Kramer P., Bui J., Chung W.J., Li X.S., Gonzalez-Garay M.L. et al. (2015) Targeted correction and restored function of the CFTR gene in cystic fibrosis induced pluripotent stem cells. Stem Cell Rep. 4, 569–577 10.1016/j.stemcr.2015.02.005 25772471 PMC4400651

[139] Firth A.L., Menon T., Parker G.S., Qualls S.J., Lewis B.M., Ke E. et al. (2015) Functional gene correction for cystic fibrosis in lung epithelial cells generated from patient iPSCs. Cell Rep. 12, 1385–1390 10.1016/j.celrep.2015.07.062 26299960 PMC4559351

[140] Lee C.M., Flynn R., Hollywood J.A., Scallan M.F. and Harrison P.T. (2012) Correction of the ΔF508 mutation in the cystic fibrosis transmembrane conductance regulator gene by zinc-finger nuclease homology-directed repair. BioResearch 1, 99–108 10.1089/biores.2012.0218 PMC3559198 23514673

[141] Min Y.L., Bassel-Duby R. and Olson E.N. (2019) CRISPR correction of Duchenne muscular dystrophy. Annu. Rev. Med. 70, 239–255 10.1146/annurev-med-081117-010451 30379597 PMC6415693

[142] Amoasii L., Hildyard J.C.W., Li H., Sanchez-Ortiz E., Mireault A.A., Caballero D. et al. (2018) Gene editing restores dystrophin expression in a canine model of Duchenne muscular dystrophy. Science 362, 86–91 10.1126/science.aau1549 30166439 PMC6205228

[143] Nelson C.E., Wu Y., Gemberling M.P., Oliver M.L., Waller M.A., Bohning J.D. et al. (2019) Long-term evaluation of AAV-CRISPR genome editing for Duchenne muscular dystrophy. Nat. Med. 25, 427–432 10.1038/s41591-019-0344-3 30778238 PMC6455975

[144] Yang Y. and Huang Y. (2019) The CRIPSR/Cas gene-editing system-an immature but useful toolkit for experimental and clinical medicine. Anim. Model Exp. Med. 2, 5–8 10.1002/ame2.12061 31016281 PMC6431121

[145] Ormond K.E., Morlock D.P., Scholes D.T., Bombard Y., Brody L.C., Faucett W.A. et al. (2017) Human germline genome editing. Am. J. Hum. Genet. 101, 167–176 10.1016/j.ajhg.2017.06.012 28777929 PMC5544380

[146] Lea R.A. and Niakan K.K. (2019) Human germline genome editing. Nat. Cell Biol. 21, 1479–1489 10.1038/s41556-019-0424-0 31792374

[147] Long C., McAnally J.R., Selton J.M., Mireault A.A., Bassel-Duby R. and Olson E.N. (2014) Prevention of muscular dystrophy in mice by CRISPR/Cas9–mediated editing of germline DNA. Science 345, 1184–1188 10.1126/science.1254445 25123483 PMC4398027

[148] German D.M., Mitalipov S., Mishra A. and Kaul S. (2019) Therapeutic genome editing in cardiovascular diseases. J. Am. Coll. Cardiol. Basic Trans. Sci. 4, 122–131 10.1016/j.jacbts.2018.11.004 PMC6390678 30847427

[149] Cyranoski D. (2019) What’s next for CRISPR babies? Nature 566, 440–442 10.1038/d41586-019-00673-1 30809070

[150] Ma H., Marti-Gutierrez N., Park S.W., Wu J., Lee Y.L., Suzuki K. et al. (2017) Correction of a pathogenic gene mutation in human embryos. Nature 548, 413–419 10.1038/nature23305 28783728

[151] Tang L., Zeng Y., Du H., Gong M., Peng J., Zhang B. et al. (2017) CRISPR/Cas9-mediated gene editing in human zygotes using Cas9 protein. Mol. Genet. Genomics 292, 252–233 10.1007/s00438-017-1299-z 28251317

[152] Liang P., Xu Y., Zhang X., Ding C., Huang R., Zhang Z. et al. (2015) CRISPR/Cas9-mediated gene editing in human tripronuclear zygotes. Protein Cell 6, 363–372 10.1007/s13238-015-0153-5 25894090 PMC4417674

[153] Fogarty N.M.E., McCarthy A., Snijders K.E., Powell B.E., Kubikova N., Blakeley P. et al. (2017) Genome editing reveals a role for OCT4 in human embryogenesis. Nature 550, 67–73 10.1038/nature24033 28953884 PMC5815497

[154] Greely H.T. (2019) CRISPR’d babies: human germline genome editing in the ‘He Jiankui affair’. J. Law Biosci. 6, 111–183 10.1093/jlb/lsz010 31666967 PMC6813942

[155] Ranisch R. (2020) Germline genome editing versus preimplantation genetic diagnosis: Is there a case in favour of germline interventions? Bioethics 34, 60–69 10.1111/bioe.12635 31448423 PMC6973094

[156] Cross R. (2017) CRISPR’s breakthrough problem. Chem. Eng. News 95, 28–33

[157] Glass Z., Lee M. and Xu Q. (2018) Engineering the delivery system for CRISPR-based genome Editing. Trends Biotechnol. 36, 173–185 10.1016/j.tibtech.2017.11.006 29305085 PMC5801045

[158] Chew W.L. (2018) Immunity to CRISPR Cas9 and Cas12a therapeutics. Wiley Interdiscip. Rev. Syst. Biol. Med. 10, e1408 10.1002/wsbm.1408 29083112

[159] Barbalat R., Ewald S.E., Mouchess M.L. and Barton G.M. (2011) Nucleic acid recognition by the innate immune system. Annu. Rev. Immunol. 29, 185–214 10.1146/annurev-immunol-031210-101340 21219183

[160] Kim S.T., Koo T., Jee H.G., Cho H.Y., Lee G., Lim D.G. et al. (2018) CRISPR RNAs trigger innate immune responses in human cells. Genome Res. 28, 367–373 10.1101/gr.231936.117 PMC5848615 29472270

[161] Wienert B., Shin J., Zelin E., Pestal K. and Corn J.E. (2018) In vitro-transcribed guide RNAs trigger an innate immune response via the RIG-I pathway. PLoS Biol. 16, e2005840 10.1371/journal.pbio.2005840 30011268 PMC6049001

[162] Charlesworth C.T., Deshpande P.S., Dever D.P., Camarena J., Lemgart V.T., Cromer M.K. et al. (2019) Identification of preexisting adaptive immunity to Cas9 proteins in humans. Nat. Med. 25, 249–254 10.1038/s41591-018-0326-x 30692695 PMC7199589

[163] Simhardri V.L., McGill J., McMahon S., Wang J., Jiang H. and Suana Z.E. (2018) Prevalence of pre-existing antibodies to CRISPR-associated nuclease Cas9 in the USA population. Mol. Ther. Methods Clin. Dev. 10, 105–112 30073181 10.1016/j.omtm.2018.06.006 PMC6070699

[164] Chew W.L., Tabebordbar M., Cheng J.K.W., Mali P., Wu E.Y., Ng A.H.M. et al. (2016) A multifunctional AAV–CRISPR–Cas9 and its host response. Nat. Methods 13, 868–874 10.1038/nmeth.3993 27595405 PMC5374744

[165] Wagner D.L., Amini L., Wenderign D.J., Burkardt L.M., Akyüz L., Reinke P. et al. (2019) High prevalence of Streptococcus pyogenes Cas9-reactive T cells within the adult human population. Nat. Med. 25, 242–248 10.1038/s41591-018-0204-6 30374197

[166] Zhang X.H., Tee L.Y., Wang X.G., Huang Q.S. and Yang S.H. (2015) Off-target effects in CRISPR/Cas9-mediated genome engineering. Mol. Ther. Nucleic Acids 4, e264 10.1038/mtna.2015.37 26575098 PMC4877446

[167] Florea A.M. and Busselberg D. (2011) Cisplatin as an anti-tumor drug: cellular mechanisms of activity, drug resistance and induced side effects. Cancers (Basel) 3, 1351–1371 10.3390/cancers3011351 24212665 PMC3756417

[168] Gkazi S.A. (2019) Quantifying CRISPR off-target effects. Emerg. Top. Life Sci. 3, 327–334 33523136 10.1042/ETLS20180146

[169] Akcakaya P., Bobbin M.L., Guo J.A., Malagon-Lopez J., Clement K., Garcia S.P. et al. (2018) *In vivo* CRISPR editing with no detectable genome-wide off-target mutations. Nature 561, 416–419 10.1038/s41586-018-0500-9 30209390 PMC6194229

[170] Wienert B., Wyman S.K., Richardson C.D., Yeh C.D., Akcakaya P., Porritt M.J. et al. (2019) Unbiased detection of CRISPR off-targets *in vivo* using DISCOVER-Seq. Science 364, 286–289 31000663 10.1126/science.aav9023 PMC6589096

[171] Harewood L. and Fraser P. (2014) The impact of chromosomal rearrangements on regulation of gene expression. Hum. Mol. Genet. 23, R76–R82 10.1093/hmg/ddu278 24907073

[172] Hasty P. and Montagna C. (2014) Chromosomal rearrangements in cancer detection and potential causal mechanisms. Mol. Cell. Oncol. 1, e29904 10.4161/mco.29904 26203462 PMC4507279

[173] Giannoukos G., Ciulla D.M., Marco E., Abdulkerim H.S., Barrera L.A., Bothmer A. et al. (2018) UDiTaS, a genome editing detection method for indels and genome rearrangements. BMC Genomics 19, 212 10.1186/s12864-018-4561-9 29562890 PMC5861650

[174] Jung C., Hawkins J.A., Jones S.K. Jr, Xiao Y., Rybarski J.R., Dillard K.E. et al. (2017) Massively parallel biophysical analysis of CRISPR-Cas complexes on next generation sequencing chips. Cell 170, 35–47.e13 10.1016/j.cell.2017.05.044 28666121 PMC5552236

[175] Smits A.H., Ziebell F., Joberty G., Zinn N., Mueller W.F., Clauder-Munster S. et al. (2019) Biological plasticity rescues target activity in CRISPR knock outs. Nat. Methods. 16, 1087–1093 10.1038/s41592-019-0614-5 31659326

[176] Ihry R.J., Worringer K.A., Salick M.R., Frias E., Ho D., Theriault K. et al. (2018) p53 inhibits CRISPR-Cas9 engineering in human pluripotent stem cells. Nat. Med. 24, 939–946 10.1038/s41591-018-0050-6 29892062

[177] Haapaniemi E., Botla S., Persson J., Schmierer B. and Taipale J. (2018) CRISPR-Cas9 genome editing induces a p53-mediated DNA damage response. Nat. Med. 24, 927–930 10.1038/s41591-018-0049-z 29892067

[178] Newton M.D., Taylor B.J., Driessen R.P.C., Roos L., Cvetesic N., Allyjaun S. et al. (2019) DNA stretching induces Cas9 off-target activity. Nat. Struct. Mol. Biol. 26, 185–192 10.1038/s41594-019-0188-z 30804513 PMC7613072

[179] Aryal N.K., Wasylishen A.R. and Lozano G. (2018) CRISPR/Cas9 can mediate high-efficiency off-target mutations in mice *in vivo*. Cell Death. Dis. 9, 1099 10.1038/s41419-018-1146-0 30368519 PMC6204134

[180] Yamada M., Watanabe Y., Gootenberg J.S., Hirano H., Ran F.A., Nakane T. et al. (2017) Crystal structure of the minimal Cas9 from *Campylobacter jejuni* reveals the molecular diversity in the CRISPR-Cas9 systems. Mol. Cell 65, 1109–1121.e3 10.1016/j.molcel.2017.02.007 28306506

[181] Jiang F. and Doudna J.A. (2017) CRISPR-Cas9 structures and mechanisms. Annu. Rev. Biophys. 46, 505–529 10.1146/annurev-biophys-062215-010822 28375731

[182] Nishimasu H., Cong L., Yan W.X., Ran F.A., Zetsche B., Li Y. et al. (2015) Crystal structure of *Staphylococcus aureus* Cas9. Cell 162, 1113–1126 10.1016/j.cell.2015.08.007 26317473 PMC4670267

[183] Nishimasu H., Ran F.A., Hsu P.D., Konermann S., Shehata S.I., Dohmae N. et al. (2014) Crystal structure of Cas9 in complex with guide RNA and target DNA. Cell 156, 935–949 10.1016/j.cell.2014.02.001 24529477 PMC4139937

[184] Yang M.Y., Peng S.J., Sun R.R., Lin J.D., Wang N. and Chen C.L. (2018) The conformational dynamics of Cas9 governing DNA cleavage are revealed by single-molecule FRET. Cell Rep. 22, 372–382 10.1016/j.celrep.2017.12.048 29320734

[185] Slaymaker I.M., Gao L., Zetsche B., Scott D.A., Yan W.X. and Zhang F. (2016) Rationally engineered Cas9 nucleases with improved specificity. Science 351, 84–88 10.1126/science.aad5227 26628643 PMC4714946

[186] Kleinstiver B.P., Pattanayak V., Prew M.S., Tsai S.Q., Nguyen N.T., Zheng Z. et al. (2016) High-fidelity CRISPR-Cas9 nucleases with no detectable genome-wide off-target effects. Nature 529, 490–495 10.1038/nature16526 26735016 PMC4851738

[187] Chen J.S., Dagdas Y.S., Kleinstiver B.P., Welch M.M., Sousa A.A., Harrington L.B. et al. (2017) Enhanced proofreading governs CRISPR-Cas9 targeting accuracy. Nature 550, 407 10.1038/nature24268 28931002 PMC5918688

[188] Casini A., Olivieri M., Petris G., Montagna C., Reginato G., Maule G. et al. (2018) A highly specific SpCas9 variant is identified by *in vivo* screening in yeast. Nat. Biotechnol. 36, 265–271 10.1038/nbt.4066 29431739 PMC6066108

[189] Vakulskas C.A., Dever D.P., Rettig G.R., Turk R., Jacobi A.M., Collingwood M.A. et al. (2018) A high-fidelity Cas9 mutant delivered as a ribonucleoprotein complex enables efficient gene editing in human hematopoietic stem and progenitor cells. Nat. Med. 24, 1216–1224 10.1038/s41591-018-0137-0 30082871 PMC6107069

[190] Hu J.H., Miller S.M., Geurts M.H., Tang W., Chen L., Sun N. et al. (2018) Evolved Cas9 variants with broad PAM compatibility and high DNA specificity. Nature 556, 57–63 10.1038/nature26155 29512652 PMC5951633

[191] Tsai S.Q., Wyvekens N., Khayter C., Foden J.A., Thapar V., Reyon D. et al. (2014) Dimeric CRISPR RNA-guided FokI nucleases for highly specific genome editing. Nat. Biotechnol. 32, 569–576 10.1038/nbt.2908 24770325 PMC4090141

[192] Guilinger J.P., Thompson D.B. and Liu D.R. (2014) Fusion of catalytically inactive Cas9 to FokI nuclease improves the specificity of genome modification. Nat. Biotechnol. 32, 577–582 10.1038/nbt.2909 24770324 PMC4263420

[193] Mali P., Aach J., Stranges P.B., Esvelt K.M., Moosburner M., Kosuri S. et al. (2013) CAS9 transcriptional activators for target specificity screening and paired nickases for cooperative genome engineering. Nat. Biotechnol. 31, 833–838 10.1038/nbt.2675 23907171 PMC3818127

[194] Ran F.A., Hsu P.D., Lin C.Y., Gootenberg J.S., Konermann S., Trevino A.E. et al. (2013) Double nicking by RNA-guided CRISPR Cas9 for enhanced genome editing specificity. Cell 154, 1380–1389 10.1016/j.cell.2013.08.021 23992846 PMC3856256

[195] Fu Y., Sander J.D., Reyon D., Cascio V.M. and Joung J.K. (2014) Improving CRISPR-Cas nuclease specificity using truncated guide RNAs. Nat. Biotechnol. 32, 279–284 10.1038/nbt.2808 24463574 PMC3988262

[196] Bolukbasi M.F., Gupta A., Oikemus S., Derr A.G., Garber M., Brodsky M.H. et al. (2015) DNA-binding-domain fusions enhance the targeting range and precision of Cas9. Nat. Methods 12, 1150–1156 10.1038/nmeth.3624 26480473 PMC4679368

[197] Maji B., Moore C.L., Zetsche B., Volz S.E., Zhang F., Shoulders M.D. et al. (2017) Multidimensional chemical control of CRISPR-Cas9. Nat. Chem. Biol. 13, 9–11 10.1038/nchembio.2224 27820801 PMC5531067

[198] Xu H., Xiao T., Chen C.H., Li W., Meyer C.A., Wu Q. et al. (2015) Sequence determinants of improved CRISPR sgRNA design. Genome Res. 25, 1147–1157 10.1101/gr.191452.115 26063738 PMC4509999

[199] Wu X., Kriz A.J. and Sharp P.A. (2014) Target specificity of the CRISPR-Cas9 system. Quant. Biol. 2, 59–70 10.1007/s40484-014-0030-x 25722925 PMC4338555

[200] Cui Y., Xu J., Cheng M., Liao X. and Peng S. (2018) Review of CRISPR/Cas9 sgRNA design tools. Interdiscip. Sci. 10, 455–465 10.1007/s12539-018-0298-z 29644494

[201] Hendel A., Bak R.O., Clark J.T., Kennedy A.B., Ryan D.E., Roy S. et al. (2015) Chemically modified guide RNAs enhance CRISPR-Cas genome editing in human primary cells. Nat. Biotechnol. 33, 985–989 26121415 10.1038/nbt.3290 PMC4729442

[202] Yin H., Song C.Q., Suresh S., Wu Q., Walsh S., Rhym L.H. et al. (2017) Structure-guided chemical modification of guide RNA enables potent non-viral *in vivo* genome editing. Nat. Biotechnol. 35, 1179–1187 10.1038/nbt.4005 29131148 PMC5901668

[203] Ryan D.E., Taussig D., Steinfeld I., Phadnis S.M., Lunstad B.D., Singh M. et al. (2018) Improving CRISPR-Cas specificity with chemical modifications in single-guide RNAs. Nucleic Acids Res. 46, 792–803 10.1093/nar/gkx1199 29216382 PMC5778453

[204] Mir A., Alterman J.F., Hassler M.R., Debacker A.J., Hudgens E., Echeverria D. et al. (2018) Heavily and fully modified RNAs guide efficient SpyCas9-mediated genome editing. Nat. Commun. 9, 2641 10.1038/s41467-018-05073-z 29980686 PMC6035171

[205] O’Reilly D., Kartje Z.J., Ageely E.A., Malek-Adamian E., Habibian M., Schofield A. et al. (2019) Extensive CRISPR RNA modification reveals chemical compatibility and structure-activity relationships for Cas9 biochemical activity. Nucleic Acids Res. 47, 546–558 30517736 10.1093/nar/gky1214 PMC6344873

[206] Kocak D.D., Josephs E.A., Bhandarkar V., Adkar S.S., Kwon J.B. and Gersbach C.A. (2019) Increasing the specificity of CRISPR systems with engineered RNA secondary structures. Nat. Biotechnol. 37, 657–666 10.1038/s41587-019-0095-1 30988504 PMC6626619

[207] Strecker J., Ladha A., Gardner Z., Schmid-Burgk J.L., Makarova K.S., Koonin E.V. et al. (2019) RNA-guided DNA insertion with CRISPR-associated transposases. Science 365, 48–53 10.1126/science.aax9181 31171706 PMC6659118

[208] Klompe S.E., Vo P.L.H., Halpin-Healy T.S. and Sternberg S.H. (2019) Transposon-encoded CRISPR-Cas systems direct RNA-guided DNA integration. Nature 571, 219–225 10.1038/s41586-019-1323-z 31189177

[209] Guirouilh-Barbat J., Huck S., Bertrand P., Pirzio L., Desmaze C., Sabatier L. et al. (2004) Impact of the KU80 pathway on NHEJ-induced genome rearrangements in mammalian cells. Mol. Cell 14, 611–623 10.1016/j.molcel.2004.05.008 15175156

[210] Aird E.J., Lovendahl K.N., St Martin A., Harris R.S. and Gordon W.R. (2018) Increasing Cas9-mediated homology-directed repair efficiency through covalent tethering of DNA repair template. Commun. Biol. 1, 54 10.1038/s42003-018-0054-2 30271937 PMC6123678

[211] Lin S., Staahl B.T., Alla R.K. and Doudna J.A. (2014) Enhanced homology-directed human genome engineering by controlled timing of CRISPR/Cas9 delivery. Elife 3, e04766 10.7554/eLife.04766 25497837 PMC4383097

[212] Komor A.C., Kim Y.B., Packer M.S., Zuris J.A. and Liu D.R. (2016) Programmable editing of a target base in genomic DNA without double-stranded DNA cleavage. Nature 533, 420–424 10.1038/nature17946 27096365 PMC4873371

[213] Kim K., Ryu S.M., Kim S.T., Baek G., Kim D., Lim K. et al. (2017) Highly efficient RNA-guided base editing in mouse embryos. Nat. Biotechnol. 35, 435–437 10.1038/nbt.3816 28244995

[214] Ma Y., Yu L., Zhang X., Xin C., Huang S., Bai L. et al. (2018) Highly efficient and precise base editing by engineered dCas9-guide tRNA adenosine deaminase in rats. Cell Discov. 4, 39 10.1038/s41421-018-0047-9 30038797 PMC6048098

[215] Yeh W.H., Chiang H., Rees H.A., Edge A.S.B. and Liu D.R. (2018) *In vivo* base editing of post-mitotic sensory cells. Nat. Commun. 9, 2184 10.1038/s41467-018-04580-3 29872041 PMC5988727

[216] Lee H.K., Willi M., Miller S.M., Kim S., Liu C., Liu D.R. et al. (2018) Targeting fidelity of adenine and cytosine base editors in mouse embryos. Nat. Commun. 9, 4804 10.1038/s41467-018-07322-7 30442934 PMC6238002

[217] Tan J., Zhang F., Karcher D. and Bock R. (2019) Engineering of high-precision base editors for site-specific single nucleotide replacement. Nat. Commun. 10, 439 10.1038/s41467-018-08034-8 30683865 PMC6347625

[218] Liang P., Xie X., Zhi S., Sun H., Zhang X., Chen Y. et al. (2019) Genome-wide profiling of adenine base editor specificity by EndoV-seq. Nat. Commun. 10, 67 10.1038/s41467-018-07988-z 30622278 PMC6325126

[219] Zafra M.P., Schatoff E.M., Katti A., Foronda M., Breinig M., Schweitzer A.Y. et al. (2018) Optimized base editors enable efficient editing in cells, organoids and mice. Nat. Biotechnol. 36, 888–893 10.1038/nbt.4194 29969439 PMC6130889

[220] Thuronyi B.W., Koblan L.W., Levy J.M., Yeh W.-H., Zheng C., Newby G.A. et al. (2019) Continuous evolution of base editors with expanded target compatibility and improved activity. Nat. Biotechnol. 37, 1070–1079 10.1038/s41587-019-0193-0 31332326 PMC6728210

[221] Liu L.D., Huang M., Dai P., Liu T., Fan S., Cheng X. et al. (2018) Intrinsic nucleotide preference of diversifying base editors guides antibody *ex vivo* affinity maturation. Cell Rep. 25, 884–892.e3 10.1016/j.celrep.2018.09.090 30355495

[222] Zuo E., Sun Y., Wei W., Yuan T., Ying W., Sun H. et al. (2019) Cytosine base editor generates substantial off-target single-nucleotide variants in mouse embryos. Science 364, 289–292 30819928 10.1126/science.aav9973 PMC7301308

[223] Jin S., Zong Y., Gao Q., Zhu Z., Wang Y., Qin P. et al. (2019) Cytosine, but not adenine, base editors induce genome-wide off-target mutations in rice. Science 364, 292–295 30819931 10.1126/science.aaw7166

[224] Grunewald J., Zhou R., Garcia S.P., Iyer S., Lareau C.A., Aryee M.J. et al. (2019) Transcriptome-wide off-target RNA editing induced by CRISPR-guided DNA base editors. Nature 569, 433–437 10.1038/s41586-019-1161-z 30995674 PMC6657343

[225] Kim D., Kim D.E., Lee G., Cho S.I. and Kim J.S. (2019) Genome-wide target specificity of CRISPR RNA-guided adenine base editors. Nat. Biotechnol. 37, 430–435 10.1038/s41587-019-0050-1 30833658

[226] Terns M.P. (2018) CRISPR-based technologies: impact of RNA-targeting systems. Mol. Cell 72, 404–412 10.1016/j.molcel.2018.09.018 30388409 PMC6239212

[227] O'Connell M.R., Oakes B.L., Sternberg S.H., East-Seletsky A., Kaplan M. and Doudna J.A. (2014) Programmable RNA recognition and cleavage by CRISPR/Cas9. Nature 516, 263–266 10.1038/nature13769 25274302 PMC4268322

[228] Nelles D.A., Fang M.Y., O’Connell M.R., Xu J.L., Markmiller S.J., Doudna J.A. et al. (2016) Programmable RNA tracking in live cells with CRISPR/Cas9. Cell 165, 488–496 10.1016/j.cell.2016.02.054 26997482 PMC4826288

[229] Liu Y., Chen Z., He A., Zhan Y., Li J., Liu L. et al. (2016) Targeting cellular mRNAs translation by CRISPR-Cas9. Sci. Rep. 6, 29652 10.1038/srep29652 27405721 PMC4942795

[230] Batra R., Nelles D.A., Pirie E., Blue S.M., Marina R.J., Wang H. et al. (2017) Elimination of Toxic Microsatellite Repeat Expansion RNA by RNA-Targeting Cas9. Cell 170, 899e10–912e10 10.1016/j.cell.2017.07.010 28803727 PMC5873302

[231] Dugar G., Leenay R.T., Eisenbart S.K., Bischler T., Aul B.U., Beisel C.L. et al. (2018) CRISPR RNA-Dependent Binding and Cleavage of Endogenous RNAs by the Campylobacter jejuni Cas9. Mol. Cell 69, 893e7–905e7 10.1016/j.molcel.2018.01.032 29499139 PMC5859949

[232] Price A.A., Sampson T.R., Ratner H.K., Grakoui A. and Weiss D.S. (2015) Cas9-mediated targeting of viral RNA in eukaryotic cells. Proc. Natl. Acad. Sci. U. S. A. 112, 6164–6169 10.1073/pnas.1422340112 25918406 PMC4434742

[233] Rousseau B.A., Hou Z., Gramelspacher M.J. and Zhang Y. (2018) Programmable RNA Cleavage and Recognition by a Natural CRISPR-Cas9 System from Neisseria meningitidis. Mol. Cell 69, 906e4–914e4 10.1016/j.molcel.2018.01.025 29456189 PMC5889306

[234] Strutt S.C., Torrez R.M., Kaya E., Negrete O.A. and Doudna J.A. (2018) RNA-dependent RNA targeting by CRISPR-Cas9. Elife 7, 10.7554/eLife.32724 29303478 PMC5796797

[235] Sampson T.R., Saroj S.D., Llewellyn A.C., Tzeng Y.L. and Weiss D.S. (2013) A CRISPR/Cas system mediates bacterial innate immune evasion and virulence. Nature 497, 254–257 10.1038/nature12048 23584588 PMC3651764

[236] Abudayyeh O.O., Gootenberg J.S., Konermann S., Joung J., Slaymaker I.M., Cox D.B. et al. (2016) C2c2 is a single-component programmable RNA-guided RNA-targeting CRISPR effector. Science 353, aaf5573 10.1126/science.aaf5573 27256883 PMC5127784

[237] Abudayyeh O.O., Gootenberg J.S., Essletzbichler P., Han S., Joung J., Belanto J.J. et al. (2017) RNA targeting with CRISPR-Cas13. Nature 550, 280–284 10.1038/nature24049 28976959 PMC5706658

[238] Cox D.B.T., Gootenberg J.S., Abudayyeh O.O., Franklin B., Kellner M.J., Joung J. et al. (2017) RNA editing with CRISPR-Cas13. Science 358, 1019–1027 10.1126/science.aaq0180 29070703 PMC5793859

[239] Kim V.N. (2018) RNA-targeting CRISPR comes of age. Nat. Biotechnol. 36, 44–45 10.1038/nbt.4054 29319696

[240] Konermann S., Lotfy P., Brideau N.J., Oki J., Shokhirev M.N. and Hsu P.D. (2018) Transcriptome Engineering with RNA-Targeting Type VI-D CRISPR Effectors. Cell 173, 665 10.1016/j.cell.2018.02.033 29551272 PMC5910255

[241] Qi L.S., Larson M.H., Gilbert L.A., Doudna J.A., Weissman J.S., Arkin A.P. et al. (2013) Repurposing CRISPR as an RNA-guided platform for sequence-specific control of gene expression. Cell 152, 1173–1183 10.1016/j.cell.2013.02.022 23452860 PMC3664290

[242] Gilbert L.A., Larson M.H., Morsut L., Liu Z., Brar G.A., Torres S.E. et al. (2013) CRISPR-mediated modular RNA-guided regulation of transcription in eukaryotes. Cell 154, 442–451 10.1016/j.cell.2013.06.044 23849981 PMC3770145

[243] Konermann S., Brigham M.D., Trevino A., Hsu P.D., Heidenreich M., Cong L. et al. (2013) Optical control of mammalian endogenous transcription and epigenetic states. Nature 500, 472–476 10.1038/nature12466 23877069 PMC3856241

[244] Margolin J.F., Friedman J.R., Meyer W.K., Vissing H., Thiesen H.J. and Rauscher F.J. 3rd (1994) Kruppel-associated boxes are potent transcriptional repression domains. Proc. Natl. Acad. Sci. U. S. A. 91, 4509–4513 10.1073/pnas.91.10.4509 8183939 PMC43815

[245] Lupo A., Cesaro E., Montano G., Zurlo D., Izzo P. and Costanzo P. (2013) KRAB-Zinc Finger Proteins: A Repressor Family Displaying Multiple Biological Functions. Curr. Genomics 14, 268–278 10.2174/13892029113149990002 24294107 PMC3731817

[246] Gasperini M., Hill A.J., McFaline-Figueroa J.L., Martin B., Kim S., Zhang M.D. et al. (2019) A Genome-wide Framework for Mapping Gene Regulation via Cellular Genetic Screens. Cell 176, 1516 10.1016/j.cell.2019.02.027 30849375

[247] Mandegar M.A., Huebsch N., Frolov E.B., Shin E., Truong A., Olvera M.P. et al. (2016) CRISPR Interference Efficiently Induces Specific and Reversible Gene Silencing in Human iPSCs. Cell Stem Cell 18, 541–553 10.1016/j.stem.2016.01.022 26971820 PMC4830697

[248] Libby A.R., Joy D.A., So P.L., Mandegar M.A., Muncie J.M., Mendoza-Camacho F.N. et al. (2018) Spatiotemporal mosaic self-patterning of pluripotent stem cells using CRISPR interference. Elife 7, 10.7554/eLife.36045 30298816 PMC6177255

[249] Kearns N.A., Genga R.M., Enuameh M.S., Garber M., Wolfe S.A. and Maehr R. (2014) Cas9 effector-mediated regulation of transcription and differentiation in human pluripotent stem cells. Development 141, 219–223 10.1242/dev.103341 24346702 PMC3865759

[250] Zheng Y., Shen W., Zhang J., Yang B., Liu Y.N., Qi H. et al. (2018) CRISPR interference-based specific and efficient gene inactivation in the brain. Nat. Neurosci. 21, 447–454 10.1038/s41593-018-0077-5 29403034

[251] Yeo N.C., Chavez A., Lance-Byrne A., Chan Y., Menn D., Milanova D. et al. (2018) An enhanced CRISPR repressor for targeted mammalian gene regulation. Nat. Methods 15, 611–616 10.1038/s41592-018-0048-5 30013045 PMC6129399

[252] Maeder M.L., Linder S.J., Cascio V.M., Fu Y., Ho Q.H. and Joung J.K. (2013) CRISPR RNA-guided activation of endogenous human genes. Nat. Methods 10, 977–979 10.1038/nmeth.2598 23892898 PMC3794058

[253] Perez-Pinera P., Kocak D.D., Vockley C.M., Adler A.F., Kabadi A.M., Polstein L.R. et al. (2013) RNA-guided gene activation by CRISPR-Cas9-based transcription factors. Nat. Methods 10, 973–976 10.1038/nmeth.2600 23892895 PMC3911785

[254] La Russa M.F. and Qi L.S. (2015) The new state of the art: Cas9 for gene activation and repression. Mol. Cell. Biol. 35, 3800–3809 10.1128/MCB.00512-15 26370509 PMC4609748

[255] Cheng A.W., Wang H., Yang H., Shi L., Katz Y., Theunissen T.W. et al. (2013) Multiplexed activation of endogenous genes by CRISPR-on, an RNA-guided transcriptional activator system. Cell Res. 23, 1163–1171 10.1038/cr.2013.122 23979020 PMC3790238

[256] Gilbert L.A., Horlbeck M.A., Adamson B., Villalta J.E., Chen Y., Whitehead E.H. et al. (2014) Genome-scale CRISPR-mediated control of gene repression and activation. Cell 159, 647–661 10.1016/j.cell.2014.09.029 25307932 PMC4253859

[257] Tanenbaum M.E., Gilbert L.A., Qi L.S., Weissman J.S. and Vale R.D. (2014) A protein-tagging system for signal amplification in gene expression and fluorescence imaging. Cell 159, 635–646 10.1016/j.cell.2014.09.039 25307933 PMC4252608

[258] Chavez A., Scheiman J., Vora S., Pruitt B.W., Tuttle M., E P.R.I. et al. (2015) Highly efficient Cas9-mediated transcriptional programming. Nat. Methods 12, 326–328 10.1038/nmeth.3312 25730490 PMC4393883

[259] Konermann S., Brigham M.D., Trevino A.E., Joung J., Abudayyeh O.O., Barcena C. et al. (2015) Genome-scale transcriptional activation by an engineered CRISPR-Cas9 complex. Nature 517, 583–588 10.1038/nature14136 25494202 PMC4420636

[260] Peabody D.S. (1993) The RNA binding site of bacteriophage MS2 coat protein. EMBO J. 12, 595–600 10.1002/j.1460-2075.1993.tb05691.x 8440248 PMC413242

[261] Chavez A., Tuttle M., Pruitt B.W., Ewen-Campen B., Chari R., Ter-Ovanesyan D. et al. (2016) Comparison of Cas9 activators in multiple species. Nat. Methods 13, 563–567 10.1038/nmeth.3871 27214048 PMC4927356

[262] Liao H.K., Hatanaka F., Araoda T., Reddy P., Wu M.Z., Sui Y. et al. (2017) In vivo target gene activation via CRISPR/Cas9-mediated trans-epigenetic modulation. Cell 171, 1495–1507 10.1016/j.cell.2017.10.025 29224783 PMC5732045

[263] Kretzmann J.A., Evans C.W., Moses C., Sorolla A., Kretzmann A.L., Wang E. et al. (2019) Tumour suppression by targeted intravenous nonviral CRISPRa using dendritic polymers. Chem. Sci. 10, 7718–7727 10.1039/C9SC01432B 31588320 PMC6761875

[264] Matharu N., Rattanasopha S., Tamura S., Maliskova L., Wang Y., Bernard A. et al. (2019) CRISPR-mediated activation of a promoter or enhancer rescues obesity caused by haploinsufficiency. Science 363, eaau0629 10.1126/science.aau0629 30545847 PMC6570489

[265] Smith Z.D. and Meissner A. (2013) DNA methylation: roles in mammalian development. Nat. Rev. Genet. 14, 204–220 10.1038/nrg3354 23400093

[266] Wu H. and Zhang Y. (2014) Reversing DNA methylation: mechanisms, genomics, and biological functions. Cell 156, 45–68 10.1016/j.cell.2013.12.019 24439369 PMC3938284

[267] Attwood J.T., Yung R.L. and Richardson B.C. (2002) DNA methylation and the regulation of gene transcription. Cell. Mol. Life Sci. 59, 241–257 10.1007/s00018-002-8420-z 11915942 PMC11146104

[268] Siddique A.N., Nunna S., Rajavelu A., Zhang Y., Jurkowska R.Z., Reinhardt R. et al. (2013) Targeted methylation and gene silencing of VEGF-A in human cells by using a designed Dnmt3a-Dnmt3L single-chain fusion protein with increased DNA methylation activity. J. Mol. Biol. 425, 479–491 10.1016/j.jmb.2012.11.038 23220192

[269] Bernstein D.L., Le Lay J.E., Ruano E.G. and Kaestner K.H. (2015) TALE-mediated epigenetic suppression of CDKN2A increases replication in human fibroblasts. J. Clin. Invest. 125, 1998–2006 10.1172/JCI77321 25866970 PMC4463192

[270] Vojta A., Dobrinic P., Tadic V., Bockor L., Korac P., Julg B. et al. (2016) Repurposing the CRISPR-Cas9 system for targeted DNA methylation. Nucleic Acids Res. 44, 5615–5628 10.1093/nar/gkw159 26969735 PMC4937303

[271] McDonald J.I., Celik H., Rois L.E., Fishberger G., Fowler T., Rees R. et al. (2016) Reprogrammable CRISPR/Cas9-based system for inducing site-specific DNA methylation. Biol. Open 5, 866–874 10.1242/bio.019067 27170255 PMC4920199

[272] Liu X.S., Wu H., Ji X., Stelzer Y., Wu X., Czauderna S. et al. (2016) Editing DNA methylation in the mammalian genome. Cell 167, 233–247.e17 10.1016/j.cell.2016.08.056 27662091 PMC5062609

[273] Amabile A., Migliara A., Capasso P., Biffi M., Cittaro D., Naldini L. et al. (2016) Inheritable silencing of endogenous genes by hit-and-run targeted epigenetic editing. Cell 167, 219–232.e14 10.1016/j.cell.2016.09.006 27662090 PMC5039111

[274] Huang Y.H., Su J., Lei Y., Brunetti L., Gundry M.C., Zhang X. et al. (2017) DNA epigenome editing using CRISPR-Cas SunTag-directed DNMT3A. Genome Biol. 18, 176 10.1186/s13059-017-1306-z 28923089 PMC5604343

[275] Pflueger C., Tan D., Swain T., Nguyen T., Pflueger J., Nefzger C. et al. (2018) A modular dCas9-SunTag DNMT3A epigenome editing system overcomes pervasive off-target activity of direct fusion dCas9-DNMT3A constructs. Genome Res. 28, 1193–1206 10.1101/gr.233049.117 29907613 PMC6071642

[276] Stepper P., Kungulovski G., Jurkowska R.Z., Chandra T., Krueger F., Reinhardt R. et al. (2017) Efficient targeted DNA methylation with chimeric dCas9-Dnmt3a-Dnmt3L methyltransferase. Nucleic Acids Res. 45, 1703–1713 10.1093/nar/gkw1112 27899645 PMC5389507

[277] Galonska C., Charlton J., Mattei A.L., Donaghey J., Clement K., Gu H. et al. (2018) Genome-wide tracking of dCas9-methyltransferase footprints. Nat. Commun. 9, 597 10.1038/s41467-017-02708-5 29426832 PMC5807365

[278] Lin L., Liu Y., Xu F., Huang J., Daugaard T.F., Petersen T.S. et al. (2018) Genome-wide determination of on-target and off-target characteristics for RNA-guided DNA methylation by dCas9 methyltransferases. Gigascience 7, 1–19 10.1093/gigascience/giy011 PMC5888497 29635374

[279] Xiong T., Meister G.E., Workman R.E., Kato N.C., Spellberg M.J., Turker F. et al. (2017) Targeted DNA methylation in human cells using engineered dCas9-methyltransferases. Sci. Rep. 7, 6732 10.1038/s41598-017-06757-0 28751638 PMC5532369

[280] Lei Y., Zhang X., Su J., Jeong M., Gundry M.C., Huang Y.H. et al. (2017) Targeted DNA methylation *in vivo* using an engineered dCas9-MQ1 fusion protein. Nat. Commun. 8, 16026 10.1038/ncomms16026 28695892 PMC5508226

[281] Maeder M.L., Angstman J.F., Richardson M.E., Linder S.J., Cascio V.M., Tsai S.Q. et al. (2013) Targeted DNA demethylation and activation of endogenous genes using programmable TALE-TET1 fusion proteins. Nat. Biotechnol. 31, 1137–1142 10.1038/nbt.2726 24108092 PMC3858462

[282] Choudhury S.R., Cui Y., Lubecka K., Stefanska B. and Irudayaraj J. (2016) CRISPR-dCas9 mediated TET1 targeting for selective DNA demethylation at BRCA1 promoter. Oncotarget 7, 46545–46556 10.18632/oncotarget.10234 27356740 PMC5216816

[283] Morita S., Noguchi H., Horii T., Nakabayashi K., Kimura M., Okamura K. et al. (2016) Targeted DNA demethylation in vivo using dCas9-peptide repeat and scFv-TET1 catalytic domain fusions. Nat. Biotechnol. 34, 1060–1065 10.1038/nbt.3658 27571369

[284] Xu X., Tao Y., Gao X., Zhang L., Li X., Zou W. et al. (2016) A CRISPR-based approach for targeted DNA demethylation. Cell Discov. 2, 16009 10.1038/celldisc.2016.9 27462456 PMC4853773

[285] Gu T., Lin X., Cullen S.M., Luo M., Jeong M., Estecio M. et al. (2018) DNMT3A and TET1 cooperate to regulate promoter epigenetic landscapes in mouse embryonic stem cells. Genome Biol. 19, 88 10.1186/s13059-018-1464-7 30001199 PMC6042404

[286] Bannister A.J. and Kouzarides T. (2011) Regulation of chromatin by histone modifications. Cell Res. 21, 381–395 10.1038/cr.2011.22 21321607 PMC3193420

[287] Hilton I.B., D’Ippolito A.M., Vockley C.M., Thakore P.I., Crawford G.E., Reddy T.E. et al. (2015) Epigenome editing by a CRISPR-Cas9-based acetyltransferase activates genes from promoters and enhancers. Nat. Biotechnol. 33, 510–517 10.1038/nbt.3199 25849900 PMC4430400

[288] Kwon D.Y., Zhao Y.T., Lamonica J.M. and Zhou Z. (2017) Locus-specific histone deacetylation using a synthetic CRISPR-Cas9-based HDAC. Nat. Commun. 8, 15315 10.1038/ncomms15315 28497787 PMC5437308

[289] Shi Y., Lan F., Matson C., Mulligan P., Whetstine J.R., Cole P.A. et al. (2004) Histone demethylation mediated by the nuclear amine oxidase homolog LSD1. Cell 119, 941–953 10.1016/j.cell.2004.12.012 15620353

[290] Whyte W.A., Bilodeau S., Orlando D.A., Hoke H.A., Frampton G.M., Foster C.T. et al. (2012) Enhancer decommissioning by LSD1 during embryonic stem cell differentiation. Nature 482, 221–225 10.1038/nature10805 22297846 PMC4144424

[291] Martin C. and Zhang Y. (2005) The diverse functions of histone lysine methylation. Nat. Rev. Mol. Cell Biol. 6, 838–849 10.1038/nrm1761 16261189

[292] Mendenhall E.M., Williamson K.E., Reyon D., Zou J.Y., Ram O., Joung J.K. et al. (2013) Locus-specific editing of histone modifications at endogenous enhancers. Nat. Biotechnol. 31, 1133–1136 10.1038/nbt.2701 24013198 PMC3858395

[293] Kearns N.A., Pham H., Tabak B., Genga R.M., Silverstein N.J., Garber M. et al. (2015) Functional annotation of native enhancers with a Cas9-histone demethylase fusion. Nat. Methods 12, 401–403 10.1038/nmeth.3325 25775043 PMC4414811

[294] Wu H., Mathioudakis N., Diagouraga B., Dong A., Dombrovski L., Baudat F. et al. (2013) Molecular basis for the regulation of the H3K4 methyltransferase activity of PRDM9. Cell Rep. 5, 13–20 10.1016/j.celrep.2013.08.035 24095733

[295] Cano-Rodriguez D., Gjaltema R.A., Jilderda L.J., Jellema P., Dokter-Fokkens J., Ruiters M.H. et al. (2016) Writing of H3K4Me3 overcomes epigenetic silencing in a sustained but context-dependent manner. Nat. Commun. 7, 12284 10.1038/ncomms12284 27506838 PMC4987519

[296] O’Geen H., Ren C., Nicolet C.M., Perez A.A., Halmai J., Le V.M. et al. (2017) dCas9-based epigenome editing suggests acquisition of histone methylation is not sufficient for target gene repression. Nucleic Acids Res. 45, 9901–9916 10.1093/nar/gkx578 28973434 PMC5622328

